# Engineering ion migration and interface chemistry via covalent organic framework-enhanced polymer electrolytes for fast-charging sodium solid-state batteries

**DOI:** 10.1038/s41467-026-75417-7

**Published:** 2026-07-20

**Authors:** Yongbiao Mu, Chen Li, Jun Huang, Xiaoqian Xu, Yichun Wang, Quanyan Man, Zhiyu Zou, Meisheng Han, Qing Zhang, Guobin Zhang, Lei Wei, Baohua Li, Lin Zeng

**Affiliations:** 1 Shenzhen Key Laboratory of Advanced Energy Storage, Department of Mechanical and Energy Engineering, Southern University of Science and Technology, Shenzhen, China; 2 SUSTech Energy Institute for Carbon Neutrality, Southern University of Science and Technology, Shenzhen, China; 3 Department of Chemical and Biological Engineering, The Hong Kong University of Science and Technology, Hong Kong SAR, China; 4 College of New Materials and New Energies, Shenzhen Technology University, Shenzhen, China; 5 Institute of Materials Research, Tsinghua Shenzhen International Graduate School, Tsinghua University Shenzhen, Shenzhen, China

**Keywords:** Batteries, Batteries, Batteries

## Abstract

Solid-state sodium batteries offer a promising route toward safe and cost-effective energy storage, yet their practical implementation remains limited by the difficulty of coupling fast ion transport with stable electrode–electrolyte interfaces, especially under fast-charging and long-cycling conditions. Here we report a covalent organic framework/poly(sodium acrylate-co-fluorinated ethylene) (COF/PNSE) composite electrolyte developed through synergistic structural and chemical regulation. The aligned nanoporous COF provides continuous Na⁺ transport pathways while mechanically reinforcing the PNSE matrix, delivering an ionic conductivity of 1.2 mS cm^−1^ at 30 °C. This integrated electrolyte enables robust Na metal compatibility, as demonstrated by symmetric Na cells operating for 6,750 h with low polarization of 85 mV and a critical current density of 1.9 mA cm^−2^. Na | |Na_2/3_Ni_1/3_Mn_2/3_O_2_ batteries deliver 82.5 mAh g^−1^ at 1 A g^−1^ and retain 77.2% capacity after 1,000 cycles at 100 mA g^−1^, while maintaining 92.7% retention at 4.2 V over 180 cycles and 83.5% retention at 1 A g^−1^ over 2,000 cycles. Ah-level pouch cells further retain 87.3% capacity after 488 cycles at 1 A. Mechanistic analyses reveal that the COF framework guides uniform Na deposition and promotes dual-gradient NaF/Na_2_O-rich interphases, suppressing dendrite growth and stabilizing both electrodes. These findings inform future composite electrolyte design for solid-state sodium batteries.

## Abstract (web summary)

Solid-state sodium batteries are hindered by slow ion transport and unstable electrode–electrolyte interfaces. Here, the authors develop a covalent organic framework - enhanced polymer electrolyte that promotes the formation of stable dual-gradient interphases, leading to improved rate capability and cycling stability.

## Introduction

The accelerating global transition toward renewable energy integration and electrified transportation has intensified the need for next-generation energy storage systems that surpass the intrinsic limitations of conventional lithium-ion batteries^1,2^. Sodium solid-state batteries (SSSBs) represent a compelling alternative, capitalizing on sodium’s natural abundance, low cost, and electrochemical properties comparable to lithium^3,4^. Critically, SSSBs offer inherent safety benefits by replacing flammable liquid electrolytes with non-combustible solids of an essential requirement for large-scale grid storage and electric vehicle applications^5^. However, achieving high-performance SSSBs remains hindered by two fundamental challenges: (i) uncontrolled sodium dendrite growth at the metal negative electrode interface^6^, and (ii) sluggish ion transport kinetics that severely restrict fast-charging capability^7,8^. These issues are intrinsically linked to the larger ionic radius of Na⁺ (1.02 Å) compared to Li⁺ (0.76 Å), which exacerbates interfacial contact loss, elevates charge-transfer resistance, and accelerates dendrite nucleation^9^.

Polymer electrolytes—particularly poly(ethylene oxide) (PEO) derivatives—have been extensively investigated for SSSBs due to their flexibility, processability, and favorable interfacial compatibility^10,11^.Nevertheless, their room-temperature ionic conductivity seldom exceeds 10^−4^ S cm^−1^, constrained by high crystallinity and limited segmental mobility^12^. More critically, the narrow electrochemical stability window of PEO ( < 3.8 V) limits compatibility with high-voltage positive electrodes required for high-energy-density systems. Although poly(vinylidene fluoride) (PVDF)-based terpolymers offer improved anodic stability ( > 4.5 V), their chemically inert backbone exhibits negligible Na⁺ solvation capability, resulting in low transference numbers ( < 0.3) and severe concentration polarization under high-rate cycling^13,14^. To circumvent these kinetic bottlenecks, recent strategies have pivoted towards modulating the local coordination environment and hierarchical morphology within PVDF-based matrices. Specifically, the high electronegativity of fluorine atoms in the PVDF-HFP units can theoretically induce a strong dipole moment^15^; however, their poor coordinative interaction with Na^+^ ions leads to a “decoupled” transport mechanism that is often hindered by the high degree of polymer crystallinity^16^. Recent advances have demonstrated that the introduction of specific filler-matrix interactions—such as Lewis acid-base coupling^17^ or tethered anion sites^18^ can effectively reconfigure the Na^+^ hopping pathways, transitioning from slow segmental-dependent motion to fast, channel-mediated transport. These advancements underscore the necessity of a trifunctional design of combining high oxidative stability, accelerated desolvation kinetics, and uniform ion flux to realize truly viable PVDF-based solid-state sodium batteries.

To enhance ionic transport, composite polymer electrolytes (CPEs) incorporating inorganic fillers such as Al_2_O_3_, TiO_2_, NASICON-type ceramics have been developed^16^. These fillers can disrupt polymer crystallinity and promote Na-salt dissociation through Lewis acid-base interactions. However, isotropic ceramic particles tend to agglomerate, leading to discontinuous ion-conduction networks^19^. Moreover, the rigid filler-polymer interfaces can generate stress concentrators during electrode volume changes, thereby accelerating crack formation and enabling dendrite penetration^20^.

Covalent organic frameworks (COFs)—crystalline porous polymers with tunable pore architectures and chemical functionalities, have emerged as transformative fillers for advanced CPEs^21^. Unlike amorphous polymers or particulate ceramics, COFs feature long-range ordered nanopores that act as molecular conduits for rapid ion migration^22^. Their periodic scaffolds can be precisely functionalized ( − SO_3_⁻, −COO⁻) to enhance Na⁺ dissociation and mobility^23^. Furthermore, their high mechanical stiffness (Young’s modulus >10 GPa) and favorable interfacial compatibility with polymers contribute to dendrite suppression. While pioneering studies have demonstrated COF-enhanced Li-ion conductors with conductivities exceeding 1 mS cm^−1^, analogous advances for sodium systems remain limited. The larger Na⁺ size necessitates COFs with optimized pore diameters ( > 1 nm) and binding energies to minimize desolvation penalties, a design criterion unmet by most existing frameworks^24–26^. Lee et al. modified a separator with a sp^2^ carbon-conjugated COF to induce a NaF-rich SEI and achieve a high ion transference number, effectively suppressing sodium dendrites and enabling long-term cycling of sodium metal batteries at high current densities.^27^ Yousaf et al. developed a trifunctional halogenated COF-modified separator that anchors anions, lowers desolvation barriers, and induces a NaF-rich interface, enabling uniform Na^+^ flux^28^ Sadegh Kaviani et al. reviewed the frontier of COF-based solid polymer electrolytes, highlighting the synergistic integration of DFT, MD simulations, and machine learning to provide a roadmap for the rational design of next-generation metal-ion batteries^29–31^. Building upon these insights, it is imperative to develop COF structures with tailored pore environments and functional sites to further optimize ion transport and interfacial stability in sodium-based systems.

Herein, we report a hierarchically structured hybrid electrolyte comprising a sulfonate-functionalized covalent organic framework (TpPa-SO_3_Na) embedded within a high-dielectric poly(vinylidene fluoride-co-hexafluoropropylene-co-chlorotrifluoroethylene) (PNSE) terpolymer matrix. This synergistic configuration leverages the π–π stacking and negatively charged channels of the covalent organic framework for rapid sodium-ion (Na^+^) conduction, while the fluorinated matrix contributes to mechanical reinforcement and facilitates ion dissociation through polar C–F and C–Cl groups. The electrolyte delivers an ionic conductivity of 1.2 mS cm^−1^ at 30 °C and supports stable Na plating/stripping for 6750 h. In Na | |NNM cells, it demonstrates 83.5% capacity retention after 2000 cycles at 1 A g⁻¹, while 1 Ah hard carbon | |O3-NaNi_1/3_Fe_1/3_Mn_1/3_O_2_ pouch cells retain 87.3% capacity over 488 cycles. Operando analyses confirm the formation of a NaF-rich positive electrode-electrolyte interphase that suppresses transition-metal dissolution. This scalable hybridization strategy overcomes critical transport barriers for high-performance solid-state sodium batteries.

## Results and discussion

### Synthesis and structural characterization of COFs

**Fig. 1:**
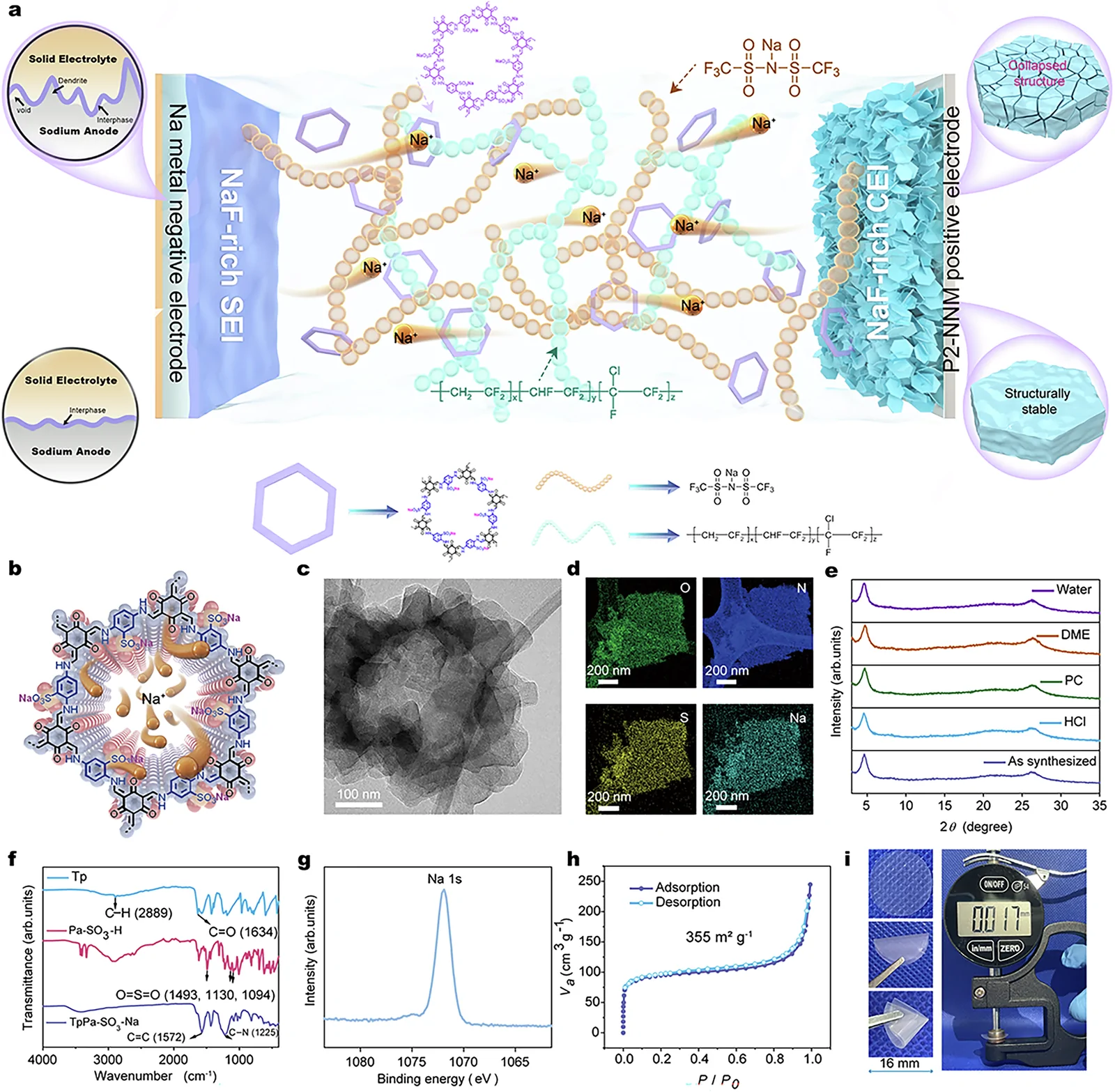
Schematic of TpPa-SO₃-Na COF-based solid-state sodium batteries and structural characterization of TpPa-SO₃-Na material. **a** Schematic illustration of the COF/PNSE composite electrolyte film in Na | |NNM solid-state batteries; **b** Structure of TpPa-SO_3_-Na material; **c** TEM image of TpPa-SO_3_-Na material; **d** the corresponding elemental mapping images; **e** XRD pattern of the TpPa-SO₃-Na after treatment with different solvents; **f** FT-IR spectrum; **g** XPS spectra of Na element; **h** Nitrogen adsorption-desorption isotherms curve; (**i**) Optical images showing mechanical flexibility and thickness measurements of the COF/PNSE composite electrolyte film.

As depicted in Fig. 1a, the mechanistic scheme of the COF/PNSE electrolyte in Na-metal solid-state batteries features a three-dimensionally interpenetrated network of COF nanochannels uniformly embedded within a poly(vinylidene fluoride-co-hexafluoropropylene-co-chlorotrifluoroethylene) (PNSE) polymer matrix. At the Na-metal negative electrode interface, the electrolyte induces the formation of a NaF-rich solid/electrolyte interphase (SEI) layer, whose high modulus effectively suppresses dendrite penetration while maintaining high Na⁺ conductivity for uniform sodium deposition. On the positive electrode side, a NaF-enriched positive electrode/electrolyte interphase (CEI) layer mitigates transition-metal dissolution from the P2-Na_2/3_Ni_1/3_Mn_2/3_O_2_ (P2-NNM) positive electrode under high-voltage operation, thereby preventing structural phase transitions and mechanical fracture of the electrode.^32^ This dual-interface engineering significantly accelerates interfacial ion transport, enabling enhanced fast-charging performance.

The TpPa-SO_3_-Na COF was designed via precise molecular engineering to exhibit hexagonally ordered pores (Fig. 1b) generated during the synthesis process (Figure S1), with densely distributed -SO_3_⁻Na⁺ groups serving as directional Na⁺ transport sites. X-ray diffraction (XRD) analysis (Figure S2) confirms the successful synthesis of TpPa-SO_3_Na, with characteristic peaks at 2θ =  4.8° and 26.9°, corresponding to the (100) and (001) planes, respectively. Scanning electron microscopy (SEM) images (Figure S3) reveal a rod-like morphology with uniform elemental distribution (Figure S4). Transmission electron microscopy (TEM) images (Fig. 1c) confirm the high crystallinity of the TpPa-SO_3_-Na, showing a 0.34 nm interlayer spacing (Figure S5) that forms continuous ionic channels for efficient Na⁺ transport within the polymer matrix. Elemental mapping (Fig. 1d) demonstrates the uniform distribution of oxygen (O), nitrogen (N), sulfur (S), and sodium (Na) elements throughout the TpPa-SO₃-Na framework.

The solvent stability of the TpPa-SO₃-Na is verified by XRD patterns (Fig. 1e), which show retention of the AA-stacked hexagonal structure, consistent with TEM results. Fourier-transform infrared (FT-IR) spectra (Fig. 1f) exhibit a C = C vibration at 1572 cm⁻^1^ and a C-N vibration at 1225 cm⁻^1^, which correlate with the (100) plane diffraction intensity in XRD, revealing the structure-activity relationship between sulfonic group density and ion conduction pathways. X-ray photoelectron spectroscopy (XPS) (Figure S6 and 1g) shows a Na 1 s peak at 1071.8 eV, confirming the presence of stable Na⁺ species within the COF channels that facilitate low-energy-barrier ion transport. Nitrogen adsorption–desorption measurements yield a BET surface area of 355 m^2^ g^−1^ (Fig. 1h) with a maintained pore size of 1.2 nm (Figure S7). When incorporated into the PNSE matrix, the porous COF structure enables fabrication of an ultrathin ( ~ 17 μm) electrolyte film (Fig. 1i). Optical images (Figure S8) show that the COF/PNSE membrane retains improved mechanical flexibility, which not only facilitates rapid ion conduction but also mitigates interfacial delamination arising from electrode volume changes^33^.

**Fig. 2:**
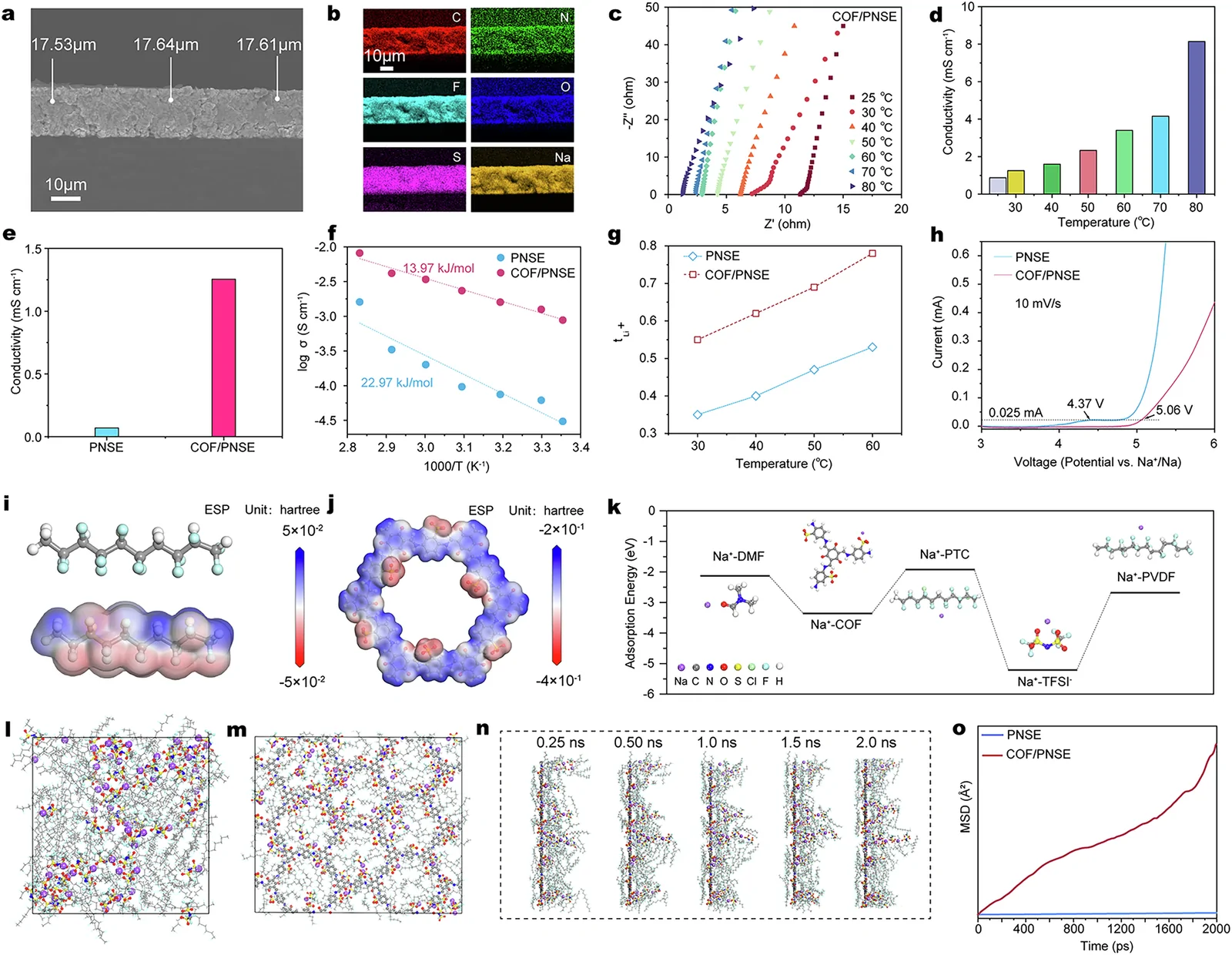
Electrochemical properties and atomistic mechanisms of Na⁺ transport in COF/PNSE electrolyte. **a** SEM image of the COF/PNSE. **b** Corresponding elemental mapping images. **c** EIS Nyquist plots of COF/PNSE at varying temperatures. **d** Temperature-dependent ionic conductivity of COF/PNSE. **e** Room-temperature ionic conductivity comparison between PNSE and COF/PNSE. **f** Activation energy (E_a_) for Na^+^ migration in PNSE and COF/PNSE. **g** Temperature-dependent Na^+^ transference numbers for PNSE and COF/PNSE. **h** LSV curves of PNSE and COF/PNSE. **i** Molecular structure and electrostatic potential distribution of PVDF-TrFE-CFE copolymer. **j** Electrostatic potential distribution of the COF framework. **k** Calculated adsorption energies of Na^+^ with DMF, COF, TFSI⁻, PTC, and PVDF. **l** MD simulation snapshots of the PNSE system. **m** MD simulation snapshots of the COF/PNSE composite system. **n** Representative simulation snapshots of Na^+^ migration pathways in the COF/PNSE system at room temperature. **o** Mean squared displacement (MSD) of Na^+^ ions in COF/PNSE and PNSE systems derived from MD simulations.

The COF/PNSE composite electrolyte exhibits enhanced ionic conductivity, selectivity, and stability, arising from its rationally engineered architecture. The composite film was fabricated via an optimized coating process, yielding a uniform morphology with interconnected networks, as evidenced by SEM imaging (Figure S9). This hierarchical structure confirms the three-dimensional integration of COF frameworks within the PNSE polymer matrix, providing continuous Na⁺ conduction channels and significantly enhancing ionic transport. The pristine PNSE electrolyte displays a similar morphology (Figure S10). Cross-sectional SEM images (Figure S11 and Fig. 2a) reveal that both PNSE and COF/PNSE membranes possess a uniform thickness of 17–20 μm. Elemental mapping (Fig. 2b, Figure S12, Figure S13) confirms the uniform distribution of C, N, F, O, S, and Na elements, indicating robust interfacial compatibility between the COF framework and polymer matrix. Such compatibility minimizes phase boundaries, ensures continuous conduction pathways, and facilitates efficient ion migration.

Thermogravimetric analysis (Figure S14) shows that TpPa-SO_3_Na exhibits significant weight loss only above 400 °C, confirming good thermal stability. Incorporating TpPa-SO_3_-Na into the PNSE matrix further improves the composite’s thermal stability. FTIR spectra (Figure S15) verify the coexistence of fluorinated polymer and COF structural features in the composite. Mechanical testing (Figure S16) reveals that while PNSE exhibits high deformability, COF incorporation markedly enhances tensile strength from 29.5 MPa to 37.6 MPa, reflecting the COF’s reinforcing effect^21^.

Electrochemical impedance spectroscopy (Fig. 2c and Figure S17) demonstrates temperature-dependent conductivity behavior, with COF/PNSE conductivity increasing from 1.25 × 10⁻^3^ S cm⁻^1^ at 30 °C to 8.14 × 10⁻^3^ S cm⁻¹ at 80 °C (Fig. 2d), surpassing pristine PNSE (7×10⁻^5^ S cm^−1^ at 30 °C; Fig. 2e, Figure S18). This enhancement originates from the ordered COF pores, which enable rapid Na⁺ hopping while suppressing anion migration. The conductivity follows the Arrhenius relationship (Fig. 2f), with COF/PNSE exhibiting a lower activation energy (E_a_ = 13.97 kJ mol^−1^) compared with PNSE (E_a_ = 22.97 kJ mol^−1^), confirming the COF’s role in lowering ion migration barriers. The Na⁺ transference number (t_Na⁺_) of COF/PNSE increases from 0.55 at 30 °C to 0.78 at 60 °C (Fig. 2g, and Figure S19), compared with the PNSE (0.35–0.53 over the same range). This improvement results from the immobilization of anions via –SO_3_⁻ groups, which enables directional Na⁺ transport, enhances selectivity, and reduces concentration polarization. Linear sweep voltammetry (Fig. 2h) reveals an expanded electrochemical stability window (5.06 V vs. Na⁺/Na) for COF/PNSE, significantly exceeding that of PNSE (4.37 V), thereby enabling compatibility with high-voltage positive electrodes.

The multi-scale ion transport kinetics and electrochemical stability of the composite electrolyte were elucidated through a combination of electrostatic potential (ESP) topography and density functional theory (DFT) calculations. As visualized in Fig. 2i–j, the PVDF-TrFE-CFE copolymer exhibits pronounced fluorine-induced electronegative regions that function as potent anchoring sites, intensifying host-guest interactions with Na⁺ cations. Intriguingly, the amorphous domains introduced by CFE moieties effectively flatten the local potential landscape, paving the way for continuous, percolated low-barrier migration pathways and surpassing the transport limits of pristine PVDF (Figure S20)^34^. Within the anionic-functionalized TpPa-SO_3_-Na framework (Figure S21), the highly ordered sulfonic acid groups generate a long-range, directional negative potential field. This structural advantage is further quantified by the energetic relay established among the electrolyte components (Fig. 2k). While the Na⁺-TFSI⁻ pair exhibits the most profound binding (−5.21 eV), the COF framework offers a strategic intermediate anchoring strength (−3.36 eV). This kinetic pivot is sufficiently robust to disrupt the primitive Na⁺-TFSI⁻ coordination for salt dissolution, yet flexible enough to facilitate facile ion exchange and hopping. Conversely, the notably weaker interaction energies of PVDF (−2.68 eV) and PTC (−1.92 eV) create an energetic exclusion zone, effectively funneling Na⁺ flux away from stagnant polymer domains and into the high-percolation COF nanochannels. These effects collectively boost ionic conductivity and ensure compatibility with high-energy positive electrode materials^35^.

Molecular dynamics (MD) simulations were performed to gain molecular-level insight into the Na⁺ diffusion kinetics in both electrolytes (Figs. 2l, 2m, and Figure S22). The results indicate that incorporating TpPa-SO_3_Na covalent organic frameworks into the PVDF-TrFE-CFE polymer matrix markedly improves NaTFSI solubility and ionic conductivity. At the initial stage, NaTFSI is uniformly distributed within the polymer matrix, with Na^+^ ions evenly coordinated to the polymer chain segments. As the simulation trajectory progresses to 0.25 ns and 0.50 ns, a distinct accumulation of Na^+^ ions gradually emerges within the COF regions, which is directly attributed to the strong electrostatic adsorption governed by the electron-withdrawing -SO_3_ groups. With the simulation extended from 1.0 ns to 2.0 ns, Na^+^ ions become substantially enriched and highly ordered around the COF sites. This long-term spatial evolution demonstrates the robust formation of continuous, polar ion-conduction channels that effectively facilitate directional Na^+^ migration and stabilize the long-range transport pathways, thereby boosting the overall ionic conductivity (Fig. 2n). In stark contrast, the COF-free PNSE benchmark exhibits persistent entropic dispersion of NaTFSI throughout the entire trajectory, where Na^+^ ions only form transient, disordered coordination with the PVDF segments without any local enrichment or structural reconfiguration. Consequently, Na^+^ transport in the benchmark is heavily confined to inefficient, stochastic hopping within the amorphous matrix, resulting in a stagnant ion flux and markedly suppressed conductivity (Figure S23). The long-range transport kinetics in both electrolyte systems were further quantified by tracking the mean square displacement (MSD) over a continuous 2000 ps timescale (Fig. 2o). The COF/PNSE system exhibits a Na⁺ diffusion coefficient of 0.388 Å^2^ ps^−1^, significantly higher than that of the PNSE system of 3.745 × 10^−3 ^Å ^2^ ps^−1^. These results confirm that the COF framework accelerates ion diffusion by providing structural reinforcement, continuous ordered channels, and directional migration pathways,^36^ thereby markedly enhancing ion transport efficiency in sodium solid-state batteries.

### High reversibility of Na/Na^+^ and uniform Na deposition

**Fig. 3:**
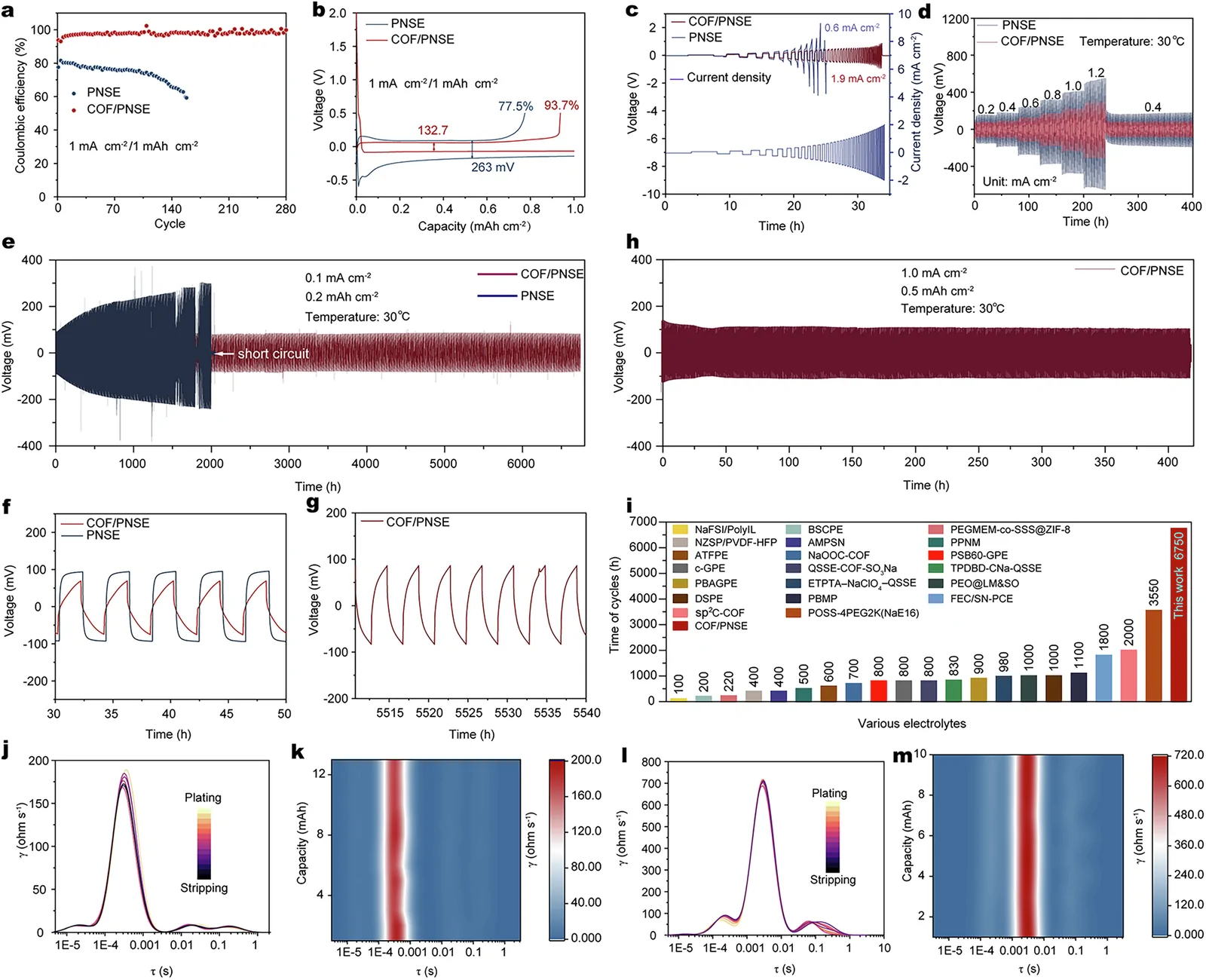
Sodium metal compatibility, interface kinetics, and long-term cycling stability. **a** Cycling stability of Na | |Cu half-cells evaluated at 1.0 mA cm^-2^/1.0 mAh cm^−2^. **b** Initial Na deposition/stripping voltage profiles for Na | |Cu cells employing PNSE and COF/PNSE electrolytes. **c** Critical current density analysis for Na | |Na solid-state cells utilizing PNSE and COF/PNSE electrolytes. **d** Rate performance of PNSE-based and COF/PNSE-based cells. **e** Long-term electrochemical stability of Na | |Na symmetric cells cycled at 0.1 mA cm^−2^/0.2 mAh cm^-2^ with PNSE and COF/PNSE. **f**, **g** Enlarged voltage-time traces of Na plating/stripping cycles from (**e**). **h** High-rate cycling capability of Na | |Na symmetric cells at 1 mA cm^-2^/0.5 mAh cm^-2^ using COF/PNSE. **i** Cycling lifespan benchmarking against representative sodium solid-state battery electrolytes reported^21–23,27,33,36,43–55^ (The literature data sources shown in this figure are provided in Supplementary Information Table S1.). **j**, **k** Distribution of Relaxation Times (DRT) analysis for Na plating and stripping kinetics in COF/PNSE-containing symmetric cells. **l**, **m** Corresponding DRT spectra for PNSE-only symmetric cells.

To comprehensively evaluate the electrochemical performance of the COF/PNSE composite solid-state electrolyte in sodium metal batteries, multidimensional characterizations were conducted to assess its interfacial stability and ion transport kinetics. Long-term cycling tests of Na | |Cu half-cells (Fig. 3a) at 1.0 mA cm^−2^ and 1.0 mAh cm^−2^ demonstrate that COF/PNSE achieves an average Coulombic efficiency (CE) of 97.8% over 280 cycles. In contrast, pure PNSE fails after 140 cycles due to short-circuiting, with CE dropping to 50% (Figure S24), highlighting the COF framework’s effectiveness in suppressing sodium dendrite growth and minimizing parasitic electrolyte decomposition. Initial Na plating/stripping voltage profiles (Fig. 3b) reveal that COF/PNSE delivers good reversibility, with a first-cycle CE of 93.7% (vs. 77.5% for PNSE) and a lower nucleation overpotential of 132.7 mV (vs. 263.0 mV). These improvements indicate that the COF’s two-dimensional conductive network reduces the energy barrier for Na⁺ deposition, enabling uniform electroplating/stripping and minimizing dead Na formation. Critical current density (CCD) measurements (Fig. 3c) further confirm COF/PNSE’s dendrite suppression capability: it sustains a CCD of 1.9 mA cm⁻^2^ with a polarization peak of 1.1 V, while PNSE fails at 0.6 mA cm^-2^ with 2.3 V polarization (Figure S25).

Rate performance testing (Fig. 3d) demonstrates the COF/PNSE electrolyte’s enhanced ion transport kinetics across 0.2–1.2 mA cm⁻^2^. At 0.2 mA cm⁻^2^, it shows a polarization voltage of 80.2 mV (vs. 157.5 mV for PNSE), which rises to 158.4 mV at 1.2 mA cm⁻^2^ (vs. 547.6 mV for PNSE; Figure S26). This performance is attributed to the ordered porous COF channels, which accelerate Na⁺ migration and maintain stability at high current densities. Long-term cycling at 0.1 mA cm⁻^2^/0.2 mAh cm⁻^2^ (Fig. 3e) shows that COF/PNSE operates for 6750 h with an average polarization of 85 mV, whereas PNSE short-circuits at 1543 h, with polarization rising from 90.5 mV to 231.4 mV within 1000 h. Magnified voltage profiles (Fig. 3f–g) reveal negligible polarization growth and suppressed interfacial side reactions for COF/PNSE, in stark contrast to PNSE’s rapid degradation. Even under harsher conditions (1.0 mA cm^−2^/0.5 mAh cm^−2^), COF/PNSE maintains a polarization of 113.3 mV for 418 h without voltage drift (Fig. 3h, Figure S27). Benchmarking against some reported sodium solid-state electrolytes (Fig. 3i, and Table S1) indicates that COF/PNSE achieves appreciable improvements in cycle life and rate performance. By leveraging in situ electrochemical impedance spectroscopy (EIS) coupled with distribution of relaxation times (DRT) deconvolution (Fig. 3j–m), we gained fundamental insights into the interfacial kinetics. During continuous Na deposition at 0.2 mA cm⁻^2^, the COF/PNSE configuration exhibited a spontaneous decline in interfacial resistance from 375 Ω to 334 Ω (Figure S28b), a trend that stands in stark contrast to the resistive growth (from 1,410 Ω to 1,460 Ω) observed in the pristine PNSE system (Figure S28a). The DRT spectra effectively resolved the multifaceted electrochemical response into discrete timescales; specifically, the relaxation peaks identified at 10^1^–10^3 ^Hz and below 10^0 ^Hz were attributed to the charge-transfer (R_ct_) and ion diffusion (R_diff_) processes, respectively. Our analysis reveals that COF incorporation significantly suppresses the R_ct_ intensity, providing tangible evidence of a lower desolvation energy barrier and accelerated interfacial Na^+^ exchange kinetics. In a sharp departure from this behavior, PNSE alone suffers from broadened and intensified relaxation peaks, indicative of slow interfacial kinetics and the stochastic buildup of resistive species. Furthermore, the temporal stability of the DRT signatures for COF/PNSE over cycling underscores the formation of a highly uniform and robust SEI. These results establish COF/PNSE as a paradigm-shifting electrolyte design, effectively harmonizing mechanical reinforcement with ultrafast Na⁺ transport to circumvent the long-standing bottlenecks in high-energy-density solid-state sodium batteries^23^.

### Structure and formation mechanism of the solid electrolyte interface

The highest occupied molecular orbital (HOMO) and lowest unoccupied molecular orbital (LUMO) reflect the electron-donating and electron-accepting capabilities of molecules, respectively. A lower HOMO energy level improves the oxidative stability of the electrolyte at the positive electrode, while a lower LUMO level facilitates molecular reduction at the negative electrode, thereby influencing SEI formation (Figure S29).^37^ Specifically, the Na⁺-TFSI complex exhibits the lowest LUMO level ( − 1.92 eV) among all evaluated species, rendering it highly susceptible to preferential reductive decomposition upon initial contact with the Na-metal negative electrode. This selective reduction triggers the in-situ growth of a mechanically robust, NaF/Na_2_O-rich inner SEI layer, which serves as a potent barrier against dendrite proliferation. Simultaneously, the TFSI⁻-PTC complex presents an elevated LUMO level (0.83 eV), architecting a high reductive barrier that shields the polymer host from spontaneous degradation, thereby ensuring long-term interfacial stability. This thermodynamic shielding is synergistically coupled with the narrow HOMO-LUMO gap of the Na⁺-COF complex ( ~ 1.76 eV), which promotes regulated electronic interactions and the formation of a flexible, organic/inorganic-hybrid (Na_2_O-rich) outer SEI buffer. Furthermore, the TFSI⁻-COF complex ( ~ 1.80 eV gap) functions as a molecular-scale charge-transfer bridge, accelerating Na^+^ ion hopping across the heterogeneous interphase and substantially lowering interfacial impedance.

**Fig. 4:**
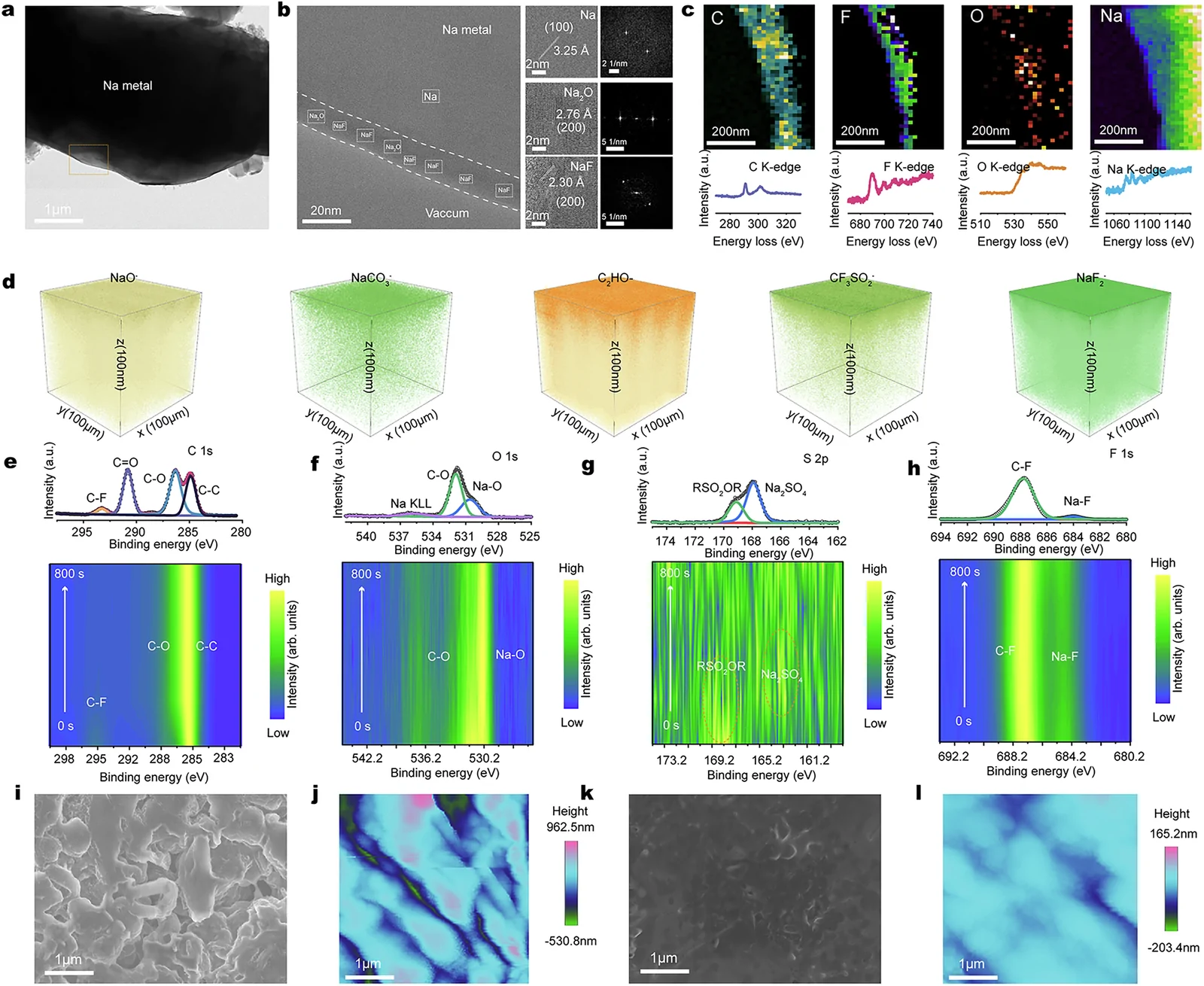
Microstructural and chemical evolution of the SEI and Na deposition morphology. **a** Low-magnification cryo-EM image of deposited Na metal. **b** HRTEM images and corresponding FFT patterns of the SEI formed with COF/PNSE electrolyte. **c** EELS mapping images and line scans across the C, F, O, and Na K-edges. **d** TOF-SIMS depth profiles of NaO⁻, NaCO_3_⁻, C_2_HO⁻, CF_3_SO_3_⁻, and NaF_2_⁻ fragments at the Na metal surface after cycling with COF/PNSE. **e**–**h** Depth-profile XPS analysis of cycled Na metal: **e** C 1 s, (**f**) O 1 s, (**g**) S 2p, (**h**) F 1 s spectra. **i** SEM image and (**j**) corresponding topography via AFM for Na metal cycled with PNSE. **k**, **l** SEM and AFM images for Na metal cycled with COF/PNSE electrolyte.

Cryogenic TEM (Fig. 4a) reveals that in the COF/PNSE system, sodium deposits as dense, uniform nanoparticles without dendritic protrusions, owing to the spatial confinement of COF’s ordered porous framework, which guides lateral Na⁺ deposition and suppresses vertical dendrite growth. In contrast, PNSE yields elongated, dendritic structures (Figure S30). High-resolution TEM (Fig. 4b) shows that COF/PNSE induces a unique amorphous-nanocrystalline composite SEI: a ~ 15 nm fluorine-rich amorphous organic matrix acts as a mechanical buffer to accommodate volume changes, while embedded 2-5 nm Na_2_O/NaF nanocrystals provide fast ion-conduction channels. PNSE instead forms a fully amorphous SEI exceeding 30 nm, with inferior mechanical integrity.

Electron energy loss spectroscopy (EELS) (Fig. 4c) and elemental mapping reveal a gradient-functionalized SEI: an outer C/F-rich layer blocks electrolyte penetration, an intermediate O-rich layer forms an ion-conductive network, and an inner layer tightly contacts the Na metal. This architecture reflects the synergistic enrichment of organic matrices with fluorine/oxygen-containing nanocrystals. TOF-SIMS depth profiling (Fig. 4d) confirms the vertical chemical gradient—Na_2_O and NaF_2_⁻ are concentrated near the Na surface and decrease outward, CF_3_SO_3_⁻ resides in the outer layer, and NaCO_3_⁻/C_2_HO⁻ species from PTC-sodium interactions permeate the SEI, enhancing toughness and stability.^38^ In contrast, PNSE-cycled sodium shows limited F_2_⁻ content, excess NaO⁻, and no clear gradient (Figure S31), indicating an uneven SEI detrimental to cycling stability.

X-ray photoelectron spectroscopy (XPS, Fig. 4e–h) depth profiles quantify SEI composition and bonding evolution. C 1 s spectra reveal a surface organic-rich layer with C-F signals localized near the top, decreasing upon etching. O 1 s and F 1 s spectra confirm an optimized inorganic/organic oxide ratio and high fluorinated SEI stability. S 2p spectra detect Na_2_SO_4_ species, which facilitate Na⁺ conduction (Fig. 4g). The gradient reduction of F/O content corroborates the fluorine/oxygen-rich outer SEI structure. In comparison, PNSE forms an SEI with weaker NaF signals (Figure S32). Morphological analysis via SEM and AFM (Fig. 4i–l) further illustrates post-cycling sodium deposition differences. PNSE produces rough, dendrite-laden surfaces ( > 2 μm fragments, >1 μm height variation; Figure S33), whereas COF/PNSE yields dense, smooth deposits ( < 300 nm height variation; Figure S34), directly evidencing COF-guided lateral deposition with the increasing of deposited capacity (Figure S35). Collectively, these results confirm the “structure-chemistry” synergy of COF/PNSE electrolytes: structurally, the COF’s ordered pores confine Na⁺ flux for uniform deposition; chemically, fluorinated groups coordinate with PNSE decomposition products to build a gradient SEI; and kinetically, the COF’s conjugated backbone lowers charge-transfer resistance and accelerates Na⁺ transport. This integrated mechanism underpins the composite’s interfacial stability and cycling durability.

### Electrochemical performance and kinetic analysis of sodium metal solid-state batteries

**Fig. 5:**
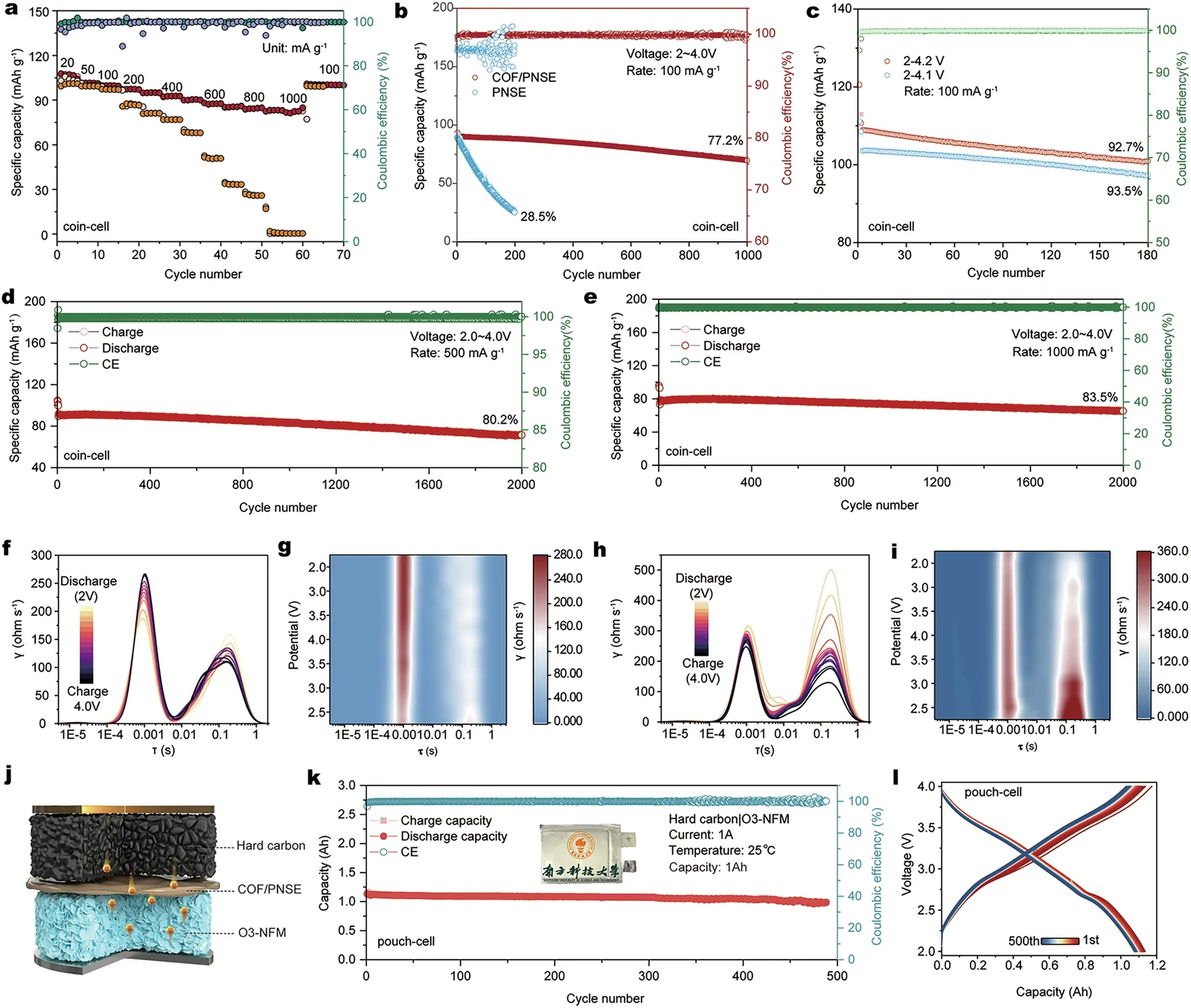
Full-cell electrochemical performance, kinetic analysis, and practical pouch cell. **a** Rate capability of Na | |NNM cells utilizing COF/PNSE and PNSE electrolytes at current densities from 20 mA g^-1^ to 1 A g^-1^. **b** Long-term cycling stability of Na | |NNM cells with COF/PNSE and PNSE electrolytes at 100 mA g^-1^ within 2.0–4.0 V. **c** Voltage window extension study for Na | |NNM cells with COF/PNSE electrolyte at 2.0–4.1 V and 2.0–4.2 V. **d**, **e** high-rate cycling performance of Na | |NNM cells with COF/PNSE at 500 mA g^-1^ (**d**) and 1 A g^-1^ (**e**). **f**, **g** DRT analysis revealing charge and discharge kinetic differences in COF/PNSE-based cells. **h**, **i** Corresponding DRT spectra for PNSE-only Na | |NNM cells. (**j**) Schematic illustration of the HC | | COF/PNSE | | O3-NFM full-cell configuration. **k** Cycling stability of Ah-level pouch cells under 1 A operation. **l** Representative charge/discharge voltage curves for the pouch cell.

To assess the full-cell performance of PNSE and COF/PNSE electrolytes, rate capability tests for Na | |NNM cells were conducted over current densities from 20 mA g^-1^ to 1 A g^-1^ (Fig. 5a and S36). The COF/PNSE system, benefiting from its synergistic structure–chemistry design, exhibits markedly improved rate performance, delivering ~99.8 mAh g^−1^ at 100 mA g^-1^ and retaining 82.5 mAh g^−1^ even at 1 A g^-1^ (Figure S37b). In contrast, PNSE retains only ~58.0 mAh g^−1^ at 500 mA g^-1^ and drops to 25.9 mAh g^−1^ at 800 mA g^-1^, with charge/discharge profiles showing pronounced polarization and capacity loss at high rates (Figure S37a). This improvement is attributed to the dual role of the COF framework: (i) its two-dimensional ordered pores shorten Na^+^ diffusion pathways and homogenize ion flux, mitigating concentration polarization, and (ii) fluorinated moieties within the COF skeleton cooperate with PNSE-derived SEI species to construct a gradient, stable electrode–electrolyte interface that supports rapid Na^+^ transport under high current densities^27^.

Long-term cycling at 100 mA g^-1^ within 2.0–4.0 V (Fig. 5b) further highlights the performance advantage. The COF/PNSE cell maintains 77.2% of its initial capacity (93.7 mAh g^−1^) with a Coulombic efficiency of 99.7% after 1000 cycles, whereas PNSE suffers severe degradation, retaining only 28.5% of its capacity after 199 cycles (Figure S38). This stability is ascribed to COF/PNSE’s ability to form fluorinated organic matrices that passivate the negative electrode surface and embed nanocrystals to create percolating ion channels, thereby preserving rapid interfacial Na^+^ transport. Leveraging its wide electrochemical stability window (confirmed by LSV), the cell also sustains stable cycling at high cutoff voltages: at 4.2 V, it retains 92.7% capacity after 180 cycles, and at 4.1 V, 93.5% retention is achieved (Fig. 5c). The kinetic advantages of COF/PNSE are further demonstrated under extreme rates. Long-term cycling at 500 mA g^-1^ and 1 A g^-1^ (Figs. 5d, 5e) shows capacity retentions of 80.2% over 2000 cycles at 500 mA g^-1^ and 83.5% at 1 A g^-1^. This performance arises from the COF’s conjugated structure, which reduces the activation energy for Na^+^ migration, and its ordered porous framework, which integrates the interfacial stability of solid-state electrolytes with the fast kinetics of liquid systems. Operando DRT deconvolution (Fig. 5f–i) effectively resolves the kinetic disparities between the two systems, identifying characteristic responses for bulk Na⁺ diffusion (*t* ~ 0.1–1 s) and interfacial charge-transfer. In the COF/PNSE configuration, the marked suppression of low-frequency impedance signals a radical acceleration in diffusion kinetics through the electrolyte framework (Figure S39b). In stark contrast, the PNSE-only cells exhibit amplified peak intensities and volatile frequency migrations (Fig. 5h–i, Figure S39a), symptomatic of sluggish charge-transfer across a highly resistive and structurally unstable CEI at the positive electrode interface. The scalability of this design is demonstrated in Fig. 5j–l. A 1 Ah pouch cell (O3-NFM | | COF/PNSE | | HC) fabricated via casting and curing achieves 87.3% capacity retention after 488 cycles at 1 A (Fig. 5k) with negligible polarization growth (Fig. 5l). SEM analysis of the cycled hard carbon electrode (Figure S40) shows a uniform surface morphology with minimal roughness changes, indicating stable electrode/electrolyte interfaces during cycling, beneficial for both performance and longevity. This scalability is underpinned by the composite’s mechanical robustness, high Coulombic efficiency, and resistance to interfacial degradation in large-format cells (Table S2). This structural/electrolyte design delivers high-energy, long- life, and high-rate sodium metal batteries with performance metrics that surpass current state-of-the-art systems.

### Evolution of positive electrode interface and structural stability

**Fig. 6:**
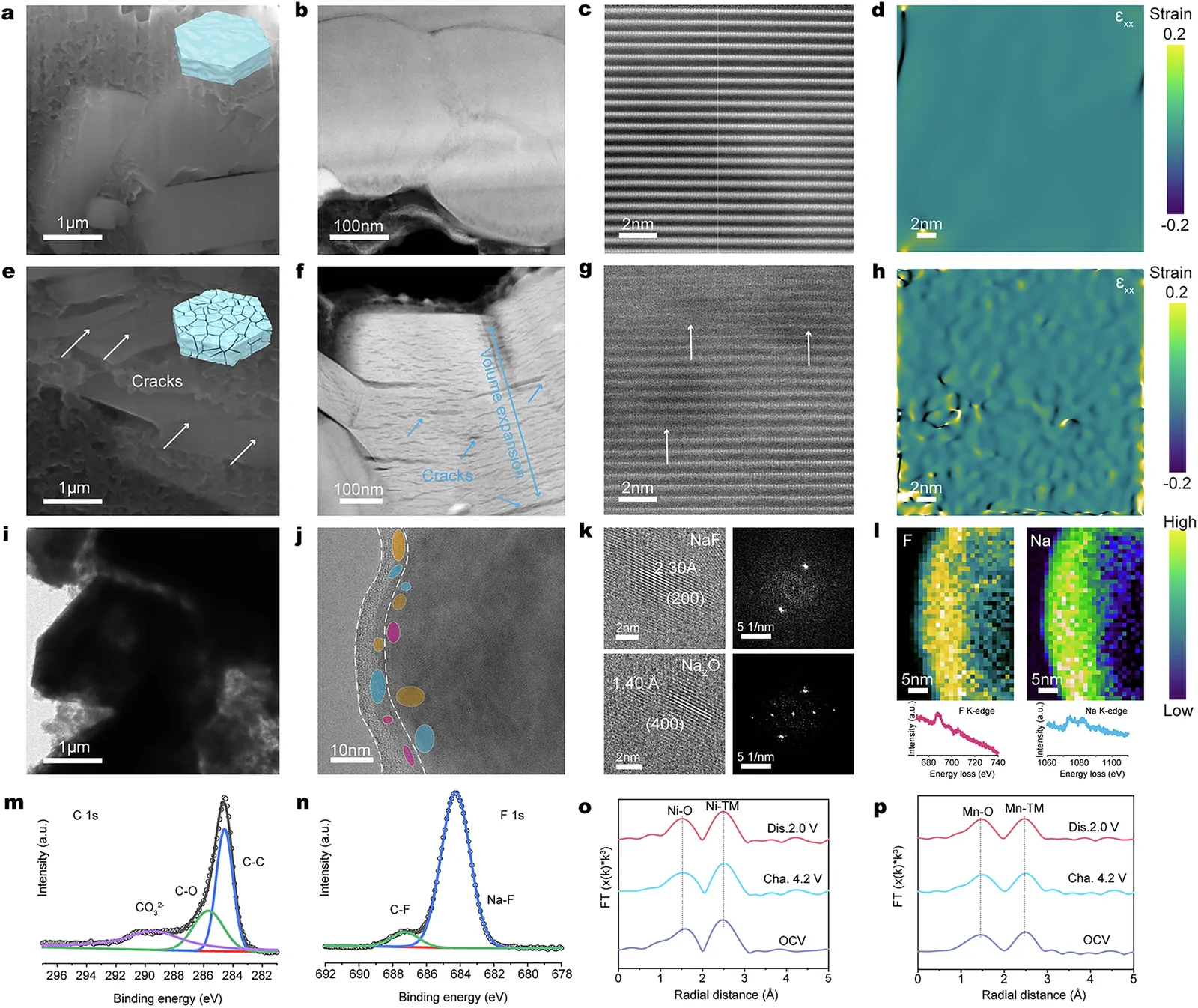
Interfacial stability, stress evolution, and CEI chemistries at the positive electrode NNM/COF/PNSE interface. **a** SEM image of NNM electrodes cycled with COF/PNSE electrolyte (Inset: Schematic of a single NNM particle structure.). **b** FIB-SEM image revealing the structural evolution. **c** TEM image and (**d**) corresponding GPA strain map of the NNM positive electrode. **e**–**h** SEM, FIB-SEM, TEM, and GPA strain mapping for NNM electrodes cycled with PNSE electrolyte (Inset: Schematic of a single NNM particle with cracks after cycling.). **i** Cryo-TEM image of the cycled NNM with COF/PNSE. **j** HRTEM image of the positive electrode-electrolyte interphase, the elliptical regions indicate different inorganic phases: yellow represents NaF, blue represents Na_2_O phase, and red regions represent amorphous areas. **k** Atomic-resolution TEM analysis of CEI nanostructure. **l** Elemental mapping via TEM-EDS and EELS line scans across F K-edge and Na K-edge transitions. XPS depth profiles for (**m**) C 1 s and (**n**) F 1 s spectra at the cycled NNM interface. XAS curves for (**o**) Ni K-edge and (**p**) Mn K-edge transitions at various charging/discharging states.

The electrolyte/ positive electrode interface plays a decisive role in the design and optimization of solid-state batteries.^20^ Here, we investigate the post-cycling structural integrity and interfacial characteristics of P2-NNM positive electrode paired with COF/PNSE and PNSE electrolytes. SEM imaging (Fig. 6a, e) reveals pronounced morphological differences: the COF/PNSE-cycled positive electrode maintains sharp particle contours, intimate interparticle contact, and a crack-free surface (Fig. 6a, Figure S41), whereas the PNSE-cycled positive electrode exhibits a roughened surface, fragmented particles, and propagating microcracks (Fig. 6e, and Figure S42). Focused ion beam SEM (FIB-SEM), TEM, and geometric phase analysis (GPA) strain mapping of the COF/PNSE-cycled electrode (Fig. 6b-d) confirm preserved lattice integrity, minimal lattice distortion, and uniform strain distribution, indicative of effective mechanical stress dissipation. These features correlate with the cell’s good long-term cycling stability and high-rate performance. In contrast, low-magnification TEM image of the PNSE-cycled electrode (Fig. 6f) reveals extensive particle cracking throughout the electrode. High-resolution TEM  and the GPA images (Fig. 6g, h) reveal severe lattice distortion, localized strain concentration, and extensive grain boundary defects, indicating structural degradation that hinders Na⁺ transport.

Cryogenic TEM was employed to resolve the nanoscale positive electrode–electrolyte interphase formed between COF/PNSE and P2-NNM (Fig. 6i–k). Low-magnification images (Fig. 6i) show intact NNM particles with well-defined contours. High-resolution images (Fig. 6j, k) reveal a continuous bilayer CEI comprising an outer ~10 nm amorphous fluorine-rich layer and an inner layer embedded with NaF/Na_2_O nanocrystals. This gradient architecture suppresses transition-metal dissolution and stabilizes the interface during cycling. TEM-based elemental mapping and EELS analysis (Fig. 6l) corroborate F enrichment in the outer CEI ( ~ 15 nm thick) and Na permeation throughout, forming an “outerly sparse, innerly dense” ionic screening layer that facilitates interfacial ion conduction. Operando FTIR spectroscopy was employed to decipher the dynamic ion-regulation mechanism within the Na | |COF/PNSE | | NNM batteries (Figure S43). By correlating the structural fingerprints of the pristine COF with the evolving spectra of the composite electrolyte, we observed that the COF skeletal vibrations (1580–1620 cm^-1^) remain remarkably constant, confirming its robust structural integrity. Concurrently, the reversible, periodic intensity modulations of the sulfonate (1080 cm^-1^) and polar C-N (1250 cm^-1^) modes provide direct spectroscopic evidence of a coordinated transport relay: the sulfonate groups anchor anions to facilitate quasi-single-ion conduction, while the C-N sites function as a molecular “conveyor belt” via dynamic coordination/de-coordination. Crucially, the characteristic –CF_2_– stretching vibrations (1150–1250 cm^-1^) of the PVDF matrix exhibit highly reversible evolution without any degradation-related stray peaks. This underscores that the COF-driven nanoconfinement effect effectively shields the polymer host from chaotic decomposition, thereby stabilizing the coordination environment and lowering the Na⁺ migration barrier under real operating conditions.

XPS profiling (Fig. 6m, n) further elucidates the CEI’s chemical composition. Deconvolution of the C 1 s spectrum reveals multiple carbonaceous fragments, indicating the presence of organic components that enhance interfacial stability and mechanical robustness. With increasing sputtering depth, C-F and C-O signals in the C 1 s spectrum decrease, confirming the outer-layer enrichment of fluorinated species. The F 1 s spectrum shows a decreasing NaF/C-F ratio from the outer to the inner CEI, with NaF dominating, indicating substantial NaF formation at the interface. EDS mapping (Figure S44) further confirms the co-distribution of carbon- and fluorine-rich species within the CEI. To further decode the chemical architecture of the positive electrode electrolyte interphase (CEI), XPS depth-profiling (Figure S45) was executed, revealing a well-defined gradient distribution of C, F, and O species as a function of etching depth. At the topmost interface, the C 1 s and O 1 s signatures are predominantly governed by organic moieties originating from electrolyte decomposition. Upon deeper penetration into the interphase, these organic-related signals progressively diminish, a transition accompanied by the relative intensification of inorganic constituents. Notably, the F 1 s spectra reveal that inorganic fluoride species, specifically NaF-based fragments, persist even within the deeper sub-layers, providing tangible evidence for the formation of a dense, F-rich protective shield. he spatial distribution of C⁻, O⁻, and F⁻ species was further visualized through 2D TOF-SIMS mapping (Figure S46), which confirms a spatially uniform and continuous enrichment of these characteristic fragments across the positive electrode/electrolyte interface. X-ray absorption spectroscopy (XAS) (Fig. 6o, p) was used to probe the transition-metal oxidation states of cycled P2-NNM. At 4.2 V, the Ni K-edge (Fig. 6o) and Mn K-edge (Fig. 6p) spectra of COF/PNSE-cycled P2-NNM closely match those of pristine P2-NNM, reflecting the highly reversible local coordination environment (TM-O and TM-TM scattering paths) and the robust structural stability of the positive electrode.^39^ Upon discharge to 2.0 V, COF/PNSE confines the Ni/Mn oxidation state changes within the reversible range, effectively preventing rock-salt phase formation and Mn^2+^ dissolution. Collectively, these results demonstrate that COF/PNSE forms a gradient-structured, dual-functional CEI at the P2-NNM positive electrode interface. Structurally, this CEI dissipates mechanical stress and preserves lattice integrity; chemically, it suppresses transition-metal dissolution and interfacial degradation; functionally, it maintains rapid Na⁺ transport across the interface. This engineered interface underpins the enhanced structural stability, electrochemical reversibility, and long-term cycling performance of COF/PNSE-based sodium solid-state batteries.

In summary, we have engineered a structurally integrated COF/PNSE composite electrolyte that redefines the performance limits of sodium-metal batteries through a synergistic “architecture-chemistry” optimization. By embedding a periodically ordered COF framework within a PNSE matrix, we successfully surmount the triple bottlenecks of dendrite proliferation, interfacial instability, and sluggish cation transport. The COF/PNSE architecture exhibits good electrochemical resilience: achieving a critical current density of 1.9 mA cm^−2^ and maintaining improved 6,750 h symmetric-cell stability with minimal polarization (85 mV). In Na | |NNM batteries, the electrolyte enables an extraordinary high-rate capability (82.5 mAh g^-1^ at 1 A g^-1^) and sustains 83.5% capacity after 2000 cycles, fundamentally outclassing the pristine PNSE baseline. Mechanistically, the COF nanopores spatially regulate Na^+^ flux, fostering the in-situ growth of a robust, gradient interphase enriched with NaF/Na_2_O nanocrystals. The practical viability is confirmed in Ah-level pouch cells, which retain 87.3% capacity over 488 cycles. Overall, the COF/PNSE platform provides a scalable method for next-generation, high-energy-density sodium-metal storage systems.

## Methods

Synthesis of TpPa-SO₃Na COF:

The TpPa-SO_3_-H COF was synthesized via a Schiff-base condensation reaction. Under a nitrogen atmosphere, a Pyrex tube reactor was prepared with 1,3,5-triformylphloroglucinol (Tp, Aladdin, purity ≥98%) (0.6 mmol, 126.0 mg) and 2,5-diaminobenzenesulfonic acid (Pa-(SO_3_H, Aladdin, purity ≥98% (HPLC))) (0.9 mmol, 169.4 mg). A 6 mL mixed solvent of 1,4-dioxane (Aladdin, purity ≥98%) and mesitylene (Aladdin, purity ≥98%) (v/v, 1:4) was added, along with 1.2 mL of 6 M acetic acid (Aladdin, purity ≥98%) as a catalyst. The mixture underwent sonication for 10 min, followed by flash freezing in liquid nitrogen and degassing through three freeze-pump-thaw cycles, before being maintained at 120 °C for a duration of three days. The resultant TpPa-SO_3_-H COF was collected by filtration, washed with tetrahydrofuran (THF) (Aladdin, purity ≥99%, AR), deionized water, and ethanol, and dried under vacuum at 120 °C overnight, resulting in a yield of 255.2 mg (95%). The synthesis of the TpPa-SO_3_-Na COF involved an ion exchange process. In this procedure, 240 mg of TpPa-SO_3_-H COF powder was combined with 40 mL of a 5 M sodium acetate aqueous solution and allowed to stir for three days at room temperature. After this period, the resulting TpPa-SO_3_-Na COF was filtered and subsequently washed with deionized water to eliminate any residual sodium acetate. Finally, the product was dried under vacuum at 120 °C overnight, yielding 219.4 mg (85%).

Synthesis of PNSE and COF/PNSE:

PNSE: 0.5 g of sodium bis(trifluoromethane)sulfonimide (NaTFSI, Aladdin Reagent, ≥99.95%) was precisely weighed and dissolved in a N,N-dimethylformamide (DMF, Aladdin Reagent, ≥98.5%) solution of poly(vinylidene fluoride-trifluoroethylene-chlorotrifluoroethylene) (PVDF-TRFE-CTFE, Mw=500,000, Aladdin Reagent). Under argon protection, the mixture was continuously stirred magnetically at 500 rpm for 5 h to form a homogeneous and transparent precursor polymer solution. An automatic doctor blade coater (MSK-AFA-III, Hefei Kejing) was employed to uniformly coat the solution onto a polytetrafluoroethylene (PTFE) substrate, with the coating thickness precisely controlled at 50 ± 5 μm. The coated wet film was immediately transferred to a vacuum oven (BZF-50, Shanghai Yiheng) and dried under vacuum at 80 °C for 12 h to thoroughly remove residual solvents. The dried electrolyte membrane was then punched into standard circular disks using a custom-designed punch (diameter 16 mm, thickness 20 μm). The resulting samples were sealed and stored in an argon-filled glovebox ( < 0.1 ppm H_2_O/O_2_, Mikrouna) for subsequent use.

COF/PNSE: 100 mg of synthesized COF material was added to the aforementioned precursor solution, with all remaining steps maintained identical to the PNSE electrolyte preparation protocol.

Positive Electrode Fabrication:

P2-type NNM (sodium nickel manganese oxide, P2-Na_2/3_Ni_1/3_Mn_2/3_O_2_) positive electrode sheets were prepared via slurry casting. NNM material, conductive additive (Super P), and binder (PVDF) were mixed in a weight ratio of 8:1:1, then dispersed in N-methyl-2-pyrrolidone (NMP) to form a homogeneous slurry using a THINKY MIXER. The slurry was coated onto aluminum foil current collectors, followed by vacuum drying at 80 °C for 12 h. Dried electrodes were punched into 12 mm-diameter disks and thoroughly dried in an argon-filled glove box prior to use. The positive electrode was single-side coated. The NNM positive electrode active material mass loading was controlled between 1.0–1.5 mg cm^−2^.

Battery Assembly:

CR2032 coin cells (Na | |electrolyte | |NNM) and sodium symmetric cells (Na | |electrolyte | |Na) were assembled in an argon-filled glove box. Sodium metal (purity 99.9%, Jiaxing Changgao New Materials Technology Co., Ltd) was used as the electrode. Sodium metal with a purity of ≥99.7%, a diameter of 15.6 mm, and a thickness of 0.45–0.5 mm were used. The outer oxide layer was carefully removed inside an Ar-filled glovebox (H_2_O < 0.1 ppm, O_2_ < 0.1ppm) to reveal a fresh, shiny surface. The 2032 coin-cell cases were purchased from Dongguan Kelude Co., Ltd. During cell assembly, one stainless-steel spacer with a thickness of 1.0 mm, one stainless-steel spacer with a thickness of 0.5 mm, and one stainless-steel spring were used to ensure close contact among the cell components. The positive and negative electrodes were separated by a solid-state electrolyte film with a diameter of 16 mm and a thickness of ~20 μm. No additional liquid electrolyte was added. The assembled coin cells were sealed using an MSK-110 compact hydraulic coin-cell crimping machine, for which the pressure gauge was set to ~60 kg cm^−2^. For 1.0 Ah pouch cells (hard carbon | |electrolyte | |O3-NFM), the 1 Ah solid state battery used O3-NFM as the positive electrode material and hard carbon as the negative electrode. The cell was assembled in a stacked configuration with a charge/discharge voltage range of 2–4 V. The polymer electrolyte was directly cast onto the surfaces of both the positive and negative electrodes, and the cells were assembled by stacking after solvent evaporation. Cell assembly was carried out in a glovebox. The positive electrode consisted of 95.3% active material, PVDF, SP, NMP, and CNT, while the hard carbon negative electrode consisted of 94.5% active material, water, CMC, SP, and SBR. The N/P ratio of the cell was 1.5. The positive electrode sheets had a size of 60 × 80 mm with 8 layers, and the negative electrode sheets had a size of 63 × 84 mm with 9 layers. The testing pressure was 0.5 MPa. After resting, the cell was fully charged and then subjected to cycling tests.

Materials Characterization:

Sample morphology was analyzed using a Hitachi SU-8250 field emission scanning electron microscope (SEM). Transmission electron microscopy (TEM) combined with energy-dispersive X-ray spectroscopy (EDX) and elemental mapping was performed on a Talos instrument (Thermo Fisher) operating at 300 kV. For nanoscale structural analysis of sodium metal negative electrode solid electrolyte interphase (SEI) and cathode electrode electrolyte interphase (CEI), cryogenic electron microscopy (Cryo-EM) was employed: cycled electrodes were rapidly cryo-fixed in liquid nitrogen for cooling, followed by cryogenic dark-field imaging on the Talos system. Elemental distribution (Na, O, F) was mapped using electron energy loss spectroscopy (EELS). Crystalline phase identification was conducted via X-ray diffraction (XRD) using a Bruker D8 ADVANCE Ultima IV system equipped with a D/teX Ultra detector, employing Cu-Kα radiation (λ = 1.5418 Å) at a scanning rate of 10°/min. Powder X-ray diffraction (PXRD) patterns of the COF materials were recorded on a PANalytical X-ray diffractometer (Cu Kα radiation λ = 1.54056 Å, Model X’pert Pro), with a scan rate of 2° min^–1^ across a 2-theta range of 3 to 35°. X-ray photoelectron spectroscopy (XPS) and depth-etching analysis were performed on a Thermo Scientific ESCALAB 250Xi platform using Al Kα radiation (hν = 1486.8 eV) as the excitation source. To facilitate direct comparison across different etching depths, a baseline offset was applied to the XPS spectra to eliminate background fluctuations, which strictly preserves relative peak areas and spectral contours. Infrared spectra were recorded using a Bruker Vertex 70 v Fourier transform infrared spectrometer (FTIR). Thermal transition temperatures of electrolytes and decomposition profiles of polymers/sodium salts were obtained via simultaneous thermal analysis (DSC/TG, STA449F3). Surface morphology and roughness of cycled sodium metal negative electrodes were characterized using atomic force microscopy (AFM, MFP-3D Stand Alone). Focused ion beam-scanning electron microscopy (FIB-SEM, FEI Helios 5UX) was employed for micro-region processing and cross-sectional morphology analysis of O3-NFM and P2-NNM positive electrodes. X-ray absorption fine structure (XAFS) measurements at the Ni and Mn K-edges were performed at the BL13SSW beamline of the Shanghai Synchrotron Radiation Facility (SSRF) in transmission mode.

Electrochemical Measurements:

The ionic conductivities of solid-state electrolytes were evaluated via electrochemical impedance spectroscopy (EIS) using a Biologic VSP-300 workstation. Temperature-dependent measurements were conducted from 25 °C to 80 °C with frequency scans ranging from 10 MHz to 0.01 Hz. Ionic conductivity (σ) was calculated using:

$$\,\sigma=\frac{L}{R\times S}$$

where *σ* is the ionic conductivity, L is the thickness of the electrolyte, R is resistance, S is the contact area between electrolyte and stainless steel (SS).

The activation energy *E*_*a*_ is calculated through Arrhenius equation:

$$\sigma \,=A\exp \left(\frac{{E}_{a}}{{k}_{b}T}\right)$$

where *A* is the pre-exponential factor and *k*_*b*_ is the Boltzmann constant.

The linear sweep voltammetry (LSV) curves were collected using Na | |SS cells at 0–6 V (vs Na/Na^+^) with a scan rate of 1 mV s^-1^.

The sodium-ion transference numbers of the electrolytes were determined using DC polarization and electrochemical impedance spectroscopy (EIS) measurements in Na | |Na symmetric cells, and calculated via the Bruce-Vincent-Evans (BVE) equation:

$${t}_{{{Na}}^{+}}=\frac{{I}_{{ss}}(\Delta V-{I}_{0}{R}_{0})}{{I}_{0}(\Delta V-{I}_{{ss}}{R}_{{ss}})}$$

where ΔV is the applied potential (10 mV), ${I}_{0}$ and ${I}_{{ss}}$ are the initial and steady-state current, and ${R}_{0}$ and ${R}_{{ss}}$ are initial interfacial impedance, steady-state interfacial impedance, respectively.

Electrochemical Performance:

Na | |Na symmetric cells were assembled by sandwiching self-developed PNSE and covalent organic framework composite electrolytes (COF/PNSE) between commercial sodium metal plates (12 mm diameter, 100 μm thickness) in CR2032 coin cells. Na-Cu half-cells were constructed using sodium metal negative electrodes (12 mm diameter), copper foil negative electrode (16 mm diameter), and PNSE/COF-PNSE electrolytes. Na | |NNM batteries employed sodium metal plates (15.6 mm diameter) as negative electrodes paired with NNM positive electrodes (12 mm diameter, 1 ~ 1.5 mg cm^−2^ loading) and various solid-state electrolyte systems. The Na | |NNM batteries were charged and discharged between 2.0 V and 4.0 V (1 C = 100 mAh g^-1^). The cycling stability of sodium-ion batteries was assessed within two voltage windows: 2.0 ~ 4.1 V and 2.0 ~ 4.2 V. All the electrochemical performance were carried out at room temperature. All electrochemical evaluations were conducted on a Neware battery test system (Shenzhen, China). Symmetric cells underwent galvanostatic cycling at 0.2-1 mA cm^-2^ current densities with 0.2-1 mAh cm^-2^ fixed capacity to assess cycling stability and voltage hysteresis. Electrochemical impedance spectroscopy (EIS) and in-situ EIS measurements were performed using a Solartron 1470E electrochemical workstation across 100 kHz to 100 mHz frequency ranges. During electrochemical testing, both Na metal symmetric cells and Na | |P2-NNM batteries were tested with at least three replicates, and representative data showing an intermediate performance level were selected for presentation in the manuscript. The scientific graphs were plotted using OriginPro.

Theoretical simulation:

The DFT calculations were carried out using a plane-wave basis within the projector augmented wave (PAW) method^40^. The exchange–correlation energy was described by the Perdew–Burke–Ernzerhof (PBE)^41^ functional under the generalized gradient approximation, and dispersion interactions were included through the DFT-D3 correction^42^. A kinetic energy cutoff of 450 eV was applied for the plane-wave expansion. The convergence criteria were set to 10^−5^ eV for electronic self-consistency and 0.02 eV Å^−1^ for structural relaxation. The computational models were constructed according to the formal ionic states of the electrolyte components, in which Na^+^ and TFSI^−^ were treated as charge-balanced ion pairs to preserve overall electroneutrality during geometry optimization and electronic structure analysis. For the MD simulations, the COMPASS III force field was adopted to describe the polymer/organic framework-based electrolyte system. The charges of Na^+^ and TFSI^−^ were explicitly assigned as +1 and −1, respectively, in the simulation cell. This charge assignment was used to describe long-range electrostatic interactions in the MD simulations while remaining consistent with the ionic configurations considered in the DFT models.

## Supplementary information

Supplementary Information

Description of Additional Supplementary Files

Supplementary Data 1

Transparent Peer Review file

## Source data

Source Data

## Data Availability

The data that support the findings of this study are available from the corresponding author upon request. Source data are provided with this paper.
