# Supplementary material for: Engineering ion migration and interface chemistry via covalent organic framework-enhanced polymer electrolytes for fast-charging sodium solid-state batteries
Source: Nat Commun. 2026 Jul 20;17:8850. doi: 10.1038/s41467-026-75417-7 (PMC13500708; doi:10.1038/s41467-026-75417-7)
Supplement: Supplementary file 1 [file 41467_2026_75417_MOESM2_ESM.pdf]

Description of Additional Supplementary Files

Supplementary Data 1. The source files of DFT calculation and MD simulation structure
