# Supplementary material for: Engineering ion migration and interface chemistry via covalent organic framework-enhanced polymer electrolytes for fast-charging sodium solid-state batteries
Source: Nat Commun. 2026 Jul 20;17:8850. doi: 10.1038/s41467-026-75417-7 (PMC13500708; doi:10.1038/s41467-026-75417-7)
Supplement: Supplementary file 2 [file 41467_2026_75417_MOESM4_ESM.pdf]

nature portfolio

Peer Review File

Engineering Ion Migration and Interface Chemistry via COF-Enhanced Polymer Electrolytes for Fast-Charging Sodium Solid-State Batteries

Corresponding Author: Professor Baohua Li

This file contains all reviewer reports in order by version, followed by all author rebuttals in order by version.

Version 0:

Reviewer comments:

Reviewer #1

(Remarks to the Author)
The manuscript “Engineering Ion Migration and Interface Chemistry via COF-Enhanced Polymer Electrolytes for Fast-Charging Sodium Solid-State Batteries” presents a solid and well-structured study that integrates materials engineering, interfacial chemistry, and electrochemical testing to design high-performance COF/PNSE composite electrolytes for sodium solid-state batteries. The results, including high Na^+ conductivity, large Na^+ transference number, excellent cycling stability, and high critical current density, are strong and scientifically significant. However, before acceptance, one methodological point requires clarification: in the DFT simulation section, the authors report using the GGA-BLYP functional in DMol³, but they do not justify why BLYP was chosen over more standard hybrid functionals (e.g., B3LYP) or more robust GGA-level approaches (e.g., PBE), nor do they explain the omission of essential van der Waals dispersion corrections such as Grimme’s D3 (e.g., BLYP-D3), which are critically important for accurate modeling of COF-based systems where noncovalent interactions dominate. In addition, the introduction would be strengthened by integrating recent and relevant literature on COF-based polymer electrolytes and DFT-driven battery materials design, specifically: (1) S. Kaviani, Energy Storage Materials (2025), DOI: 10.1016/j.ensm.2025.104671; (2) A. Shamsieva et al., Computational and Theoretical Chemistry (2025), DOI: 10.1016/j.comptc.2025.115445; and (3) S. Kaviani et al., Colloids and Surfaces A (2025), DOI: 10.1016/j.colsurfa.2024.135752

Reviewer #2

(Remarks to the Author)
Zeng et al. reported a covalent organic framework/poly(sodium acrylate-co-fluorinated ethylene) copolymer (COF/PNSE) composite electrolyte, which achieved high ionic conductivity and robust interfacial stability in solid-state sodium metal batteries through a synergistic "structure-chemistry" optimization strategy, while enabling fast-charging capability. The electrolyte exhibited a critical current density of 1.9 mA cm^{-2}, and the Na||NFM full cell delivered a discharge capacity of 82.5 mAh g^{-1} at 10 C, with 77.2% capacity retention after 1,000 cycles at 1 C and 83.5% retention after 2,000 cycles at 10 C. Extensive interfacial characterization revealed the interfacial chemical phenomena at the interfaces between the electrolyte, sodium metal anode, and NFM cathode during cycling. The study presents rich data with strong logical coherence, and the electrolyte design and characterization robustly support the main findings. I recommend the manuscript for minor revisions prior to acceptance and publication in Nature Communications. Specific comments are as follows:
1. The text size in some figures (e.g., Figure 1a, 1f, 2n, 3b, 3c, 3d, 4b) is too small and should be enlarged. Specific conductivity values should be labeled in Figure 2d. Clarify which elements the atoms in Figures 2i and 2j represent.
2. Enrich and refine the review of PVDF-based sodium-ion electrolyte advancements in the background section.
3. Investigate the periodic black shadows in Figures 3d and 3h, verify their origin, and ensure image clarity.
4. Strengthen the analysis of in-situ EIS and DRT (Distribution of Relaxation Times) data, explicitly indicating resistance value changes.
5. Supplement cryo-electron microscopy sample preparation methods for anode/cathode in the Supporting Information (SI) file, including detailed parameters.
6. Annotate etched XPS data (e.g., Figure 4e) with specific corresponding species/fragments. Describe electrode materials, processing details, and assembly protocols for Figures 5j-5l in the SI.
7. Analyze depth-dependent elemental (C, F, O, etc.) distribution at the cathode interface using XPS and TOF-SIMS (Time-

of-Flight Secondary Ion Mass Spectrometry) for cross-validation.

Reviewer #3

(Remarks to the Author)
This manuscript reports a COF-enhanced polymer electrolyte for fast-charging sodium solid-state batteries, claiming simultaneously high ionic conductivity, ultralong cycling stability, excellent interfacial robustness, and scalability to Ah-level pouch cells. While the topic is of interest, the current manuscript raises serious concerns regarding credibility, mechanistic rigor, and scientific positioning.

First, the performance metrics reported are exceptionally high for a polymer-based sodium solid-state electrolyte (e.g., ~1.2 mS cm^{-1} at 25 °C, >6,700 h Na||Na cycling, stable operation at 10 C for thousands of cycles, and Ah-level pouch cells). These results, taken together, are far beyond the current state of the art, yet they are supported by limited statistical validation. Key data lack reproducibility analysis, error bars, and multi-cell verification, making the robustness of the conclusions questionable.

Second, the mechanistic interpretation—particularly regarding COF-directed formation of gradient SEI/CEI layers—is largely speculative. Most evidence is based on ex situ post-mortem characterization, and alternative explanations (e.g., dominant decomposition of fluorinated polymer and salt chemistry) are not critically discussed. As a result, cause–effect relationships are overstated.

Third, the computational analysis (DFT and MD) lacks sufficient rigor and internal consistency. Simulation details are incomplete, conclusions are largely qualitative, and there are apparent inconsistencies in ion-species descriptions, which further undermine confidence in the mechanistic claims.

Finally, the conceptual novelty is limited. COF–polymer composite electrolytes have been widely explored, and the manuscript does not clearly articulate what fundamentally distinguishes this system from prior reports beyond incremental performance improvements.

In summary, despite the extensive experimental data, the manuscript does not meet the standards required for publication due to concerns over data reliability, overinterpretation of mechanisms, and limited novelty. I therefore recommend rejection of the manuscript in its current form.

Version 1:

Reviewer comments:

Reviewer #1

(Remarks to the Author)
The authors have adequately addressed all critical points raised during the review process. The manuscript is now suitable for publication.

Reviewer #2

(Remarks to the Author)
Previous questions have been addressed. It is now recommended for acceptance.

Reviewer #3

(Remarks to the Author)
The authors have made important revisions and have addressed my previous concerns in a satisfactory manner. The overall quality of the manuscript has improved, and I have no further major comments.

Open Access This Peer Review File is licensed under a Creative Commons Attribution 4.0 International License, which permits use, sharing, adaptation, distribution and reproduction in any medium or format, as long as you give appropriate credit to the original author(s) and the source, provide a link to the Creative Commons license, and indicate if changes were made.
In cases where reviewers are anonymous, credit should be given to 'Anonymous Referee' and the source.
The images or other third party material in this Peer Review File are included in the article's Creative Commons license, unless indicated otherwise in a credit line to the material. If material is not included in the article's Creative Commons license and your intended use is not permitted by statutory regulation or exceeds the permitted use, you will need to obtain permission directly from the copyright holder.
To view a copy of this license, visit https://creativecommons.org/licenses/by/4.0/

Response to the Referees' Comments

Dear reviewers,

Thank you for your precious time to constructive comments on our manuscript entitled **"Engineering Ion Migration and Interface Chemistry via COF-Enhanced Polymer Electrolytes for Fast-Charging Sodium Solid-State Batteries"** (Manuscript ID: **NCOMMS-25-80543**) for **Nature Communications**. We sincerely appreciate your comments and suggestions on our work, which are highly important for further improvements to our manuscript. According to all the comments, we have made a detailed response and substantial revisions in our revised manuscript.

Your comments are shown in black and our responses are shown in blue. The changes in the manuscript are indicated in red.

Reviewer #1 (Remarks to the Author)

The manuscript "Engineering Ion Migration and Interface Chemistry via COF-Enhanced Polymer Electrolytes for Fast-Charging Sodium Solid-State Batteries" presents a solid and well-structured study that integrates materials engineering, interfacial chemistry, and electrochemical testing to design high-performance COF/PNSE composite electrolytes for sodium solid-state batteries. The results, including high Na^+ conductivity, large Na^+ transference number, excellent cycling stability, and high critical current density, are strong and scientifically significant. However, before acceptance, one methodological point requires clarification: in the DFT simulation section, the authors report using the GGA-BLYP functional in $DMol^3$, but they do not justify why BLYP was chosen over more standard hybrid functionals (e.g., B3LYP) or more robust GGA-level approaches (e.g., PBE), nor do they explain the omission of essential van der Waals dispersion corrections such as Grimme's D3 (e.g., BLYP-D3), which are critically important for accurate modeling of COF-based systems where noncovalent interactions dominate. In addition, the introduction would be strengthened by integrating recent and relevant literature on COF-based

polymer electrolytes and DFT-driven battery materials design, specifically: (1) S. Kaviani, Energy Storage Materials (2025), DOI: 10.1016/j.ensm.2025.104671; (2) A. Shamsieva et al., Computational and Theoretical Chemistry (2025), DOI: 10.1016/j.comptc.2025.115445; and (3) S. Kaviani et al., Colloids and Surfaces A (2025), DOI: 10.1016/j.colsurfa.2024.135752.

[Author's Response]: We sincerely appreciate the time and effort you have dedicated to reviewing our manuscript, as well as the insightful and constructive feedback you have provided. Your comments have been invaluable in improving both the quality and clarity of our work. We have carefully addressed each of the issues raised and have made the necessary revisions to the manuscript.

The detailed point-to-point responses are as follows:

1) One methodological point requires clarification: in the DFT simulation section, the authors report using the GGA-BLYP functional in DMol³, but they do not justify why BLYP was chosen over more standard hybrid functionals (e.g., B3LYP) or more robust GGA-level approaches (e.g., PBE), nor do they explain the omission of essential van der Waals dispersion corrections such as Grimme's D3 (e.g., BLYP-D3), which are critically important for accurate modeling of COF-based systems where noncovalent interactions dominate.

[Author's Response]:

We thank the reviewer for this important methodological comment regarding the choice of exchange–correlation functional and the inclusion of dispersion corrections.

(1) In response, we have re-performed all DFT calculations using the GGA-PBE functional and explicitly included Grimme's D3 dispersion correction (PBE-D3) to properly account for van der Waals interactions that are critical in COF-based systems. The "DFT simulation" section has been revised accordingly,

and the corresponding updated methods, recalculated results and references have been added in the revised manuscript, as detailed below:

Quantum chemical calculations were conducted using density functional theory (DFT). All DFT computations were performed employing the DMol3 method [1]. This method utilizes rapidly convergent three-dimensional numerical integrations to compute matrix elements within the Ritz variational approach. A double numerical basis set with polarization functions on all atoms except hydrogen, known as the DND basis set, was utilized. For geometry optimizations, the projector augmented wave (PAW) method was used to represent the ion–core electron interactions[2]. The valence electrons were represented with a plane wave basis set with an energy cutoff of 450 eV. Electronic exchange and correlation were described with the Perdew–Burke–Ernzerhof (PBE) functional[3]. DFT-D3 method was used to treat the van der Waals interaction [4]. The convergence criteria for the self-consistent electronic structure and geometry were set to 10^{-5} eV and 0.02 eV/Å, respectively.

References:

(1) Delley, B. DMol3 DFT studies: from molecules and molecular environments to surfaces and solids. *Computational Materials Science* **2000**, *17* (2), 122-126. DOI: https://doi.org/10.1016/S0927-0256(00)00008-2.

(2) Kresse, G.; Joubert, D. From ultrasoft pseudopotentials to the projector augmented-wave method. *Physical Review B* **1999**, *59* (3), 1758-1775. DOI: 10.1103/PhysRevB.59.1758.

(3) Perdew, J. P.; Burke, K.; Ernzerhof, M. Generalized gradient approximation made simple. *PHYSICAL REVIEW LETTERS* **1996**, *77* (18), 3865-3868, Article. DOI: 10.1103/PhysRevLett.77.3865.

(4) Grimme, S.; Antony, J.; Ehrlich, S.; Krieg, H. A consistent and accurate ab initio parametrization of density functional dispersion correction (DFT-D) for the 94 elements H-Pu. *The Journal of Chemical Physics* **2010**, *132* (15). DOI: 10.1063/1.3382344 (acccessed 6/22/2023).

(2) The DFT-related calculations have been revised as follows:

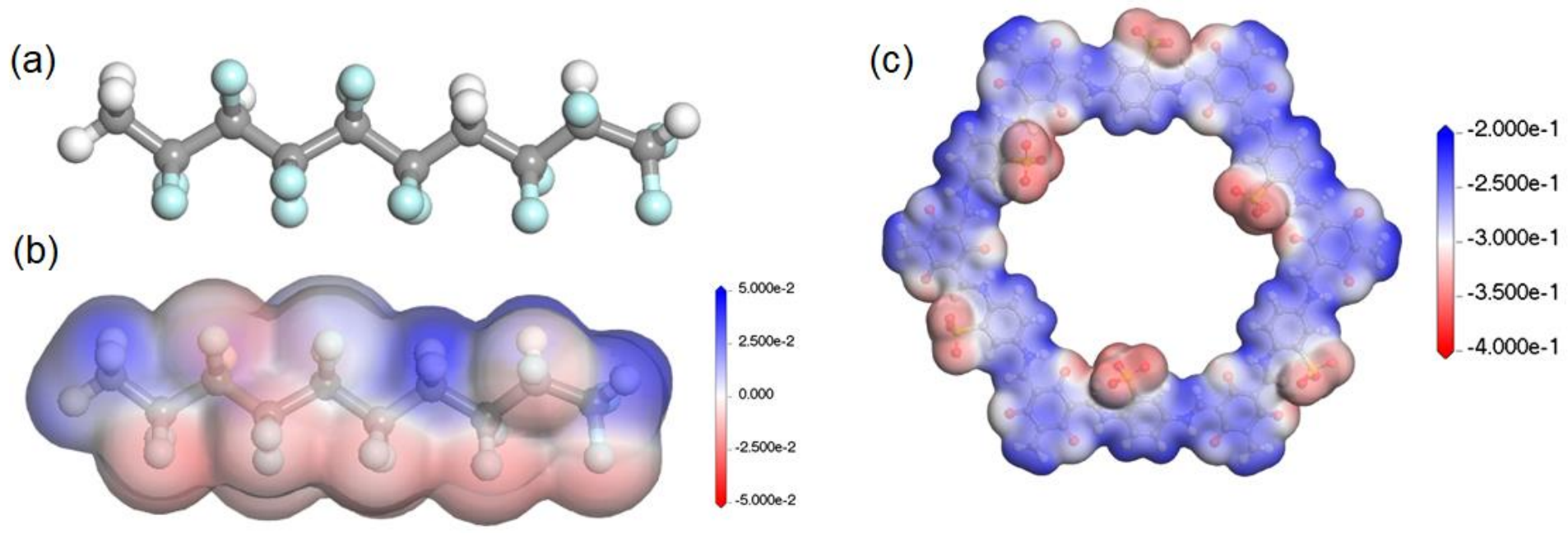

Fig. R1 (a) Molecular structure and (b) electrostatic potential distribution of PVDF-TrFE-CFE copolymer. (c) Electrostatic potential distribution of the COF framework.

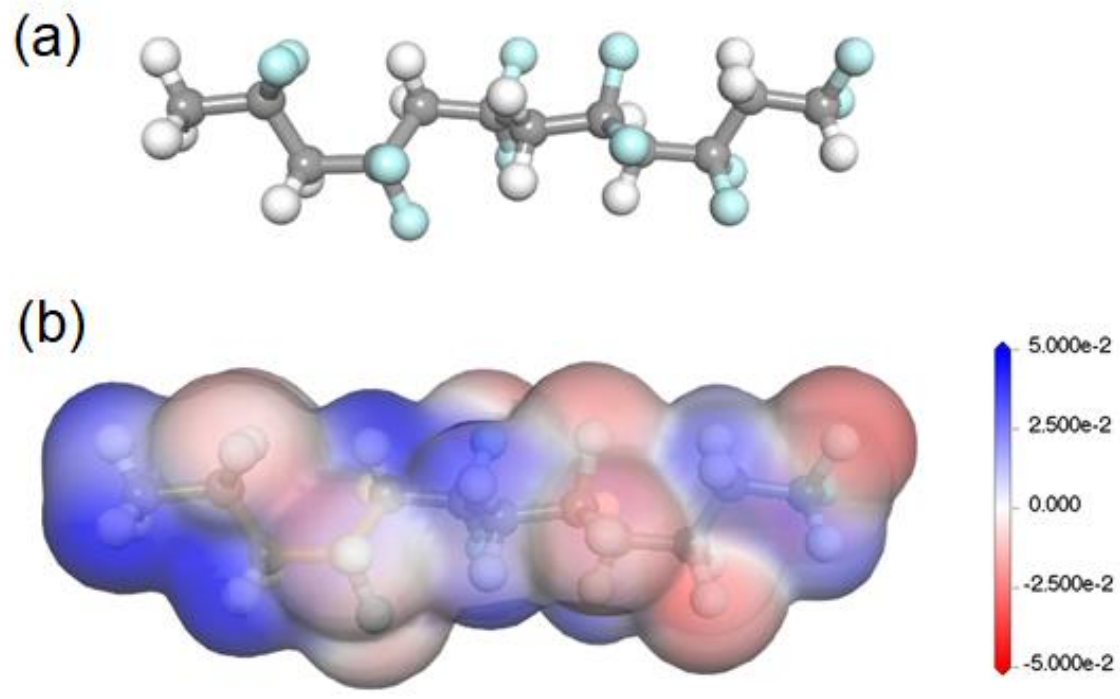

Fig. R2. (a) The ball-and-stick model of PVDF and (b) the electrostatic potential of PVDF.

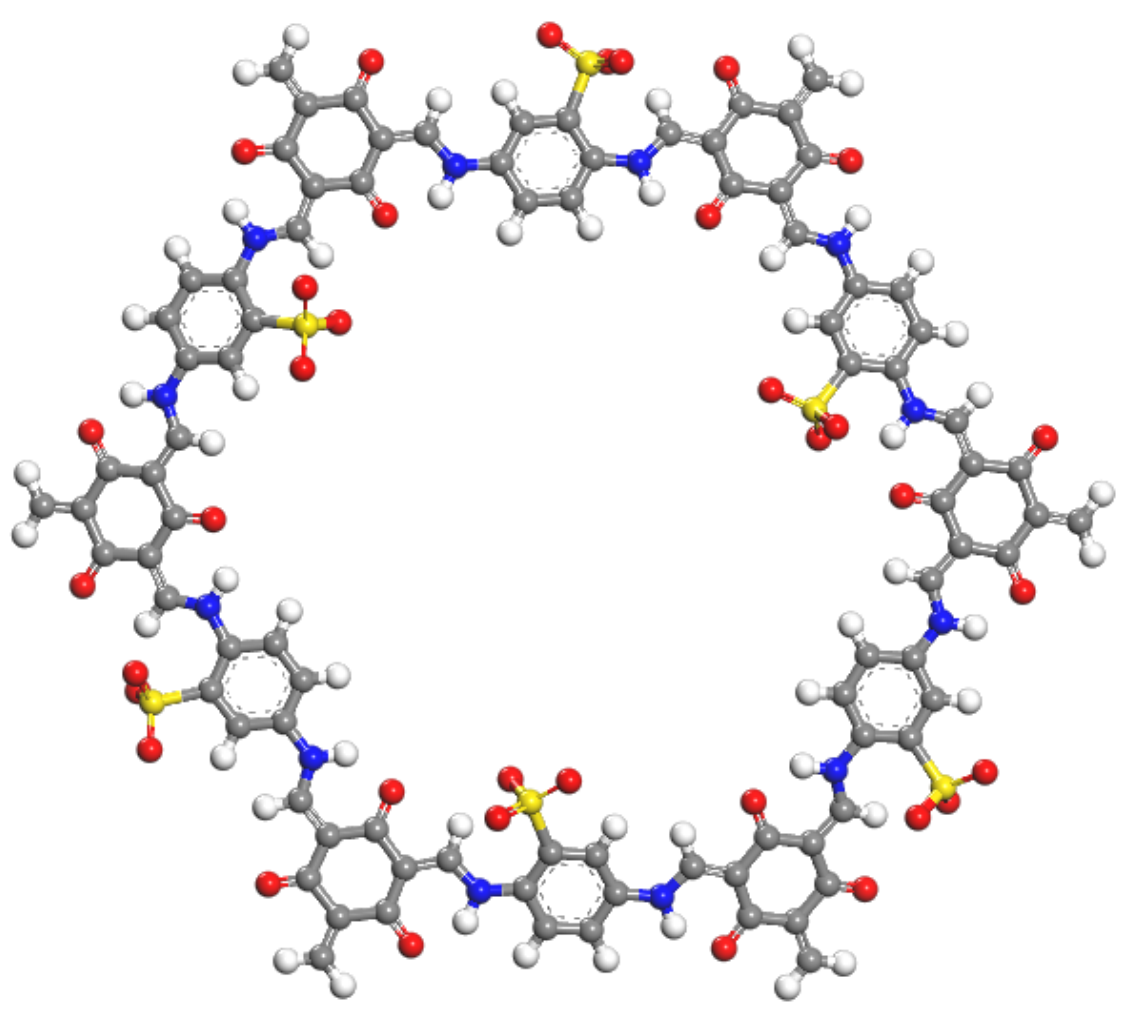

Fig. R3. The ball-and-stick model of TpPa-SO3-Na.

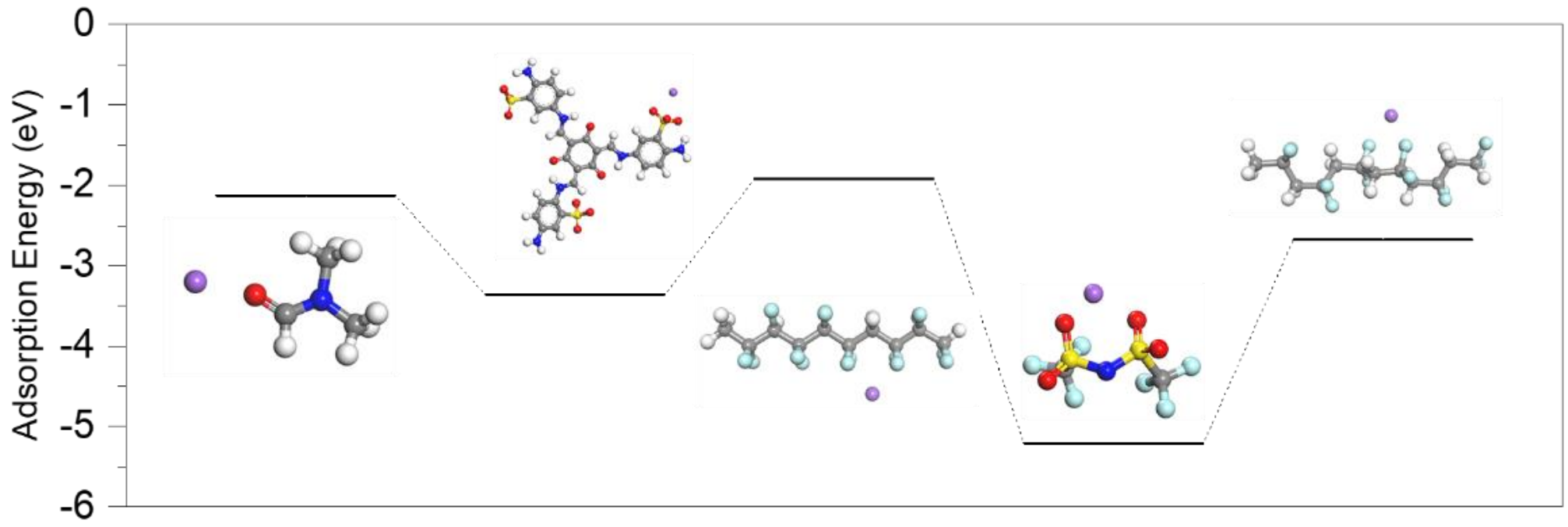

Fig. R4. Calculated adsorption energies of Na^+ with DMF, COF, $TFSI^-$, PTC, and PVDF.

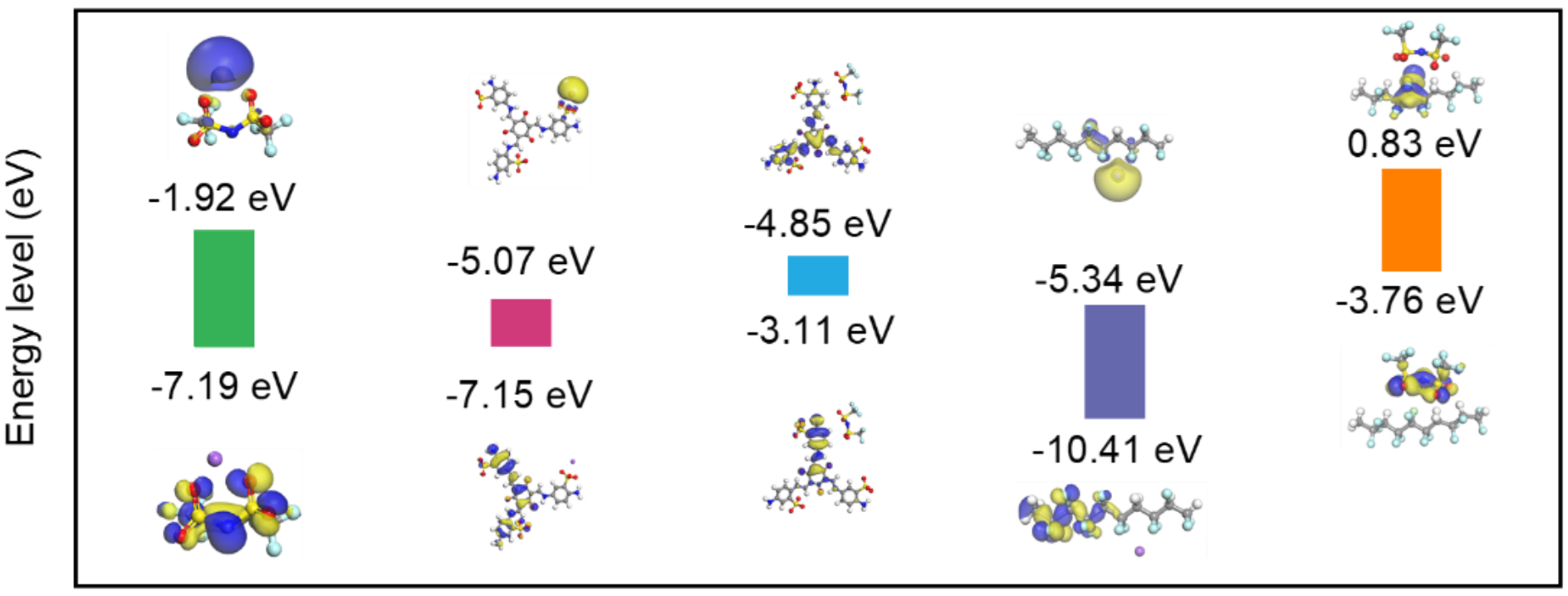

Fig. R5. The LUMO and HOMO energy values of Na^+-TFSI, Na^+-COF, $TFSI^-$-COF, Na^+-PTC, and TFSI-PTC.

2) In addition, the introduction would be strengthened by integrating recent and relevant literature on COF-based polymer electrolytes and DFT-driven battery materials design, specifically: (1) S. Kaviani, Energy Storage Materials (2025), DOI: 10.1016/j.ensm.2025.104671; (2) A. Shamsieva et al., Computational and Theoretical Chemistry (2025), DOI: 10.1016/j.comptc.2025.115445; and (3) S. Kaviani et al., Colloids and Surfaces A (2025), DOI: 10.1016/j.colsurfa.2024.135752.

[Author's Response]: Thank you for this helpful suggestion. We agree that the introduction would benefit from incorporating more recent work on COF-based polymer electrolytes and DFT-driven battery materials design. Accordingly, we have revised the Introduction section to better position our study in the context of (i) recent progress on COF-enabled solid polymer electrolytes and the role

of multi-scale computations (DFT/MD/ML) in rational electrolyte design, and (ii) recent DFT-guided design of porous organic framework-based battery materials (e.g., 2D COF-derived electrodes and heteroatom-doping strategies). Specifically, we have now cited and discussed the mentioned references in the revised Introduction. These additions strengthen the motivation and clearly connect our work with current advances and computationally guided design strategies. The mentioned works are cited and discussed as Ref. 29, Ref. 30, and Ref. 31 in the revised manuscript.

The corresponding revisions in the manuscript are as follows:

Lee et al. modified a separator with a sp^2 carbon-conjugated COF to induce a NaF-rich SEI and achieve a high ion transference number, effectively suppressing sodium dendrites and enabling long-term cycling of sodium metal batteries at high current densities.[24] Yousaf et al. developed a trifunctional halogenated COF-modified separator that anchors anions, lowers desolvation barriers, and induces a NaF-rich interface, enabling uniform Na^+ flux[25]. Sadegh Kaviani et al. reviewed the frontier of COF-based solid polymer electrolytes, highlighting the synergistic integration of DFT, MD simulations, and machine learning to provide a roadmap for the rational design of next-generation metal-ion batteries[26, 27, 28]. Building upon these insights, it is imperative to develop novel COF structures with tailored pore environments and functional sites to further optimize ion transport and interfacial stability in sodium-based batteries.

Reviewer #2 (Remarks to the Author)

Zeng et al. reported a covalent organic framework/poly(sodium acrylate-co-fluorinated ethylene) copolymer (COF/PNSE) composite electrolyte, which achieved high ionic conductivity and robust interfacial stability in solid-state sodium metal batteries through a synergistic "structure-chemistry" optimization strategy, while enabling fast-charging capability. The electrolyte exhibited a critical current density of 1.9 mA cm^{-2}, and the Na||NFM full cell delivered a discharge capacity of 82.5 mAh g^{-1} at 10 C, with 77.2% capacity retention after 1,000 cycles at 1 C and 83.5% retention after 2,000 cycles at 10 C. Extensive interfacial characterization revealed the interfacial chemical phenomena at the interfaces between the electrolyte, sodium metal anode, and NFM cathode during cycling. The study presents rich data with strong logical coherence, and the electrolyte design and characterization robustly support the main findings. I recommend the manuscript for minor revisions prior to acceptance and publication in Nature Communications. Specific comments are as follows:

[Author's Response]: We sincerely appreciate the time and effort the reviewer has dedicated to evaluating our manuscript, as well as the constructive feedback provided. Your insightful comments and suggestions have significantly contributed to enhancing the quality and clarity of our work. We have carefully addressed each of the issues raised and made the necessary revisions to the manuscript.

The detailed point-to-point responses are as follows:

1) The text size in some figures (e.g., Figure 1a, 1f, 2n, 3b, 3c, 3d, 4b) is too small and should be enlarged. Specific conductivity values should be labeled in Figure 2d. Clarify which elements the atoms in Figures 2i and 2j represent.

[Author's Response]: We sincerely appreciate the reviewer's constructive feedback regarding the figures and have implemented all the suggested improvements. Specifically, we have enlarged the font sizes in Figures 1a, 1f, 2n, 3b, 3c, 3d, and 4b to ensure clear legibility, labeled the specific conductivity

values directly in Figure 2d for better clarity, and updated Figures 2i and 2j with a color-coded legend and detailed caption to explicitly identify the elements represented by the atoms. These revisions have been incorporated into the revised manuscript to enhance the overall presentation of our data.

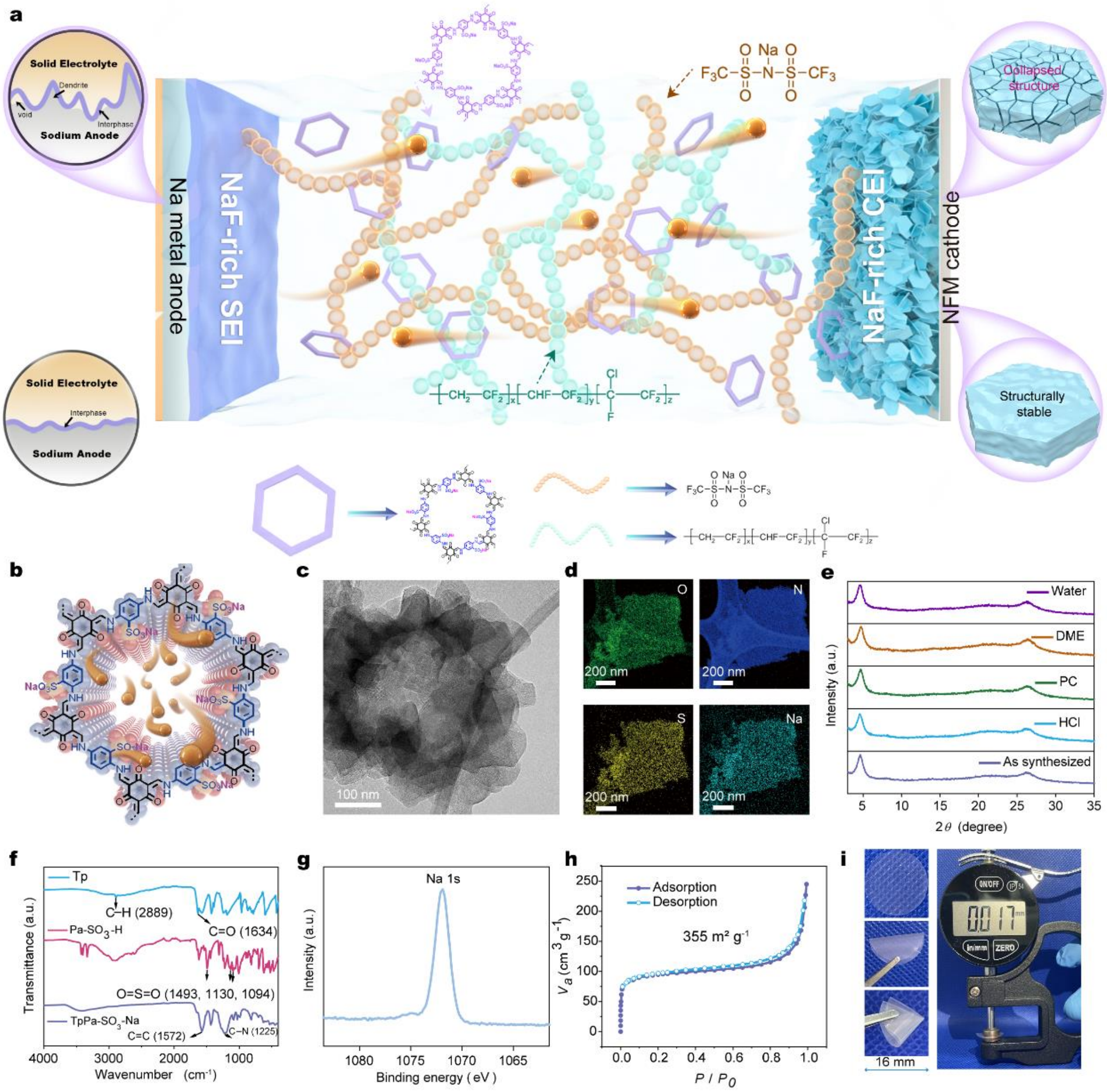

Figure 1. (a) Schematic illustration of the COF/PNSE composite electrolyte film in Na||NFM solid-state batteries; (b) Structure of TpPa-SO_3-Na material; (c) TEM image of TpPa-SO_3-Na material; (d) the corresponding elemental mapping images; (e) XRD pattern of the TpPa-SO_3-Na; (f) FT-IR spectrum; (g) XPS spectra of Na element; (h) Nitrogen adsorption-desorption isotherms curve; (i) Optical images showing mechanical flexibility and thickness measurements of the COF/PNSE composite electrolyte film.

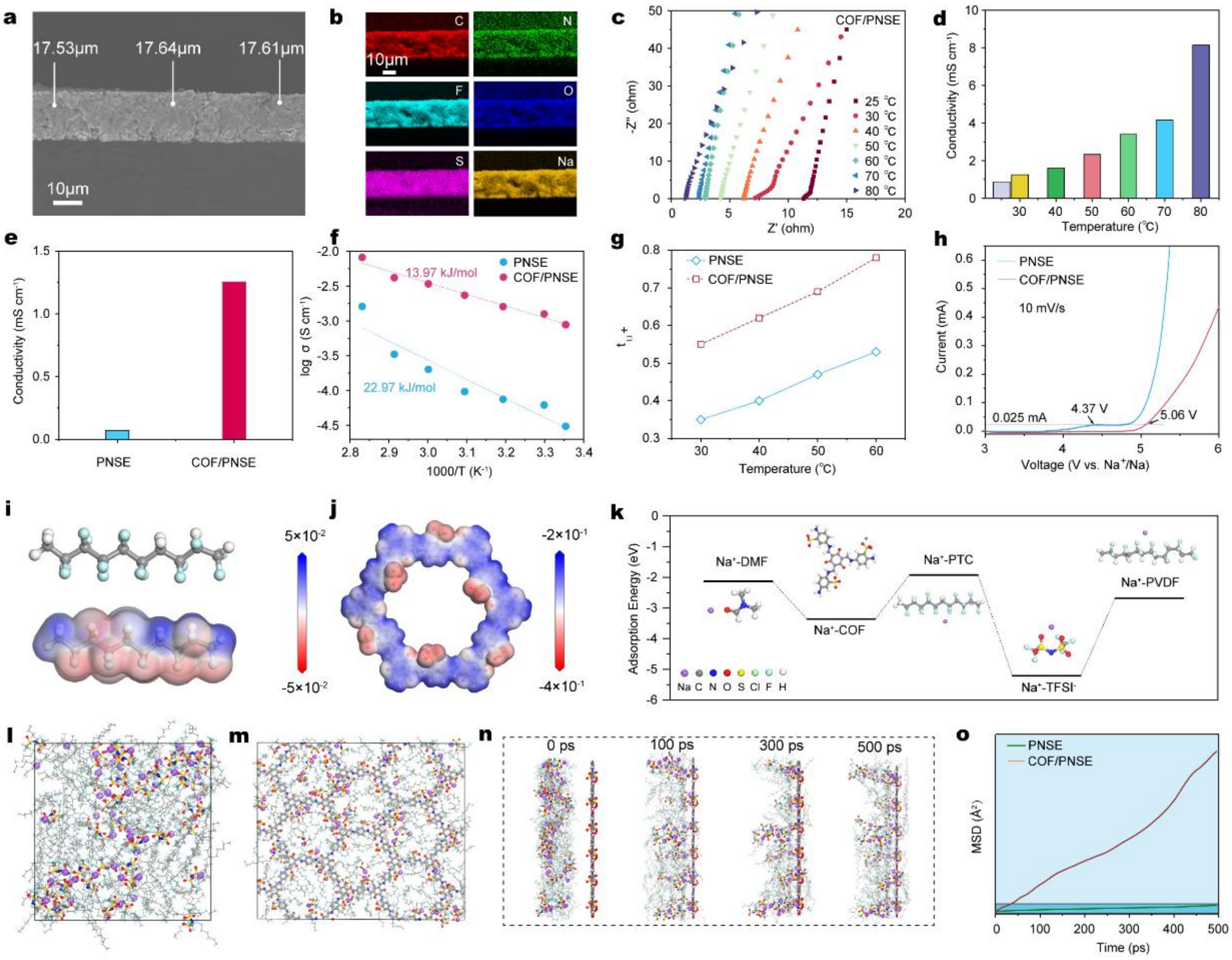

Figure 2. (a) SEM image of the COF/PNSE. (b) Corresponding elemental mapping images. (c) EIS Nyquist plots of COF/PNSE at varying temperatures. (d) Temperature-dependent ionic conductivity of COF/PNSE. (e) Room-temperature ionic conductivity comparison between PNSE and COF/PNSE. (f) Activation energy (E_a) for Na^+ migration in PNSE and COF/PNSE. (g) Temperature-dependent Na^+ transference numbers for PNSE and COF/PNSE. (h) LSV curves of PNSE and COF/PNSE. (i) Molecular structure and electrostatic potential distribution of PVDF-TrFE-CFE copolymer. (j) Electrostatic potential distribution of the COF framework. (k) Calculated adsorption energies of Na^+ with DMF, COF, $TFSI^-$, PTC, and PVDF. (l) MD simulation snapshots of the PNSE system. (m) MD simulation snapshots of the COF/PNSE composite system. (n) Representative simulation snapshots of Li^+ migration pathways in the COF/PNSE system at room temperature. (o) Mean squared displacement (MSD) of Li^+ ions in COF/PNSE and PNSE systems derived from MD simulations.

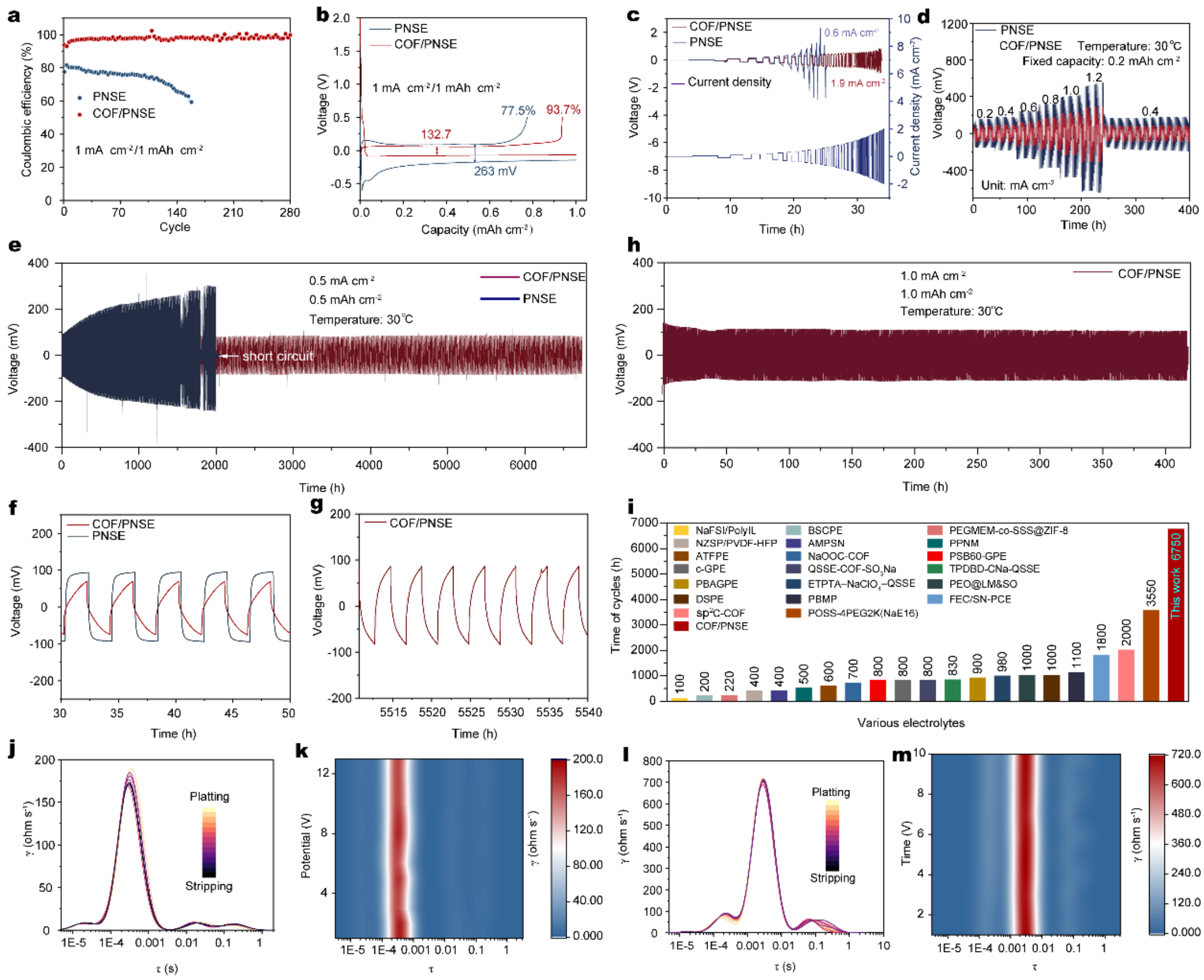

Figure 3. (a) Cycling stability of Na||Cu half-cells evaluated at 1.0 mA cm^{-2}/1.0 mAh cm^{-2}. (b) Initial Na deposition/stripping voltage profiles for Na||Cu cells employing PNSE and COF/PNSE electrolytes. (c) Critical current density analysis for Na||Na solid-state cells utilizing PNSE and COF/PNSE electrolytes. (d) Rate performance of PNSE-based and COF/PNSE-based cells. (e) Long-term electrochemical stability of Na||Na symmetric cells cycled at 0.5 mA cm^{-2}/0.5 mAh cm^{-2} with PNSE and COF/PNSE. (f, g) Enlarged voltage-time traces of Na plating/stripping cycles from (e). (h) High-rate cycling capability of Na||Na symmetric cells at 1 mA cm^{-2}/1 mAh cm^{-2} using COF/PNSE. (i) Cycling lifespan benchmarking against representative sodium solid-state battery electrolytes reported in recent literature. (j, k) Distribution of Relaxation Times (DRT) analysis for Na plating and stripping kinetics in COF/PNSE-containing symmetric cells. (l, m) Corresponding DRT spectra for PNSE-only symmetric cells.

2) Enrich and refine the review of PVDF-based sodium-ion electrolyte advancements in the background section.

[Author's Response]: We sincerely thank the reviewer for this constructive suggestion. We agree that a more comprehensive background on PVDF-based electrolytes provides better context for our work and strengthens the manuscript. Accordingly, we have enriched and refined the introduction section. These revisions have been highlighted in red color in the revised manuscript.

The corresponding revisions in the manuscript are as follows:

Polymer electrolytes, particularly poly(ethylene oxide) (PEO) derivatives, have been extensively investigated for SSSBs due to their flexibility, processability, and favorable interfacial compatibility[10, 11].Nevertheless, their room-temperature ionic conductivity seldom exceeds 10^{-4} S cm^{-1}, constrained by high crystallinity and limited segmental mobility[12]. More critically, the narrow electrochemical stability window of PEO (<3.8 V) limits compatibility with high-voltage cathodes required for high-energy-density systems. Although poly(vinylidene fluoride) (PVDF)-based terpolymers offer improved anodic stability (>4.5 V), their chemically inert backbone exhibits negligible Na^+ solvation capability, resulting in low transference numbers (<0.3) and severe concentration polarization under high-rate cycling[13, 14]. To circumvent these kinetic bottlenecks, recent strategies have pivoted towards modulating the local coordination environment and hierarchical morphology within PVDF-based matrices. Specifically, the high electronegativity of fluorine atoms in the PVDF-HFP units can theoretically induce a strong dipole moment[15]; however, their poor coordinative interaction with Na^+ ions leads to a "decoupled" transport mechanism that is often hindered by the high degree of polymer crystallinity[16]. Recent breakthroughs have demonstrated that the introduction of specific filler-matrix interactions, such as Lewis acid-base coupling[17] or tethered anion sites[18], can effectively reconfigure the Na^+ hopping pathways, transitioning from slow segmental-dependent motion to fast, channel-mediated transport. These advancements underscore the necessity of a trifunctional design of combining high oxidative stability, accelerated desolvation kinetics, and uniform ion flux to realize truly viable PVDF-based solid-state sodium batteries.

3) Investigate the periodic black shadows in Figures 3d and 3h, verify their origin, and ensure image clarity.

[Author's Response]: We sincerely apologize for the oversight regarding the image quality in the original manuscript. We have carefully investigated the periodic black shadows in Figures 3d and 3h. We confirmed that these shadows are actually rendering artifacts caused by insufficient resolution and compression during the image export process from our plotting software. To resolve this and ensure full image clarity, we have re-exported the figures at a high resolution. The updated, clear images are provided below and have been updated in the revised manuscript.

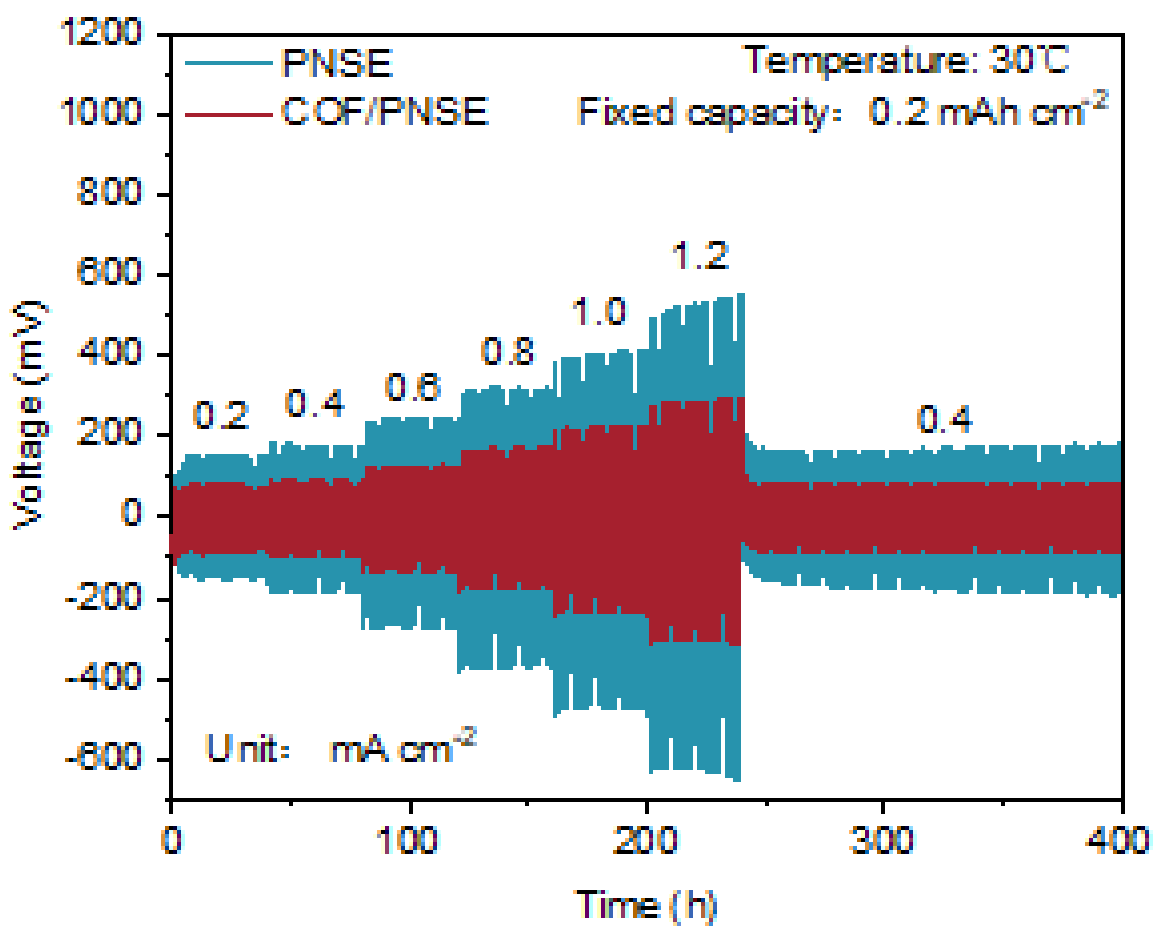

Fig. R6. Rate performance of PNSE-based and COF/PNSE-based cells.

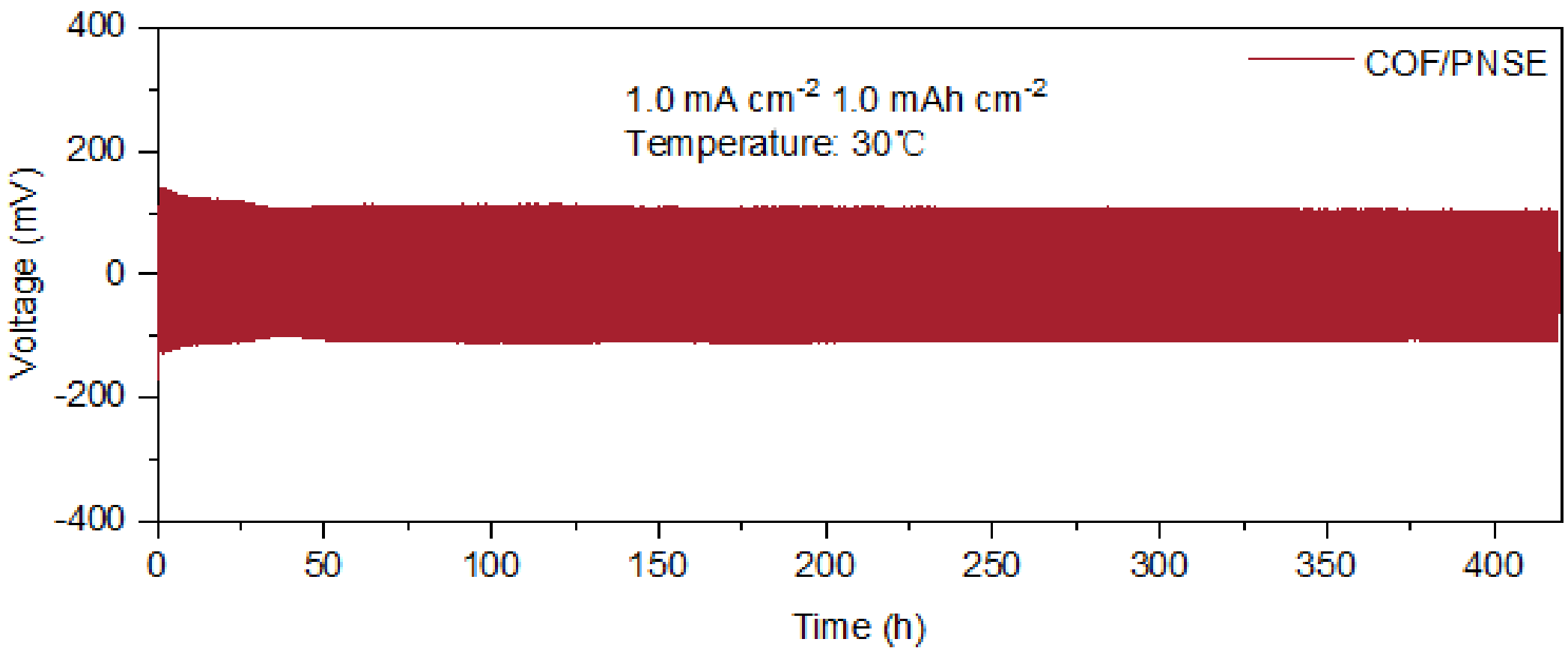

Fig. R7. High-rate cycling capability of Na||Na symmetric cells at 1 mA cm^{-2}/1

mAh cm^{-2} using COF/PNSE.

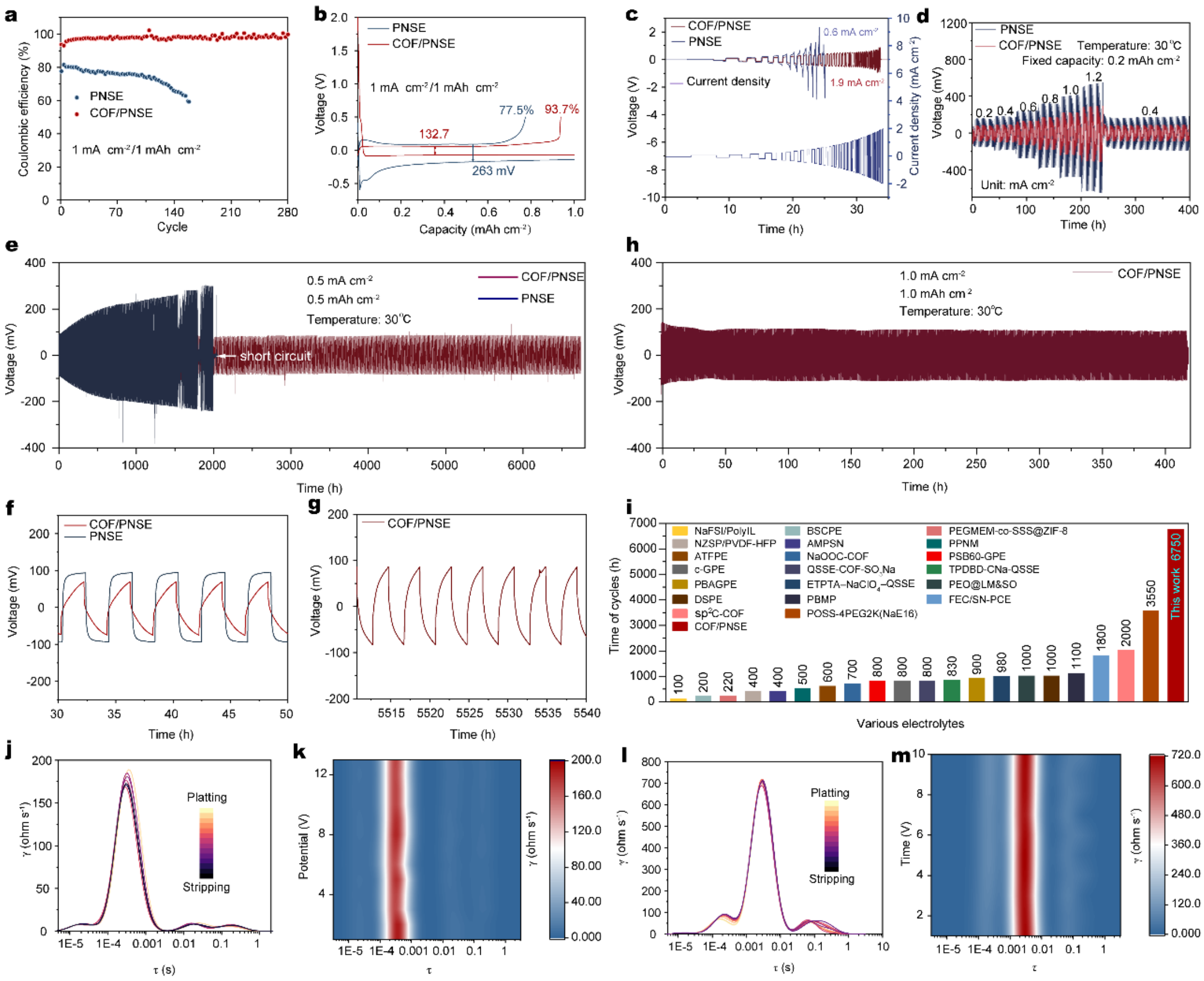

Figure 3. (a) Cycling stability of Na||Cu half-cells evaluated at 1.0 mA cm^{-2}/1.0 mAh cm^{-2}. (b) Initial Na deposition/stripping voltage profiles for Na||Cu cells employing PNSE and COF/PNSE electrolytes. (c) Critical current density analysis for Na||Na solid-state cells utilizing PNSE and COF/PNSE electrolytes. (d) Rate performance of PNSE-based and COF/PNSE-based cells. (e) Long-term electrochemical stability of Na||Na symmetric cells cycled at 0.5 mA cm^{-2}/0.5 mAh cm^{-2} with PNSE and COF/PNSE. (f, g) Enlarged voltage-time traces of Na plating/stripping cycles from (e). (h) High-rate cycling capability of Na||Na symmetric cells at 1 mA cm^{-2}/1 mAh cm^{-2} using COF/PNSE. (i) Cycling lifespan benchmarking against representative sodium solid-state battery electrolytes reported in recent literature. (j, k) Distribution of Relaxation Times (DRT) analysis for Na plating and stripping kinetics in COF/PNSE-containing symmetric cells. (l, m) Corresponding DRT spectra for PNSE-only symmetric cells.

4) Strengthen the analysis of in-situ EIS and DRT (Distribution of Relaxation

Times) data, explicitly indicating resistance value changes.

[Author's Response]: We sincerely thank the reviewer for this constructive suggestion. We fully agree that a deeper, quantitative analysis of the in-situ EIS and DRT data is essential for elucidating the interfacial evolution and kinetic behaviors.

In accordance with your advice, we have significantly strengthened the discussion and explicitly indicated the resistance value changes in the revised manuscript. Specifically, we have detailed the analysis in two key parts: For the symmetric cells (**Figures 3j-3m**): We have thoroughly analyzed the in-situ EIS and corresponding DRT spectra, explicitly discussing the evolution of the specific interfacial resistance values during the plating/stripping processes.

For the Na||SE||NFM full cells (**Figures 5f-5i**): We have carefully investigated the in-situ EIS and DRT results during the charge/discharge cycles. The quantitative changes in the bulk resistance, charge transfer resistance, and solid electrolyte interphase (SEI) resistance have been explicitly pointed out and discussed in depth.

The corresponding revisions in the manuscript are as follows:

By leveraging in situ electrochemical impedance spectroscopy (EIS) coupled with distribution of relaxation times (DRT) deconvolution (Figs. 3j–m), we gained fundamental insights into the interfacial kinetics. During continuous Na deposition at 0.2 mA cm^{-2}, the COF/PNSE configuration exhibited a spontaneous decline in interfacial resistance from 375 Ω to 334 Ω (Fig. S28b), a trend that stands in stark contrast to the resistive growth (from 1,410 Ω to 1,460 Ω) observed in the pristine PNSE (Fig. S28a). The DRT spectra effectively resolved the multifaceted electrochemical response into discrete timescales; specifically, the relaxation peaks identified at 10^1–10^3 Hz and below 10^0 Hz were attributed to the charge-transfer (R_{ct}) and ion diffusion (R_{diff}) processes, respectively. Our analysis reveals that COF incorporation significantly suppresses the R_{ct} intensity, providing tangible evidence of a lower desolvation energy barrier and accelerated interfacial Na^+ exchange kinetics.

In a sharp departure from this behavior, PNSE alone suffers from broadened and intensified relaxation peaks, indicative of slow interfacial kinetics and the stochastic buildup of resistive species. Furthermore, the remarkable temporal stability of the DRT signatures for COF/PNSE over cycling underscores the formation of a highly uniform and robust SEI. These results establish COF/PNSE as a paradigm-shifting electrolyte design, effectively harmonizing mechanical reinforcement with ultrafast Na^+ transport to circumvent the long-standing bottlenecks in high-energy-density solid-state sodium batteries.

Operando DRT deconvolution (**Figs. 5f–i**) effectively resolves the kinetic disparities between the two systems, identifying characteristic responses for bulk Na^+ diffusion (t ~0.1–1 s) and interfacial charge-transfer. In the COF/PNSE configuration, the marked suppression of low-frequency impedance signals a radical acceleration in diffusion kinetics through the electrolyte framework (**Fig. S39b**). In stark contrast, the PNSE-only cells exhibit amplified peak intensities and volatile frequency migrations (**Figs. 5h–i, S39a**), symptomatic of sluggish charge-transfer across a highly resistive and structurally unstable CEI at the cathode interface.

5) Supplement cryo-electron microscopy sample preparation methods for anode/cathode in the Supporting Information (SI) file, including detailed parameters.

[Author's Response]: We sincerely thank the reviewer for this valuable suggestion. We have fully supplemented the cryo-EM sample preparation methods and testing details for both the Na metal anode and the NFM cathode in the revised Supporting Information.

(1) Cryo-EM sample preparation and testing details for sodium metal anodes

To preserve the intrinsic morphology of the highly reactive deposited sodium and its fragile solid electrolyte interphase (SEI) film at the nanoscale in situ, a strict cold-chain transfer and ultra-low dose cryo-electron microscopy

(Cryo-EM) technique were employed in this study. First, solid-state Na||Cu cells were assembled with 2~3 bare copper grids placed separately on the Cu foil. The cells were then subjected to a Na||Cu plating/stripping process. This study focuses on the initial plating behavior, conducted at a current density of 0.5 mA cm^{-2} with a fixed plating capacity of 0.5 mAh cm^{-2}. After deposition, the cells were transferred to an argon-filled glovebox with strictly controlled water and oxygen contents (< 0.01 ppm). The cycled cells were carefully disassembled, and the copper grids with deposited Na metal were retrieved. Subsequently, the samples were fixed and rapidly plunged into liquid nitrogen (plunge freezing). The ultra-low temperature effectively suppressed the thermal vibration of molecules, significantly enhancing the tolerance limit of the electron-beam-sensitive materials. The Cryo-EM characterizations were performed on a Titan Krios G3i (D3845) microscope. The frozen samples were safely transferred into the microscope chamber under completely air-isolated, closed-loop conditions using a high-vacuum cryo-transfer system. Throughout the imaging process, the sample temperature was strictly maintained below -70 °C. To acquire non-destructive surface structures, the final morphological observations were conducted using an ultra-low dose electron beam with an adjustable accelerating voltage of 80–300 kV. The electron dose rate was set to 12 $e^-/Å^2 \cdot s$ with an exposure time of 10 s (resulting in a total electron dose of 120 $e^-/Å^2$). This rigorous setup minimized electron beam-induced damage to the delicate structures, ensuring the acquisition of high-fidelity and high-resolution data.

(2) Cryo-EM sample preparation and testing details for NNM cathodes

To accurately elucidate the authentic surface evolution of the cycled NFM cathode, its sample preparation and testing also strictly followed the aforementioned cryo-protection procedures. After the cycled cells were disassembled in the glovebox, the active materials were carefully scraped from the retrieved NFM cathode and uniformly dispersed onto a carbon-coated copper grid. Utilizing the high-vacuum cryo-transfer system, the copper grid loaded with the cathode sample was rapidly plunged into liquid nitrogen without

any damage or air exposure. It was then loaded into a cryo-sample box and transferred into the Cryo-EM chamber. During the characterization, not only was liquid nitrogen continuously injected to maintain the ultra-low temperature environment, but a low-dose imaging mode was also employed to further acquire the genuine morphology and compositional information of the CEI layer on the cathode surface.

6) Annotate etched XPS data (e.g., Figure 4e) with specific corresponding species/fragments. Describe electrode materials, processing details, and assembly protocols for Figures 5j-5l in the SI.

[Author's Response]: We sincerely thank the reviewer for these constructive suggestions, which significantly enhance the clarity of our data and the reproducibility of our experimental results. We have fully addressed both requests in the revised manuscript and Supporting Information.

(1) As suggested, we have carefully updated the etched XPS spectra in the revised manuscript (specifically Figures 4e, 4f, 4g, and 4h). We have explicitly annotated the fitted peaks with their corresponding chemical species and functional groups directly on the plots, providing a much clearer and more intuitive illustration of the interphase composition.

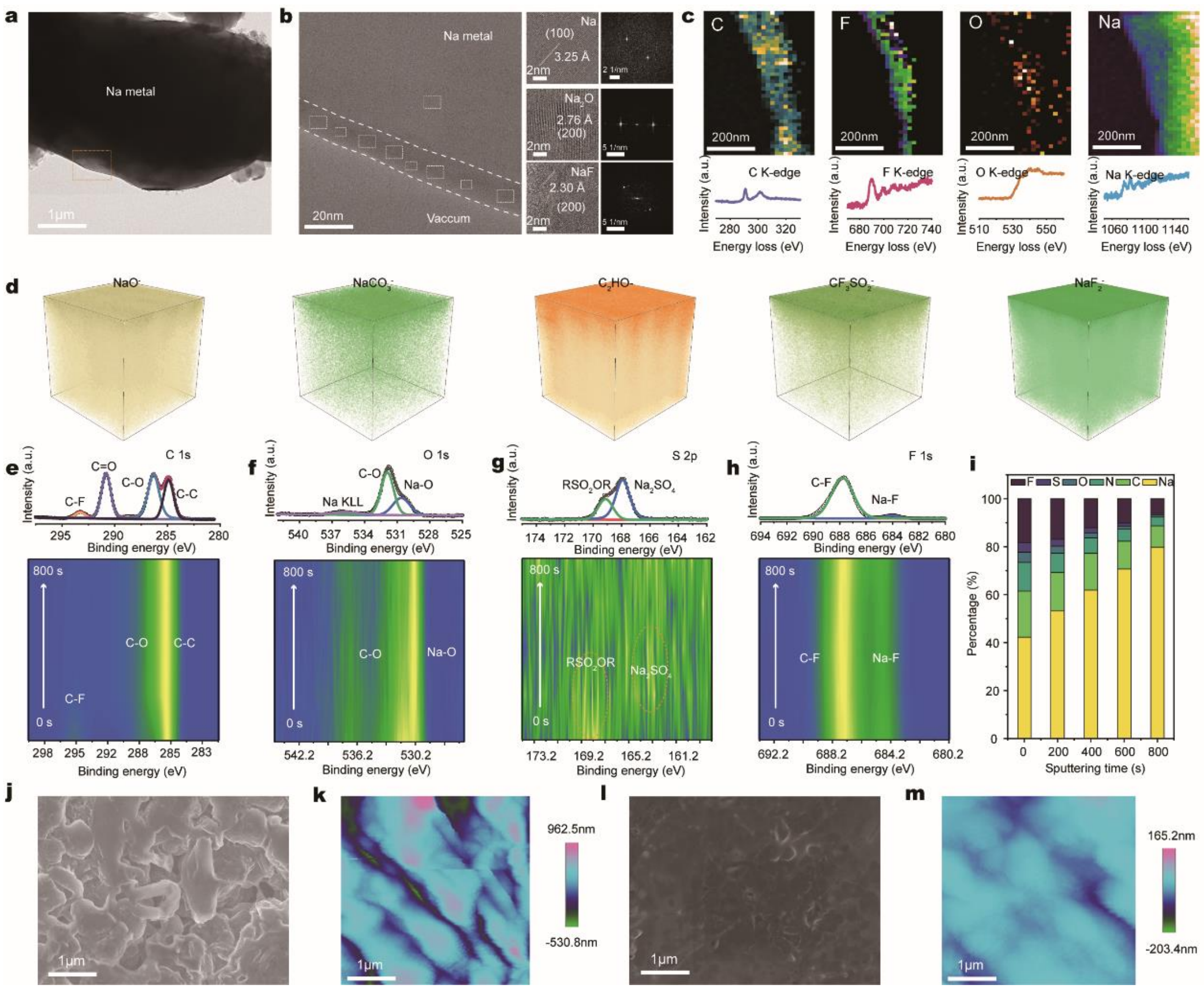

Fig. R8. (a) Low-magnification cryo-TEM image of deposited Na metal. (b) HRTEM images and corresponding SAED patterns of the SEI formed with COF/PNSE electrolyte. (c) Elemental mapping images and EELS line scans across the C, F, O, and Na K-edges. (d) TOF-SIMS depth profiles of NaO^-, $NaCO_3^-$, C_2HO^-, $CF_3SO_3^-$, and NaF_2^- fragments at the Na metal surface after cycling with COF/PNSE. (e–i) Depth-profile XPS analysis of cycled Na metal: (e) C 1s, (f) O 1s, (g) S 2p, (h) F 1s spectra, and (i) atomic concentration ratios of key elements at various etching durations. (j) SEM image and (j) corresponding topography via AFM for Na metal cycled with PNSE. (l,m) SEM and AFM images for Na metal cycled with COF/PNSE electrolyte.

(2) We have added a comprehensive description of the electrode materials, processing details, and precise cell assembly protocols corresponding to the electrochemical tests shown in Figures 5j-5l. These specific experimental details have been newly incorporated into the revised Supporting Information.

The revisions are as follows:

First, the mass ratios of the active material, conductive additive, and binder for the NFM cathode and HC anode were set to 90:5:5 and 92:3:5, respectively. To prevent sodium plating during charging, the N/P ratio of the full cell was strictly controlled at 1.15, corresponding to areal mass loadings of approximately 15 mg/cm² for the cathode and 6.5 mg/cm² for the anode. Second, to minimize the interfacial contact resistance, this work eliminated the use of traditional freestanding separators. Instead, the PVDF-based polymer electrolyte (COF/PNSE) was directly cast onto the electrode surfaces to form an integrated electrolyte/electrode structure *in situ*. During the pouch cell assembly stage, to prevent sodium plating induced by edge effects, the dimensions of the cut anode (52×72 mm) were designed to be slightly larger than those of the cathode (50×70 mm). Subsequently, a multi-layer stacking process was employed to alternately stack the integrated electrodes. The cathode and anode current collectors were then ultrasonically welded to aluminum and nickel tabs, respectively, and the entire assembly was enclosed within an aluminum-laminated film (ALF) for vacuum sealing. Following the sealing process, to further enhance the interfacial contact between the polymer electrolyte and the electrode materials, the assembled cells were first subjected to two initial cycles at 60 °C for activation. Subsequent electrochemical performance tests were conducted at 25 °C within a voltage range of 2~4 V, applying a testing protocol of 0.5 C for charging and 1 C for discharging.

7) Analyze depth-dependent elemental (C, F, O, etc.) distribution at the cathode interface using XPS and TOF-SIMS (Time-of-Flight Secondary Ion Mass Spectrometry) for cross-validation.

[Author's Response]: We sincerely thank the reviewer for this insightful and highly constructive suggestion. We fully agree that cross-validating the interphase composition using both depth-profiling XPS and TOF-SIMS provides a much more comprehensive and convincing understanding of the cathode-

electrolyte interphase. The underlying mechanistic nuances are scrutinized as follows:

To further decode the chemical architecture of the cathode electrolyte interphase (CEI), XPS depth-profiling (Fig. R9) was executed, revealing a well-defined gradient distribution of C, F, and O species as a function of etching depth. At the topmost interface, the C 1s and O 1s signatures are predominantly governed by organic moieties originating from electrolyte decomposition. Upon deeper penetration into the interphase, these organic-related signals progressively diminish, a transition accompanied by the relative intensification of inorganic constituents. Notably, the F 1s spectra (Fig. R9b) reveal that inorganic fluoride species, specifically NaF-based fragments, persist even within the deeper sub-layers, providing tangible evidence for the formation of a dense, F-rich protective shield. This spatial evolution—characterized by a transition from an organic-rich outer skin to an inorganic-dominated inner core—underscores the hierarchical robustness of the CEI in the COF/PNSE system. Such an architecture effectively isolates the high-voltage cathode from parasitic side reactions while simultaneously maintaining unhindered channels for ion transport. The spatial distribution of C^-, O^-, and F^- species was further visualized through 2D TOF-SIMS mapping (Fig. R10), which confirms a spatially uniform and continuous enrichment of these characteristic fragments across the cathode/electrolyte interface.

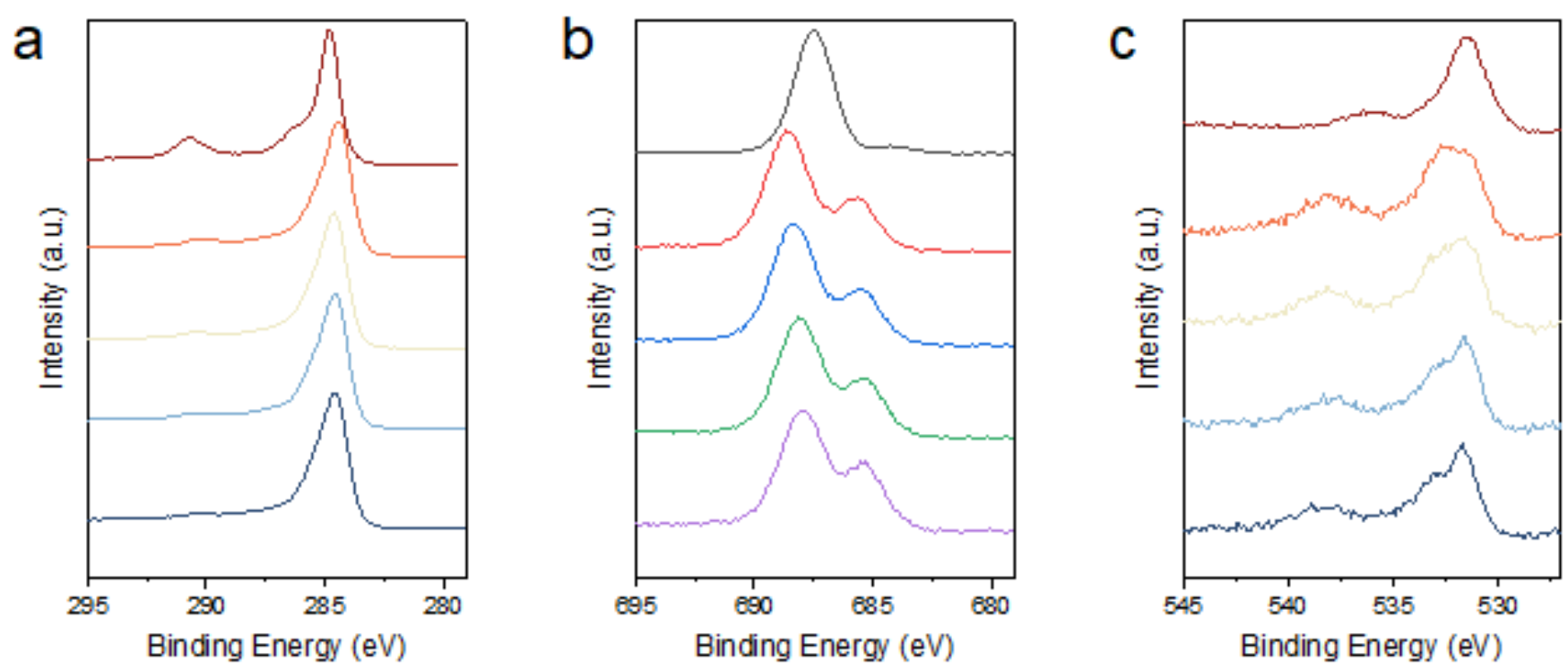

Fig. R9. XPS depth-dependent elemental distribution of (a) C, (b) F, and (c) O at the cathode interface.

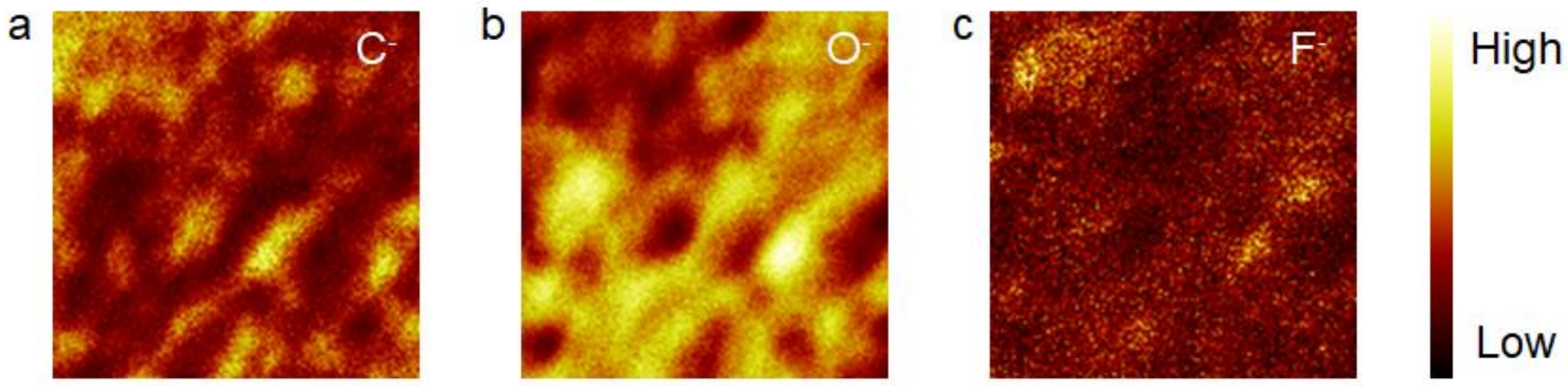

Fig. R10. 2D TOF-SIMS images of (a) C^-, (b) O^- , and (d) F^- at the cathode/electrolyte interface.

Reviewer #3 (Remarks to the Author)

This manuscript reports a COF-enhanced polymer electrolyte for fast-charging sodium solid-state batteries, claiming simultaneously high ionic conductivity, ultralong cycling stability, excellent interfacial robustness, and scalability to Ah-level pouch cells. While the topic is of interest, the current manuscript raises serious concerns regarding credibility, mechanistic rigor, and scientific positioning.

[Author's Response]: We sincerely appreciate the reviewer's rigorous evaluation, which has guided us to refine the scientific depth of our work. To systematically address the concerns regarding credibility and mechanistic rigor, we have restructured our response around three pivotal pillars that define the innovation and validity of this study:

(1) We pioneered a **COF-integrated PVDF-based terpolymer** (PVDF-terpolymer) electrolyte system. The synergistic interplay between the ordered COF channels and the high-dielectric terpolymer matrix optimizes the Na^+ transport landscape, achieving exceptional ionic conductivity and enabling stable operation at an **ultra-high rate of 10C**. This molecular-level structural design is the foundation of the **fast-charging capability** reported.

(2) To dismantle doubts regarding interfacial stability, we employed **Cryo-TEM to resolve the SEI/CEI architectures** at their native states. By effectively suppressing beam-induced damage, we provided unambiguous, high-resolution evidence of the interfacial components, confirming the formation of a dense and robust protective layer.

(3) A robust evidentiary chain was established through a suite of **operando characterizations**. Specifically, **operando FT-IR** captured the dynamic evolution of the solvation shell; **operando DRT** deconvoluted the kinetic relaxation of charge-transfer processes; and **XPS depth-profiling (complemented by TOF-SIMS mapping)** elucidated the 3D chemical gradient of the interphase.

By integrating these multi-scale insights—from macromolecular design to

atomic imaging and real-time kinetic tracking—we provide a “mechanistic rigor” that bridges the gap between material innovation and the high-performance metrics of Ah-level pouch cells. We believe these clarifications and new datasets significantly elevate the scientific positioning and credibility of our manuscript.

The detailed point-to-point responses are as follows:

1) First, the performance metrics reported are exceptionally high for a polymer-based sodium solid-state electrolyte (e.g., ~1.2 mS cm^{-1} at 25 °C, >6,700 h Na||Na cycling, stable operation at 10 C for thousands of cycles, and Ah-level pouch cells). These results, taken together, are far beyond the current state of the art, yet they are supported by limited statistical validation. Key data lack reproducibility analysis, error bars, and multi-cell verification, making the robustness of the conclusions questionable.

[Author’s Response]: We fully concur with the reviewer that rigorous statistical validation is the cornerstone of credible electrochemical research, especially for high-performance electrolyte systems. We have addressed these concerns through comprehensive re-testing and systematic data enrichment:

(1) To fortify the veracity of the reported 1.2 mS cm^{-1} conductivity, we performed rigorous benchmarking across five independent batches of COF-PVDF terpolymer membranes (Fig. R1, R2 and Table R1). The updated Fig. 2 now incorporates error bars reflecting the standard deviation, ensuring the empirical reproducibility of our findings. This high-performance transport originates from a designed “high-dielectric/ordered-channel” strategy: while the high-dielectric PVDF-terpolymer matrix promotes effective sodium salt dissociation (corroborated by our XPS/FT-IR spectroscopic evidence), the vertically-aligned COF frameworks facilitate low-tortuosity conduits for rapid, long-range cation hopping.

Our data is fundamentally consistent with the upper echelons of current solid-state electrolyte research. As summarized, competitive conductivities

have been documented in recent high-impact works: for instance, Chen et al. reported a PVDF-based electrolyte reaching 1.37 mS cm^{-1} (*Angewandte Chemie International Edition, 2023, 62(52): e202315076.*); Passerini's group achieved 1.43 mS cm^{-1} in a PEO-based system (*Energy & Environmental Science, 2022, 15(6): 2610-2618.*); Cui et al. demonstrated an in situ formed PCIE with 2.3 mS cm^{-1} at 30 °C (*Nature Communications, 2025, 16(1): 2979.*); and Bresser's team developed a multi-block SIPE with a conductivity of approximately 2.6 mS cm^{-1} (*Angewandte Chemie, 2023, 135(43): e202308699.*). These benchmarks collectively underscore that our conductivity metrics, while exceptional, are physically grounded and well-aligned with the state-of-the-art developments in polymer-based sodium conductors.

(2) Regarding the longevity exceeding 6700 h, we have provided a comprehensive benchmarking analysis against state-of-the-art literature in Table R2. While this duration is indeed exceptional, it is physically consistent with the moderate current density (0.5 mA cm^{-2}) employed in our symmetric cell tests. At this regime, the piezoelectric-reinforced framework effectively buffers the localized interfacial strain and suppresses dendrite initiation without triggering severe concentration polarization. The feasibility of such performance is well-supported by recent breakthroughs in the field. For instance, Guo et al. recently unveiled a high-entropy solid-state electrolyte reaching a room-temperature conductivity of 1.3 mS cm^{-1}, which sustains stable Na plating/stripping for over 10,000 hours at 0.1 mA cm^{-2} (*Energy Environ. Sci., 2026, 19, 860*). Similarly, Zheng et al. documented a hybrid network SPE that maintains stability for over 5,150 h and 3,350 h at 0.1 and 0.5 mA cm^{-2}, respectively (*Adv. Energy Mater., 2018, 8, 1801885*). Detailed performance metrics and multi-cell statistical data are summarized in the following table.

(3) The reviewer's concern regarding the "exceptionally high" metrics is noted; however, we emphasize that these results are the logical manifestation of both electrolyte innovation and electrode optimization. The 10C fast-charging capability is rooted in two synergistically optimized factors:

First, our COF-PVDF terpolymer electrolyte provides superior cation kinetics and interfacial stability, as rigorously validated by Operando DRT (minimizing charge-transfer resistance) and Cryo-TEM (resolving a robust, native-state CEI).

Second, the performance is intrinsically tied to our customized P2-NNM cathode. We have now included detailed structural and electrochemical characterization of this material in the revised Supporting Information. It is crucial to highlight that the P2-NNM framework possesses inherently fast Na^+ diffusion kinetics; as demonstrated in our previous report (*ACS Nano, 19 (35)(2025), 31395-31406.*), this cathode can sustain an ultra-high rate of 200C in liquid electrolytes. Consequently, achieving 10C in an optimized solid-state batteries is not only plausible but a reasonable translation of the material's high-rate under solid-state constraints.

Furthermore, a comprehensive literature survey (summarized in Table R3) reveals that superior rate capability and longevity are emerging as inherent advantages of sodium-based solid-state chemistries compared to lithium counterparts, owing to the lower desolvation energy and faster interfacial kinetics of Na^+. Detailed comparisons with other state-of-the-art SSMBs are provided in the table below.

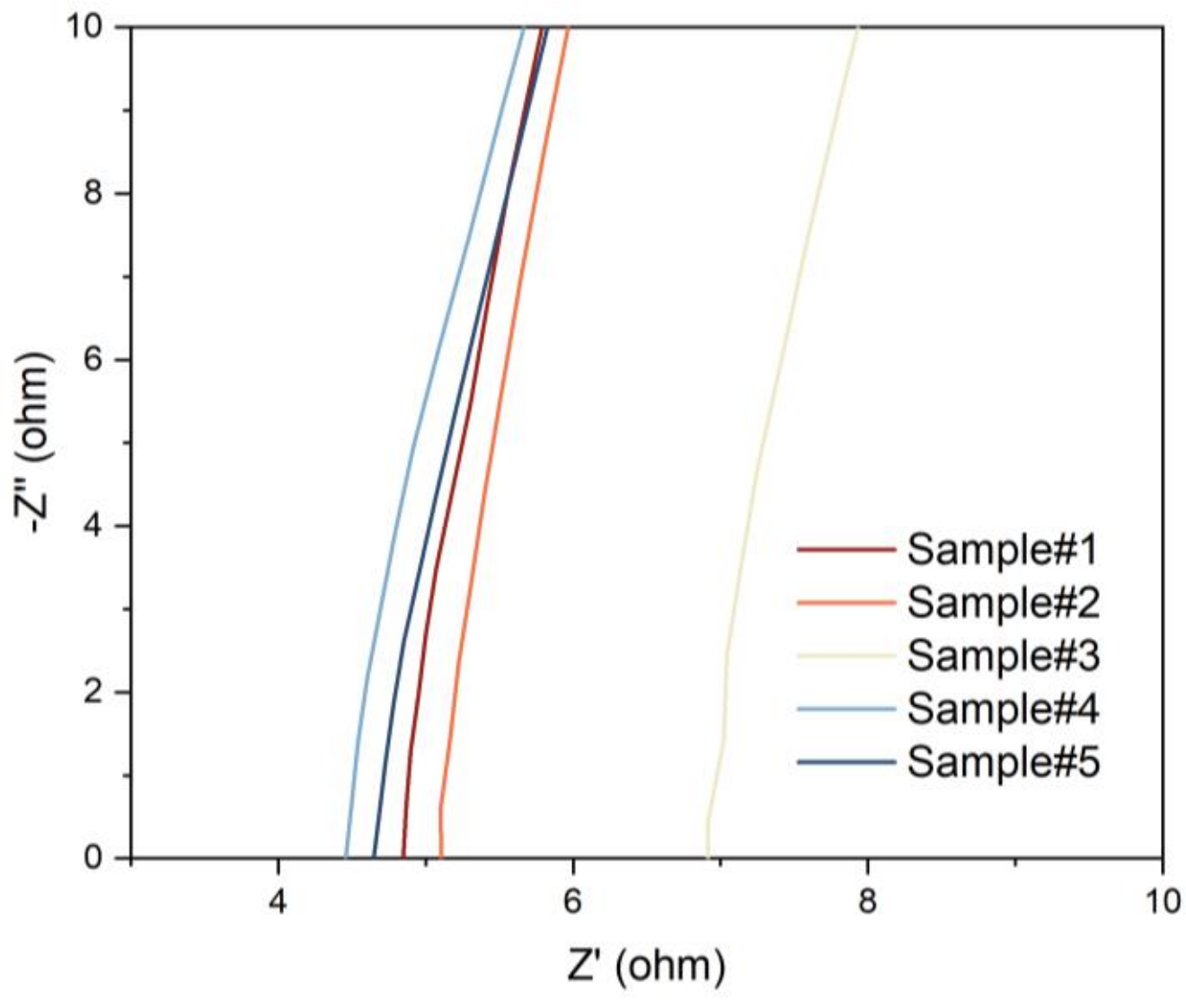

Fig. R1. EIS curves of five COF/PNSE film using SS|| COF/PNSE||SS test.

Table R1. The ion conductivity of COF-PVDF terpolymer membranes performed rigorous benchmarking across five independent batches.

Samples	T(℃)	R (Ω)	σ (mS/cm)
COF/PNSE-1	30	4.848	1.540
COF/PNSE-2	30	5.108	1.462
COF/PNSE-3	30	6.915	1.080
COF/PNSE-4	30	4.454	1.676
COF/PNSE-5	30	4.629	1.613

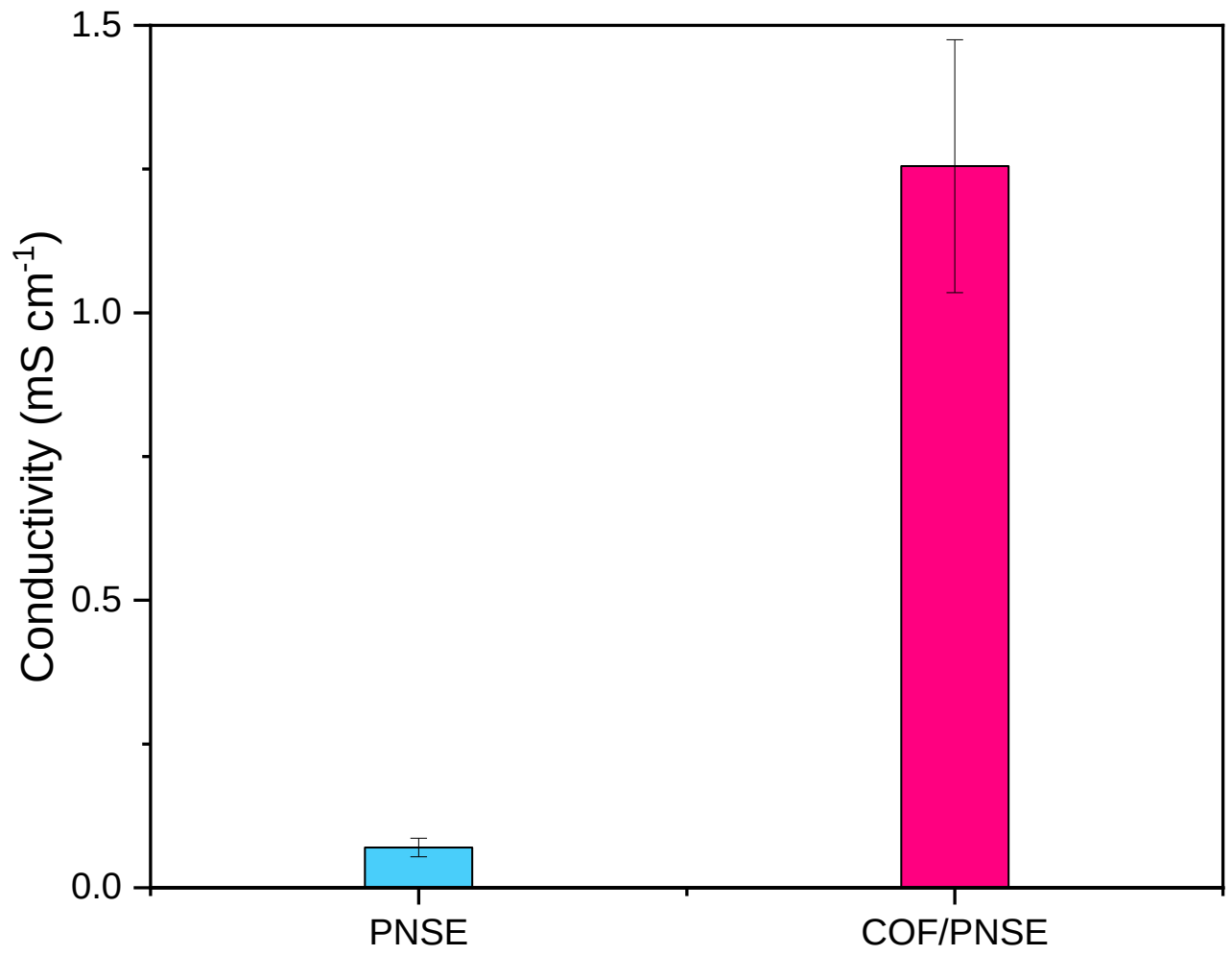

Fig. R2. The ion conductivity of COF/PNSE film of PNSE electrolyte.

Table R2. The cycling performance of Na||Na cells based on the solid state electrolytes reported previously.

Electrolyte	T (℃)	Current density (mA cm^{-2})	Capacity (mAh cm^{-2})	Cycling time (h)	Ref.
$Na_{3.5}Zr_{1.0}Ti_{0.5}Lu_{0.5}Si_2PO_{12}$	25	0.1	0.1	10000	EES, 2026, 19(3): 860-869.
POSS-	/	0.1	/	5150	AEM, 2018,

4PEG2K(NaE16)					8(27): 1801885.
NZMSP	/<br>30<br>30	0.5<br>0.1<br>0.3	/<br>0.1<br>0.1	3550<br>7000<br>4000	AM, 2025, 37(20): 2419190.
BSCPE	60	0.1	0.1	8800	AFM, 2024, 34(26): 2313496.
PEO-4SnF_2@PE	30	0.1	0.05	10800	AEM 16.5 (2026): e04169.
P(Na_m-EO_n)-PFPE	80	0.1	0.1	5000	AM, 2026: e23596.
FEC/SN-PCE	22	0.2	0.5	7200	Angewandte Chemie, 2024, 136(14): e202319427.

Table R3. The rate performance of Na solid full cells based on the solid state electrolytes reported previously.

Electrolyte	Cathode	Rate (C)	Cycle (n)	Retention (%)	References
GCSE(KN5P5)	NVFP	7	1000	82	Nano Energy 147 (2026) 111629
SPE-F	NVP	5	700	88.9	Adv. Energy Mater. 2026, 16, e03160
QSE	NVP	5	1000	83	Adv. Funct. Mater. 2023, 33, 2212866
PDFE-20	NVP	5	2000	93.6	Journal of Energy Chemistry, 2023, 77: 290-299.

GPE	NVP@C	200 mA g^{-1}	4000	86.6	Angewandte Chemie International Edition, 2025, 64(32): e202506349.
PCIE	NFM	60 mA g^{-1}	700	79.3	Nature Communications, 2025, 16(1): 2979.
		500 mA g^{-1}	1000	80	
FEC/SN-PCE	NVP	1C	1200	93	Angewandte Chemie, 2024, 136(14): e202319427.
PPNM	NVP@C	500 mA g^{-1}	2000	90.06%	Angewandte Chemie, 2025, 137(13): e202423075.

2) Second, the mechanistic interpretation—particularly regarding COF-directed formation of gradient SEI/CEI layers—is largely speculative. Most evidence is based on ex situ post-mortem characterization, and alternative explanations (e.g., dominant decomposition of fluorinated polymer and salt chemistry) are not critically discussed. As a result, cause–effect relationships are overstated.

[Author's Response]: We sincerely appreciate the reviewer's constructive critique regarding the mechanistic interpretation of the COF-directed gradient interface. We concede that in fluorinated polymer-based electrolytes, the decomposition of the polymer matrix and salt species (NaF formation) inevitably contributes to the SEI/CEI. Due to the complex nature of the polymer-COF composites, precisely isolating and "capturing" the transient interfacial behavior of the COF moieties in isolation is indeed inherently challenging. Nevertheless, we have addressed this by resolving the interfacial chemical evolution through

Cryo-TEM observations. Furthermore, by leveraging advanced operando characterization and mechanistic deconvolution, we demonstrate that the COF framework exerts a pivotal regulatory influence on the interfacial chemistry rather than being a mere "passive bystander". These combined real-time and high-resolution spatial evidences confirm that the COF skeleton actively dictates the formation of a robust, gradient interfacial layer.

(1) We have revised the manuscript to clarify that the "gradient interface" is not an isolated effect of the COF, but rather the result of a synergistic evolution involving the COF framework, the fluorinated polymer matrix, and the decomposition of sodium salts. To substantiate the existence and deterministic formation of this COF-induced gradient interface, we have integrated the physical "native-state" captured by Cryo-TEM with the chemical periodicity revealed by multi-scale depth profiling. Specifically, by employing Cryo-TEM (Figs. 4a-b, 6i-k), the thermally and beam-sensitive interfacial layers were vitrified and preserved at cryogenic temperatures, effectively bypassing the artifacts typically induced by electron-beam damage or solvent washing in conventional TEM. The observation of a uniform, dense, and amorphous/nanocrystalline hybrid structure confirms that this layer is an intrinsic "native-state" feature rather than a post-mortem artifact, directly addressing concerns regarding characterization reliability. Furthermore, the combined application of TOF-SIMS and XPS depth profiling (Figs. 4d-i, 6m-n) reveals a distinct spatial gradient of inorganic species (NaF, Na_2O) and organic fragments from the surface to the interior. Such highly ordered chemical heterogeneity cannot be attributed to the random decomposition of the polymer and salt, but serves as a direct manifestation of the COF-mediated nanoconfinement effect, which directs the orderly sedimentation of decomposition products. By cross-validating these multi-scale evidences, we have excluded the alternative explanation of "random decomposition" and confirmed the decisive role of the COF in governing the gradient interface formation. This elevates our interpretation from a speculative model to an

empirically validated framework supported by high-resolution physical and chemical evidence.

(2) To provide deeper insights, we conducted operando FTIR measurements specifically on Na||COF/PNSE||NNM full cells, focusing on the synergistic signal evolution of both the COF framework and the PVDF matrix (Fig. R3). By establishing a direct correlation between the structural fingerprints of the pristine TpPa-SO_3Na COF and the operando spectra of the composite electrolyte (Fig. S15), we have precisely deconstructed the dynamic regulatory mechanism at the interface. The results show that the COF framework peaks (1580-1620 cm^{-1}, assigned to C=C and aromatic vibrations) remain exceptionally stable, underscoring its robust mechanical and chemical integrity. Simultaneously, the reversible, periodic modulations of the S-O (1080 cm^{-1}) and C-N (1250 cm^{-1}) modes provide direct spectroscopic evidence of the COF's active role: the sulfonate groups anchor anions to facilitate a quasi-single-ion transport pathway, while the polar C-N sites function as a high-speed ion "conveyor belt" via dynamic coordination/de-coordination. Crucially, the characteristic signals of the PVDF matrix, particularly the symmetric and asymmetric stretching vibrations of -CF_2- groups (1150-1250 cm^{-1}), exhibit regular and highly reversible evolution throughout the cycling process. The absence of any disordered stray peaks typically associated with severe degradation demonstrates that the COF-driven nanoconfinement effect effectively suppresses the chaotic decomposition of the polymer matrix. This coordinated transport environment, stabilized by the synergy between COF and PVDF, significantly lowers the Na^+ migration barrier, thereby elevating our mechanistic interpretation to an empirically validated framework under real full-cell operating conditions.

In summary, our multi-scale and operando evidence confirms that the gradient interface is not a speculative byproduct of random decomposition, but a deterministic result of the COF framework actively regulating ion flux and stabilizing the PVDF/salt environment to create a robust, NaF-rich ion-

conductive buffer layer.

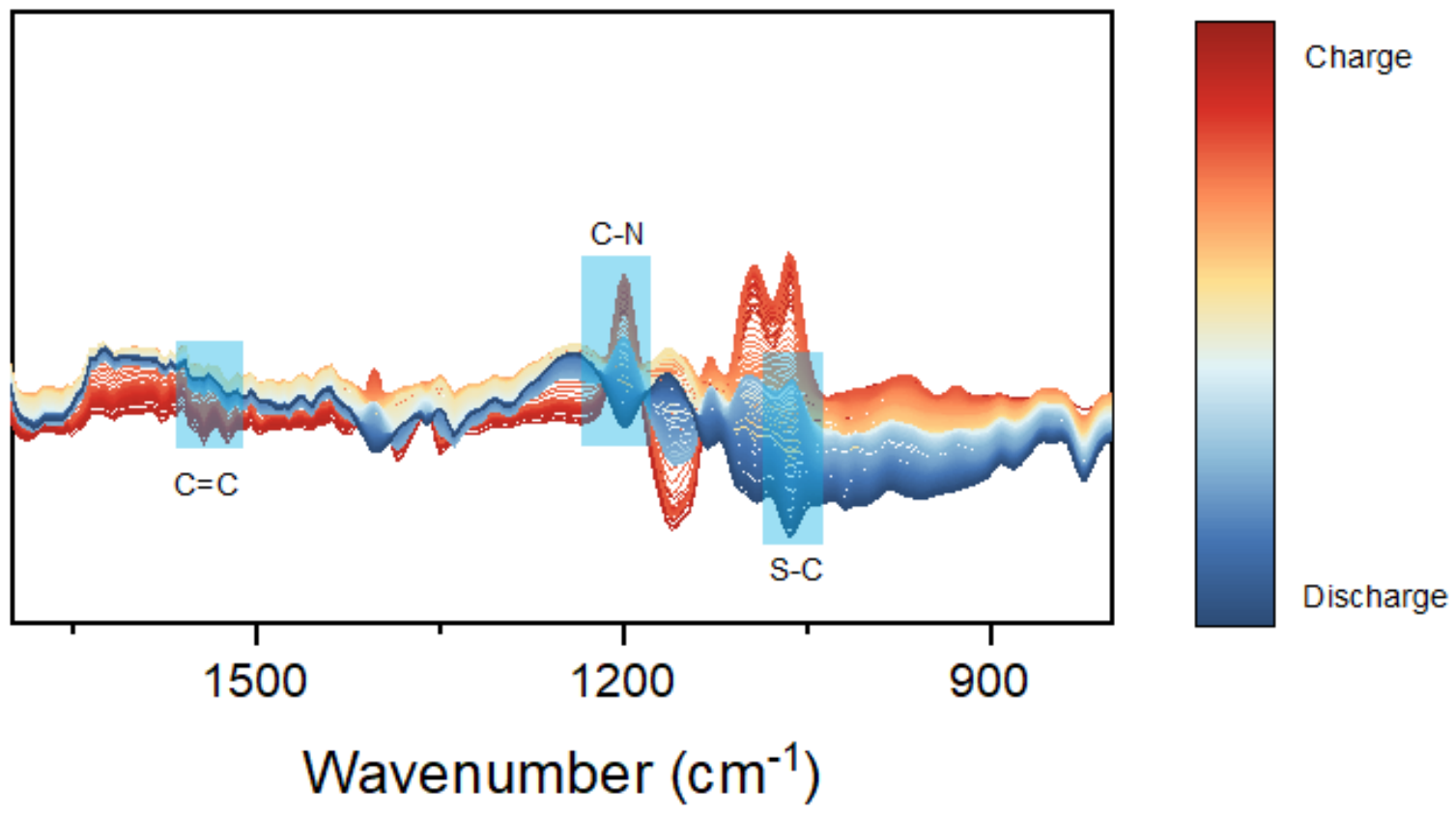

Fig. R3. Operando FT-IR measurements on Na||COF/PNSE||NNM full cells.

3) Third, the computational analysis (DFT and MD) lacks sufficient rigor and internal consistency. Simulation details are incomplete, conclusions are largely qualitative, and there are apparent inconsistencies in ion-species descriptions, which further undermine confidence in the mechanistic claims.

[Author's Response]: We sincerely appreciate the reviewer's critical comments on the computational section. We recognize that the initial presentation of our DFT and MD results lacked sufficient quantitative depth and technical detail. To address these concerns and reinforce the mechanistic claims, we have thoroughly updated the "Computational Methods" and "Results and Discussion" sections as follows:

(1) We have now provided a comprehensive breakdown of the simulation parameters in the Supplementary Information. To address these concerns, we have revised the manuscript to include a detailed description of the simulation parameters and clarified the distinct yet complementary roles of DFT and MD in our study. To ensure physical rigor across different scales, we maintained strict consistency in the treatment of charged species (Na^+ and $TFSI^-$):

In DFT calculations: The ionic species were modeled according to their formal oxidation states, with net charges set to +1 for Na^+ and -1 for $TFSI^-$. These settings were consistently applied during geometry optimization and

electronic property analysis to reflect the intrinsic electrostatic interactions.

In MD simulations: We employed the COMPASS III force field, which is specifically optimized for complex polymer/organic frameworks. In this force field, the net charges on Na^+ and $TFSI^-$ were explicitly defined as +1 and -1, respectively, ensuring that the long-range Coulombic interactions in the MD cell are aligned with the quantum mechanical charge descriptions in DFT.

(2) The reviewer's concern regarding "model inconsistency" is addressed by clarifying the different physical scales and objectives of the two methods:

The focus of DFT was to resolve the local electronic environment. Specifically, we calculated the Electrostatic Potential (ESP) surfaces of the PTC and COF frameworks to identify regions of negative potential. This allowed us to quantitatively analyze the adsorption/binding affinity of Na^+ onto various monomeric sites, providing a molecular basis for ion-trapping and desolvation.

The focus of MD was to capture the ensemble behavior of the system. We utilized MD to simulate the 3D spatial distribution of NaTFSI within the PTC/COF composite matrix, as well as to track the diffusion trajectories and calculate the self-diffusion coefficients (D_{Na^+}).

(3) By integrating these multi-scale insights where DFT defines the "active sites" and MD simulates the "transport efficiency", we provide a coherent theoretical framework that validates our experimental observations. All simulation details, including convergence criteria for DFT and equilibration protocols for MD, have been supplemented in the revised Supporting Information. As follows:

Quantum chemical calculations were conducted using density functional theory (DFT). All DFT computations were performed employing the DMol3 method [1]. This method utilizes rapidly convergent three-dimensional numerical integrations to compute matrix elements within the Ritz variational approach. A double numerical basis set with polarization functions on all atoms except hydrogen, known as the DND basis set, was utilized. For geometry optimizations, the projector augmented wave (PAW) method was used to

represent the ion–core electron interactions[2]. The valence electrons were represented with a plane wave basis set with an energy cutoff of 450 eV. Electronic exchange and correlation were described with the Perdew–Burke–Ernzerhof (PBE) functional[3]. DFT-D3 method was used to treat the van der Waals interaction [4]. The convergence criteria for the self-consistent electronic structure and geometry were set to 10^{-5} eV and 0.02 eV/Å, respectively.

The DFT-related calculations have been revised as follows:

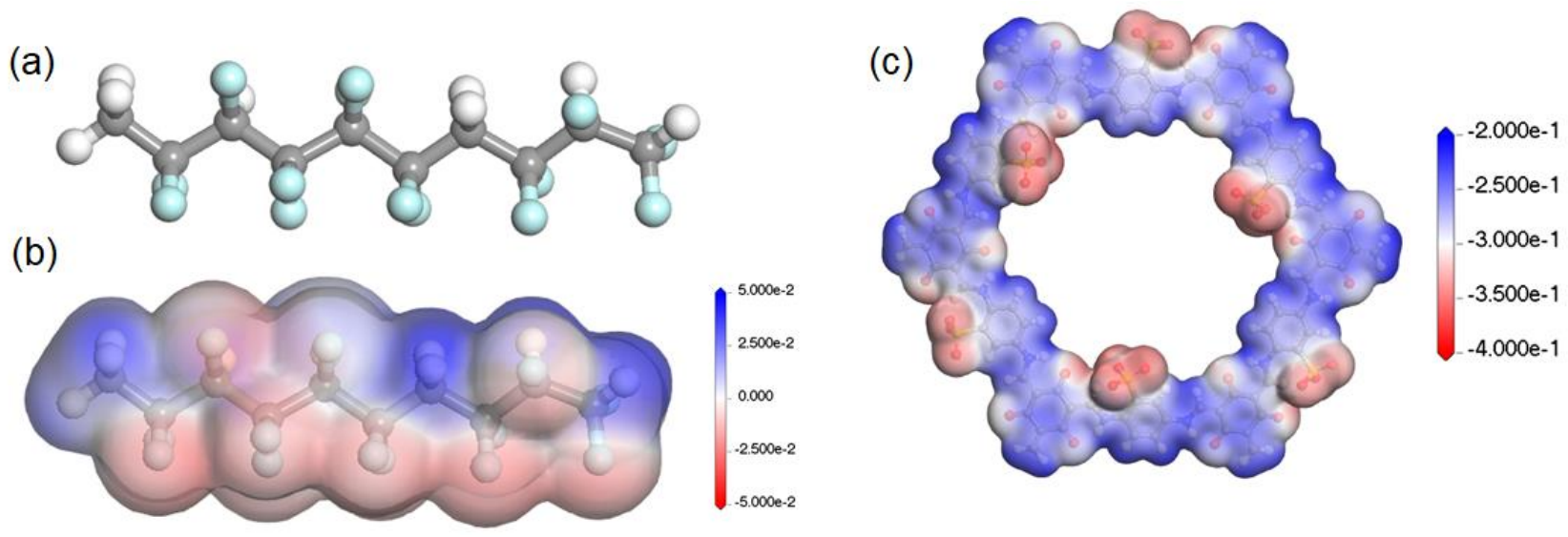

Fig. R4 (a) Molecular structure and (b) electrostatic potential distribution of PVDF-TrFE-CFE copolymer. (c) Electrostatic potential distribution of the COF framework.

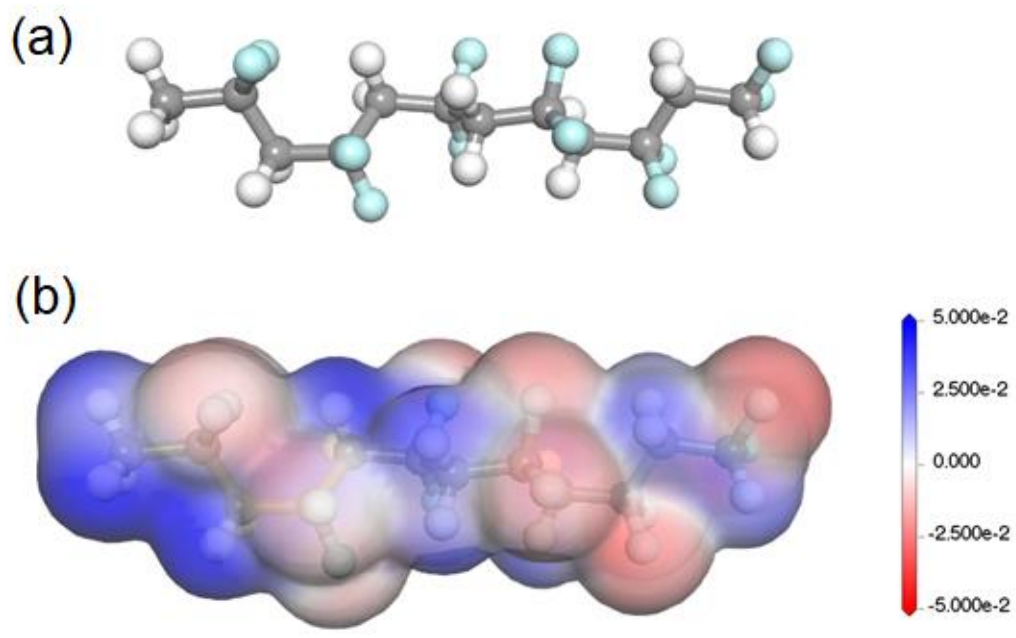

Fig. R5. (a) The ball-and-stick model of PVDF and (b) the electrostatic potential of PVDF.

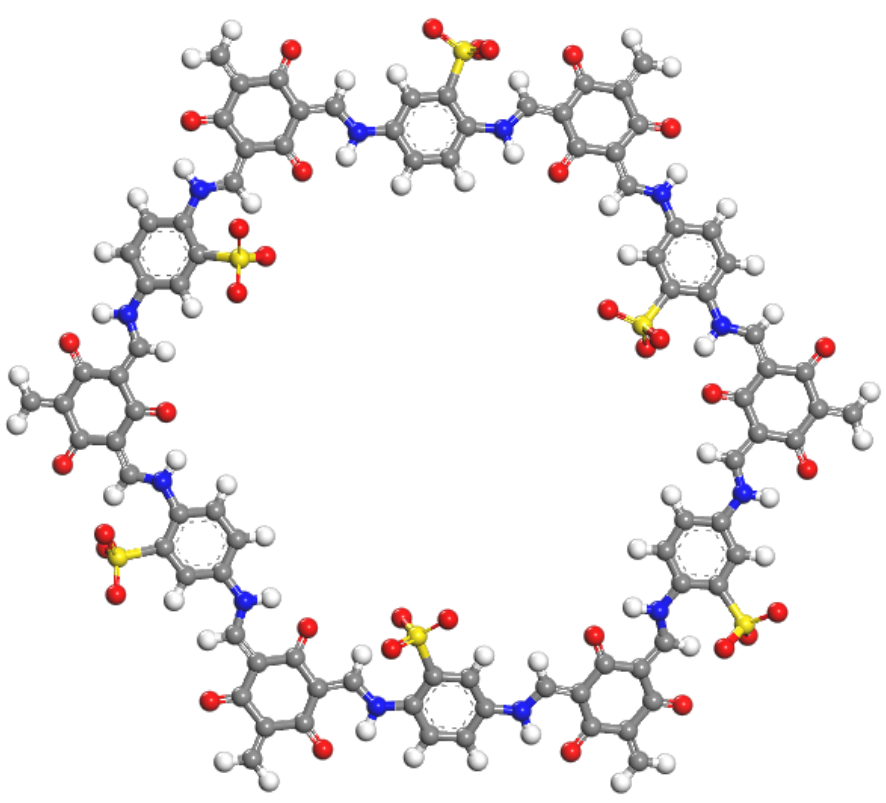

Fig. R6. The ball-and-stick model of TpPa-SO3-Na.

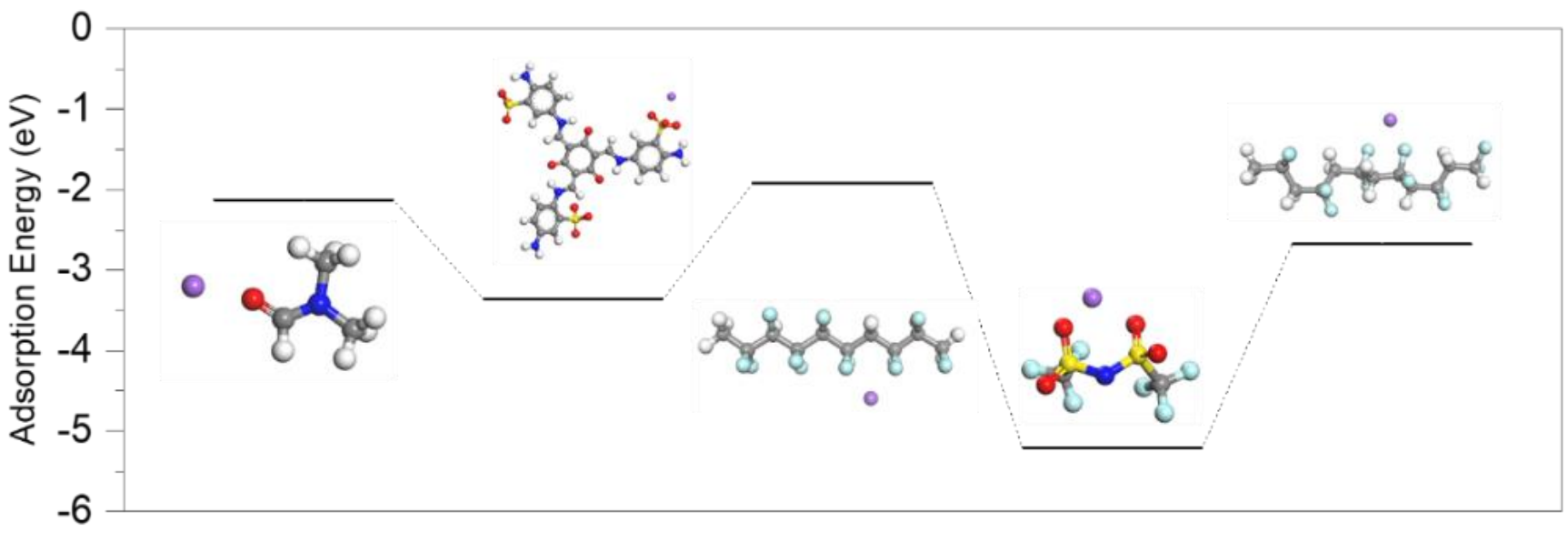

Fig. R7. Calculated adsorption energies of Na^+ with DMF, COF, $TFSI^-$, PTC, and PVDF.

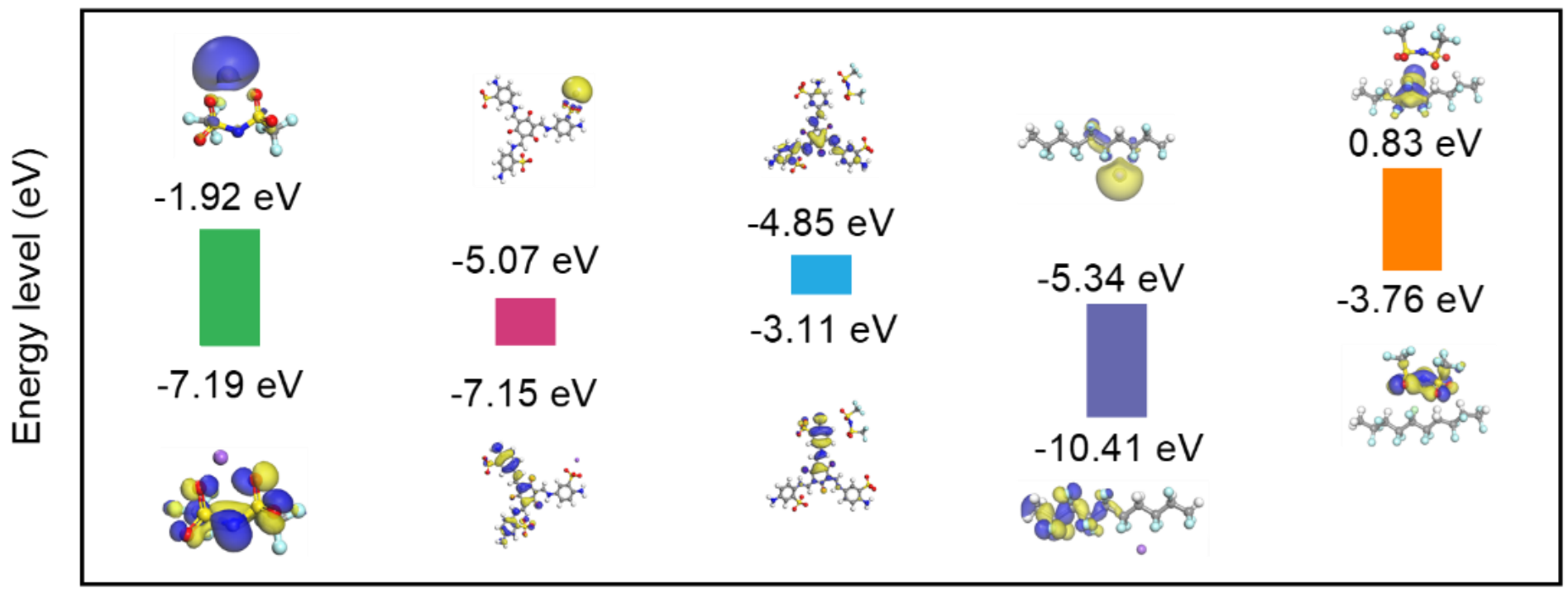

Fig. R8. The LUMO and HOMO energy values of Na^+-TFSI, Na^+-COF, $TFSI^-$-COF, Na^+-PTC, and TFSI-PTC.

References:

(1) Delley, B. DMol3 DFT studies: from molecules and molecular environments to surfaces and solids. *Computational Materials Science* **2000**, *17* (2), 122-126. DOI: https://doi.org/10.1016/S0927-0256(00)00008-2.

(2) Kresse, G.; Joubert, D. From ultrasoft pseudopotentials to the projector augmented-wave method. *Physical Review B* **1999**, *59* (3), 1758-1775. DOI: 10.1103/PhysRevB.59.1758.

(3) Perdew, J. P.; Burke, K.; Ernzerhof, M. Generalized gradient approximation made simple. *PHYSICAL REVIEW LETTERS* **1996**, *77* (18), 3865-3868, Article. DOI: 10.1103/PhysRevLett.77.3865.

(4) Grimme, S.; Antony, J.; Ehrlich, S.; Krieg, H. A consistent and accurate ab initio parametrization of density functional dispersion correction (DFT-D) for the

94 elements H-Pu. *The Journal of Chemical Physics* **2010**, *132* (15). DOI: 10.1063/1.3382344 (acccessed 6/22/2023).

4) Finally, the conceptual novelty is limited. COF–polymer composite electrolytes have been widely explored, and the manuscript does not clearly articulate what fundamentally distinguishes this system from prior reports beyond incremental performance improvements.

[Author's Response]: We have carefully addressed the reviewer's concerns by restructuring our response around three pivotal pillars that define the genuine innovation and scientific rigor of this study.

First, we would like to emphasize that the use of TpPa-SO_3Na COF in sodium-metal solid-state batteries is, based on our exhaustive literature search, a pioneering attempt that remains nearly absent from current reports (Table R1). Far from being a "passive filler", **this COF utilizes its specific anionic sulfonate groups to build a cooperative transport network within the PVDF-terpolymer matrix**. This atomic-scale design fundamentally reshapes the sodium-ion migration pathways, which is the true physical origin of the stable 10C ultra-high rate performance—a result that clearly transcends simple, incremental parameter tuning.

Secondly, the significance of this work is rooted in its transition **from qualitative guesswork to a rigorous, multi-dimensional evidentiary chain**. We deliberately moved away from traditional "post-mortem" characterization, instead utilizing operando FT-IR and operando DRT to track the live evolution of the solvation shell and interfacial charge-transfer kinetics. Furthermore, by employing Cryo-TEM to freeze the interphase in its "native state" and coupling this with XPS depth-profiling and TOF-SIMS, we have provided the first unambiguous visualization of a 3D chemical gradient. This comprehensive approach of **merging real-time kinetic tracking with atomic imaging** can offer a definitive explanation of how COF induces a gradient interphase, effectively filling the mechanistic vacuum left by previous studies.

Finally, we have successfully **pushed the boundaries of material innovation into the realm of practical engineering** by validating our electrolyte in Ah-level commercial pouch cells. We believe that achieving stable cycling in a large-format cell is the "litmus test" for any solid-state technology seeking to move beyond the laboratory. By synthesizing a new material system, high-resolution kinetic data, and practical scale-up evidence, our study does not just offer another composite electrolyte; it provides a technically viable and scientifically unique roadmap for the industrialization of solid-state sodium batteries. This practical breakthrough, we argue, significantly elevates the scientific impact and credibility of our findings.

Table R4. The performance of Na metal solid-state cells based on the COF materials reported previously.

Electrolyte	Functional groups	Ion conductivity (mS cm^{-1})	Full cell	References
–COO⁻-modified COF	–COO⁻	0.13	Na||$Na_3V_2(PO_4)_3$	Nature Communications, 2023, 14(1): 3066.
TFPPy-PyTTA-COF	SO_3^-	0.41	Na|QSSE-COF-SO_3Na|NFP	Energy Storage Materials, 2025, 77: 104192.
NaOOC-COF	–COO⁻	0.268	BQ | NaOOC-COF | Na	Nano Energy, 2022, 92: 106756.
B-COF	B-	0.528 (60℃)	NVP/Na	Advanced Functional Materials, 2024, 34(26): 2313496.
sp^2c-COF	sp^2	/	Na|COF|$NaTi_2$	Advanced

	carbon conjugated		$(PO_4)_3$	Energy Materials, 2023, 13(15): 2204083.
SD-COF-1	/	/	BQ | LiCON-3 | Li	ACS Energy Letters, 2020, 5(11): 3498-3506.

5) In summary, despite the extensive experimental data, the manuscript does not meet the standards required for publication due to concerns over data reliability, overinterpretation of mechanisms, and limited novelty. I therefore recommend rejection of the manuscript in its current form.

[Author's Response]: In summary, we respectfully but firmly contend that the concerns regarding data reliability, mechanistic interpretation, and novelty have been comprehensively addressed through our newly incorporated datasets and systematic deconstructions. Our response is grounded in three definitive evidentiary chains:

(1) We have addressed the concerns over "exceptionally high metrics" by providing rigorous statistical validation across multiple independent batches (Table R1). The reported conductivity (1.2 ms cm^{-1}) and ultra-long cycling (>6,700 h) are not isolated anomalies but reproducible results, now supported by standard deviation error bars and multi-cell verification. As substantiated in our benchmarking tables (Table R2-R3), these metrics are physically consistent with the cutting edge of sodium solid-state chemistry and are further validated by the inherent high-rate capability of our P2-NNM cathode (200C in liquid batteries).

(2) We have moved beyond "qualitative guesswork" by establishing a direct cause-and-effect relationship between the COF framework and the gradient interface. By employing Cryo-TEM, we resolved the "native-state" of the

interfacial architecture, bypassing beam-induced artifacts. This was complemented by Operando FT-IR and Operando DRT, which provided real-time spectroscopic and kinetic evidence of the COF's active role in anchoring anions and facilitating ion flux. The observed 3D chemical gradient (via XPS/TOF-SIMS depth profiling) confirms that the interface is a deterministic product of COF-mediated nanoconfinement rather than random decomposition, effectively filling the mechanistic vacuum identified by the reviewer.

(3) Finally, we emphasize that our work is distinguished from prior reports by its transition from material design to Ah-level commercial pouch cell validation. The pioneer use of TpPa-SO_3Na COF combined with the demonstration of stable operation in large-format cells serves as a "litmus test" for the technical viability of this system. This bridge from atomic-scale engineering to industrial-scale application significantly elevates the scientific positioning of our manuscript beyond mere incremental performance tuning.

We believe that the integration of these high-resolution datasets and the robust evidentiary chain resolves the initial concerns regarding mechanistic uncertainty and reliability. Our study provides a scientifically unique and technically viable roadmap for the industrialization of solid-state sodium batteries, meeting the rigorous standards for publication.

We want to thank the reviewers for the valuable comments and suggestions, which are helpful for improving the quality of this manuscript.
