# Supplementary material for: Engineering ion migration and interface chemistry via covalent organic framework-enhanced polymer electrolytes for fast-charging sodium solid-state batteries
Source: Nat Commun. 2026 Jul 20;17:8850. doi: 10.1038/s41467-026-75417-7 (PMC13500708; doi:10.1038/s41467-026-75417-7)
Supplement: Supplementary file 3 [file 41467_2026_75417_MOESM1_ESM.docx]

Supplementary Information for

**Modulating Ion Migration and Interface Chemistry via Covalent Organic Framework-Boosted Polymer Electrolytes toward Fast-Charging Sodium Solid-State Batteries**

Yongbiao Mu‡,^1,2^ Chen Li‡,^3^ Jun Huang‡,^3^ Xiaoqian Xu‡,^1,2^ Yichun Wang,^1,2^ Quanyan Man,^1,2^ Zhiyu Zou,^1,2^ Meisheng Han,^1,2^ Qing Zhang,^1,2^ Guobin Zhang,*^4^ Lei Wei,*^1,2^ Baohua Li,*^5^ and Lin Zeng*^1,2^

^1^Shenzhen Key Laboratory of Advanced Energy Storage, Department of Mechanical and Energy Engineering, Southern University of Science and Technology, Shenzhen 518055, China

^2^SUSTech Energy Institute for Carbon Neutrality, Southern University of Science and Technology, Shenzhen 518055, China

^3^Department of Chemical and Biological Engineering, The Hong Kong University of Science and Technology, Hong Kong SAR, 999077 China

^4^College of New Materials and New Energies, Shenzhen Technology University, Shenzhen 518055, China

^5^Institute of Materials Research, Tsinghua Shenzhen International Graduate School, Tsinghua University Shenzhen 518055, China

‡These authors contributed equally.

E-mail: zhangguobin@sztu.edu.cn (Guobin Zhang); [weil@sustech.edu.cn](mailto:weil@sustech.edu.cn) (Lei Wei); libh@sz.tsinghua.edu.cn (Baohua Li); zengl3@sustech.edu.cn (Lin Zeng)

**Supplementary Figures**

**Figure S1.** Synthesis process and molecular structures of the raw materials and products of TpPa-SO_3_Na COF material.

**Figure** **S2.** XRD of the raw materials used for COF synthesis and the final COF material. (a) Tp, (b) Pa-SO_3_H, (c) TpPa-SO_3_H, and (d) TpPa-SO_3_-Na.

**Figure S3.** SEM image of TpPa-SO_3_-Na COF.

**Figure S4.** SEM mapping images of TpPa-SO_3_-Na. a) Mixed mapping, b) C, c) O, d) S, e) Na, f) N elements.

**Figure S5.** High-resolution TEM image of the TpPa-SO_3_-Na.

**Figure S6.** The XPS full spectrum of TpPa-SO_3_-Na COF.

**Figure S7.** The pore size distribution of TpPa-SO_3_-Na.

**Figure S8.** Optical image of large-area COF/PNSE electrolyte film.

**Figure S9.** SEM images of COF/PNSE electrolyte. (a) High-magnification SEM, and (b) low-magnification SEM.

**Figure** **S10.** SEM image of the PNSE electrolyte film.

**Figure S11.** Cross-sectional SEM image of the PNSE electrolyte film.

**Figure S12.** EDS mapping image (top-view) of the COF/PNSE electrolyte, including C, N, O, F, S, Na elements.

**Figure S13.** EDS mapping image (cross sectional-view) of the PNSE electrolyte, including C, N, O, F, S, Na, and Cl elements.

**Figure S14.** TGA curves of TpPa-SO_3_-Na and COF/PNSE electrolyte films.

**Figure S15.** FTIR spectra of PNSE and COF/PNSE electrolytes.

**Figure S16.** Stress-strain curves of PNSE and COF/PNSE electrolytes.

**Figure S17.** EIS spectra of PNSE electrolyte at different temperatures from 25 to 80 °C.

**Figure S18.** Ionic conductivity of PNSE electrolyte at different temperatures from 25 to 80 °C.

**Figure S19.** The (a) i-t curve and (b) EIS spectra before and after polarization of Na||Na cells using COF/PNSE electrolyte at room temperature.

**Figure S20.** (a) The ball-and-stick model of PVDF and (b) the electrostatic potential of PVDF.

**Figure S21.** The ball-and-stick model of TpPa-SO_3_-Na.

**Figure S22.** The structural diagram of TpPa-SO_3_-Na in MD simulations.

**Figure S23.** Simulation snapshots of the Na⁺ migration in the PNSE system at room temperature from 0.25 ns to 2 ns. (a) 0.25 ns, (b) 0.50 ns, (c) 1.0 ns, (d) 1.5 ns, (e) 2.0 ns.

**Figure S24.** The deposition and stripping curve of Na||Cu battery with different electrolyte. (a) COF/PNSE and (b) PNSE electrolytes at 1 mA cm^-2^ and 1 mAh cm^-2^.

**Figure S25.** Comparison of polarization voltage at different current densities using two different electrolytes in CCD testing.

**Figure S26.** Comparison of polarization voltage at different current densities for Na||Na symmetric cell using PNSE and COF/PNSE electrolytes.

**Figure S27.** The deposition and stripping curve of Na||Na cells at different time. (a) 0~10 h and (b) 400~410 h at 1 mA cm^-2^ and 0.5 mAh cm^-2^.

**Figure S28.** In-situ EIS spectra of Na||Na symmetric cell under different deposited Na using different electrolytes. (a) PNSE and (b) COF/PNSE electrolytes.

**Figure S29.** HOMO/LUMO energy levels and orbitals of each molecule of Na^+^-TFSI, Na^+^-COF, TFSI^-^-COF, Na^+^-PTC, and TFSI^-^-PTC, respectively.

**Figure S30.** Cryo-TEM images of SEI on Na-anode surface formed by PNSE electrolyte at (a) low-magnification and (b) high-resolution.

**Figure S31.** 3D image of TOF-SIMS of (a) F_2_^-^ and (b) NaO^-^ using PNSE electrolyte.

**Figure S32.** XPS profiles of Na-anode cycled in Na|PNSE|Na cells. (a) Na 1s, (b) F 1s, and (c) O 1s spectra.

**Figure S33.** SEM images of cycled Na metal after 100 times repeated plating and stripping using PNSE electrolyte. (a) high-magnification and (b) low-resolution.

**Figure S34.** AFM 3D images of the sodium metal surface roughness after 500 hours of cycling: (a) using the PNSE electrolyte; (b) using the COF/PNSE electrolyte.

**Figure S35.** SEM images of deposited Na metal with the different capacity using COF/PNSE electrolyte. (a) 1 mAh, (b) 2 mAh, and (c) 3 mAh.

**Figure S36.** SEM images of NNM electrode:(a) low-magnification and (b) high-resolution.

**Figure S37.** The charge-discharge curves of Na||SE||NNM batteries at different rates in the 2.0~4.0 V range using (a) PNSE and (b) COF/PNSE electrolytes.

**Figure S38.** The charge-discharge curves of Na||COF/PNSE||NNM batteries in the 2.0~4.0 V range at 1C from 1^st^ cycle to 1000^th^ cycle.

**Figure S39.** *In-situ* EIS spectra of Na||SE||NNM symmetric cell using (a) PNSE and (b) COF/PNSE electrolytes.

**Figure S40**. SEM images of cycled hard carbon electrode:(a) low-magnification and (b) high-resolution.

**Figure S41.** FIB-SEM images of cycled NNM electrode with COF/PNSE electrolyte. (a) low-magnification and (b) high-resolution.

**Figure S42.** FIB-SEM images of cycled NNM electrode with PNSE electrolyte. (a) low-magnification and (b) high-resolution.

**Fig. S43**. Operando FT-IR measurements on Na||COF/PNSE||NNM batteries.

**Figure S44.** EDS images of cycled NNM electrode with COF/PNSE electrolyte. (a) C, (b) F, (c) O, (d) Mn, (e) Ni, and (f) Na elements.

**Figure S45.** XPS depth-dependent elemental distribution at the cathode interface. (a) C 1s, (b) F 1s, and (c) O 1s

**Figure S46.** 2D TOF-SIMS images of (a) C^-^, (b) O^-^ , and (c) F^-^ at the cathode/electrolyte interface.

**Table S1.** The comparison of Na||Na symmetric cell electrochemical performance for solid-state/hydrogel electrolytes reported in the literature.

| Electrolytes | Current density (mA cm^-2^) | Lifetime  (h) | Ref. |
| --- | --- | --- | --- |
| POSS-4PEG2K(NaE16) | 0.5 | 3550 | ^1^ |
| NaFSI/PolyIL | 0.5 | 100 | ^2^ |
| PEGMEM-co-SSS@ZIF-8 | 0.5 | 220 | ^3^ |
| PBAGPE | 0.2 | 980 | ^4^ |
| NZSP/PVDF-HFP | 0.2 | 400 | ^5^ |
| ETPTA–NaClO_4_–QSSE | 0.1 | 1000 | ^6^ |
| AMPSN | 0.4 | 400 | ^7^ |
| PEO@LM&SO | 0.2 | 800 | ^8^ |
| DSPE | 0.1 | 1000 | ^9^ |
| PBMP | 0.5 | 1100 | ^10^ |
| ATFPE | 0.1 | 600 | ^11^ |
| c-GPE | 0.1 | 800 | ^12^ |
| FEC/SN-PCE | 0.5 | 1800 | ^13^ |
| PSB60-GPE | 0.5 | 800 | ^14^ |
| BSCPE | 0.5 | 200 | ^15^ |
| PPNM | 0.23 | 500 | ^16^ |
| sp^2^ C-COF | 0.5 | 2000 | ^17^ |
| QSSE-COF-SO_3_Na | 0.2 | 830 | ^18^ |
| NaOOC-COF | 0.05 | 700 | ^19^ |
| TPDBD-CNa-QSSE | 0.01 | 900 | ^20^ |
| **COF/PNSE** | **0.1** | **6750** | **This work** |
|  | **1.0** | **418** |  |

**Table S2.** The comparison of electrochemical performance for Na||SE||NFM(NNM) batteries with solid-state/hydrogel electrolytes reported in the literature.

| Samples | Rate (C) | Cycle | Retention (%) | Temperature (℃) | Ref. |
| --- | --- | --- | --- | --- | --- |
| PVC-CPE | 0.2C | 250 | 86.8 | 25 | ^21^ |
| B-CPE | 0.2C | 320 | 80.1 | 40 | ^22^ |
|  | 3C | 700 | 80.4 | 10 |  |
| B-PCPE | 0.1C | 120 | 80.1 | 25 | ^23^ |
| HP-GPE | 120 mA/g | 400 | 70 | 25 | ^24^ |
| PAPE SSEs | 0.2C | 100 | 91.1 | 25 | ^25^ |
|  | 1.0C | 250 | ~70 | 25 |  |
| **COF/PNSE** | **1.0C** | **1000** | **77.2** | **30** | **(This work)** |
|  | **5.0C** | **2000** | **80.2** | **30** |  |
|  | **10.0C** | **2000** | **83.5** | **30** |  |

**Supplementary References**

1.	Zheng Y, Pan Q, Clites M, Byles BW, Pomerantseva E, Li CY. High-Capacity All-Solid-State Sodium Metal Battery with Hybrid Polymer Electrolytes. *Advanced Energy Materials* **8**, 1801885 (2018).

2.	Chen F, Wang X, Armand M, Forsyth M. Cationic polymer-in-salt electrolytes for fast metal ion conduction and solid-state battery applications. *Nature Materials* **21**, 1175-1182 (2022).

3.	Zhang J*, et al.* Confining Polymer Electrolyte in MOF for Safe and High-Performance All-Solid-State Sodium Metal Batteries. *Angewandte Chemie International Edition* **63**, e202318822 (2024).

4.	Zhang W*, et al.* In-situ Polymerized Gel Polymer Electrolytes with High Room-Temperature Ionic Conductivity and Regulated Na+ Solvation Structure for Sodium Metal Batteries. *Advanced Functional Materials* **32**, 2201205 (2022).

5.	Ling W*, et al.* A Flexible Solid Electrolyte with Multilayer Structure for Sodium Metal Batteries. *Advanced Energy Materials* **10**, 1903966 (2020).

6.	Wen P*, et al.* Photopolymerized Gel Electrolyte with Unprecedented Room-Temperature Ionic Conductivity for High-Energy-Density Solid-State Sodium Metal Batteries. *Advanced Energy Materials* **11**, 2002930 (2021).

7.	Dong X, Zhang Y, You Z, Chen Y, Wu X, Wen Z. Polyanion-Induced Single Na-Ion Polymer Electrolytes for Ultra-Stable Sodium Metal Batteries. *Advanced Functional Materials* **34**, 2405931 (2024).

8.	Suo J*, et al.* A Novel Super-Toughness and Self-Healing Solid Polymer Electrolyte for Solid Sodium Metal Batteries. *Advanced Materials* **36**, 2409587 (2024).

9.	Law HM*, et al.* A hybrid dual-salt polymer electrolyte for sodium metal batteries with stable room temperature cycling performance. *Energy Storage Materials* **46**, 182-191 (2022).

10.	Zhang Q*, et al.* Tertiary-Amine Based Network Polymer Electrolyte for Improving the Cyclic Stability of Na Metal Batteries with Large Capacity. *Advanced Energy Materials* **14**, 2303791 (2024).

11.	Guo J*, et al.* Achieving Ultra-Stable All-Solid-State Sodium Metal Batteries with Anion-Trapping 3D Fiber Network Enhanced Polymer Electrolyte. *Small* **19**, 2206740 (2023).

12.	Yuan Y*, et al.* Reconfiguring Polymer Chain for Regulating Na+ Solvation Structure in a Gel Polymer Electrolyte toward Sodium Metal Batteries. *ACS Energy Letters* **10**, 2007-2016 (2025).

13.	Zou P, Wang C, He Y, Xin HL. Making Plasticized Polymer Electrolytes Stable Against Sodium Metal for High-Energy Solid-State Sodium Batteries. *Angewandte Chemie International Edition* **63**, e202319427 (2024).

14.	Lai H*, et al.* In situ generated composite gel polymer electrolyte with crosslinking structure for dendrite-free and high-performance sodium metal batteries. *Energy Storage Materials* **54**, 478-487 (2023).

15.	Guo J*, et al.* Boosting Selective Na+ Migration Kinetics in Structuring Composite Polymer Electrolyte Realizes Ultrastable All-Solid-State Sodium Batteries. *Advanced Functional Materials* **34**, 2313496 (2024).

16.	Tian W, Lin G, Yuan S, Jin T, Wang Q, Jiao L. Competitive Coordination and Dual Interphase Regulation of MOF-Modified Solid-State Polymer Electrolytes for High-Performance Sodium Metal Batteries. *Angewandte Chemie International Edition* **64**, e202423075 (2025).

17.	Kang T*, et al.* Dendrite-Free Sodium Metal Anodes Via Solid Electrolyte Interphase Engineering With a Covalent Organic Framework Separator. *Advanced Energy Materials* **13**, 2204083 (2023).

18.	Kang T*, et al.* Ionic covalent organic frameworks-based electrolyte enables fast Na-ion diffusion towards quasi-solid-state sodium batteries. *Energy Storage Materials* **77**, 104192 (2025).

19.	Zhao G*, et al.* COFs-based electrolyte accelerates the Na+ diffusion and restrains dendrite growth in quasi-solid-state organic batteries. *Nano Energy* **92**, 106756 (2022).

20.	Yan Y*, et al.* Bioinspired design of Na-ion conduction channels in covalent organic frameworks for quasi-solid-state sodium batteries. *Nature Communications* **14**, 3066 (2023).

21.	Chen S*, et al.* Poly(vinylene carbonate)-Based Composite Polymer Electrolyte with Enhanced Interfacial Stability To Realize High-Performance Room-Temperature Solid-State Sodium Batteries. *ACS Applied Materials & Interfaces* **11**, 43056-43065 (2019).

22.	Chen S, Feng F, Che H, Yin Y, Ma Z-F. High performance solid-state sodium batteries enabled by boron contained 3D composite polymer electrolyte. *Chemical Engineering Journal* **406**, 126736 (2021).

23.	Chen S, Feng F, Yin Y, Lizo X, Ma Z. Plastic crystal polymer electrolytes containing boron based anion acceptors for room temperature all-solid-state sodium-ion batteries. *Energy Storage Materials* **22**, 57-65 (2019).

24.	Hu G, Xu Z, Wang Z. A fluorine-rich and flame-retardant gel polymer electrolyte enables thermal polymerization of fluorinated acrylates for stable quasi-solid-state Na-ion batteries. *Chemical Communications* **61**, 4852-4855 (2025).

25.	Liu C*, et al.* Scalable and Ultrathin Dual Entangled Network Polymer Electrolytes for Safe Solid-State Sodium Batteries. *Angewandte Chemie International Edition* **64**, e202505938 (2025).
